# Exploring New Physics Frontiers Through Numerical Relativity

**DOI:** 10.1007/lrr-2015-1

**Published:** 2015-09-21

**Authors:** Vitor Cardoso, Leonardo Gualtieri, Carlos Herdeiro, Ulrich Sperhake

**Affiliations:** 1 CENTRA, Departamento de Física, Instituto Superior Técnico, Universidade de Lisboa, Avenida Rovisco Pais 1, 1049 Lisboa, Portugal; 2 Perimeter Institute for Theoretical Physics, Waterloo, Ontario N2L 2Y5 Canada; 3 Dipartimento di Fisica, Università di Roma “La Sapienza” & Sezione INFN Roma1, P.A. Moro 5, 00185 Roma, Italy; 4 Departamento de Física da Universidade de Aveiro and CIDMA, Campus de Santiago, 3810-183 Aveiro, Portugal; 5 DAMTP, Centre for Mathematical Sciences, University of Cambridge, Wilberforce Road, Cambridge, CB3 0WA UK

**Keywords:** Gravitation, Numerical methods, Black holes, Extensions of the standard model, Alternative theories of gravity, Extra dimensions, Trans-Planckian scattering

## Abstract

The demand to obtain answers to highly complex problems within strong-field gravity has been met with significant progress in the numerical solution of Einstein’s equations — along with some spectacular results — in various setups.

We review techniques for solving Einstein’s equations in generic spacetimes, focusing on fully nonlinear evolutions but also on how to benchmark those results with perturbative approaches. The results address problems in high-energy physics, holography, mathematical physics, fundamental physics, astrophysics and cosmology.

## Prologue

*“Wir müssen wissen, wir werden wissen.”* (We must know, we will know.)

— D. Hilbert, Address to the Society of German Scientists and Physicians, Königsberg (September 08, 1930).

One century of peering into Einstein’s field equations has given us elegant and simple solutions, and shown how they behave when slightly displaced from equilibrium. We were rewarded with a beautiful mathematical theory of black holes (BHs) and their perturbations, and a machinery that is able to handle all weak-field phenomena. After all, one hundred years is not a very long time to understand a theory with such conceptual richness. Left behind, as an annoying nuisance, was the problem of dynamical strong-field effects such as the last stages of BH mergers.

In the last few decades, it gradually became clear that analytical or perturbative tools could only go so far: gravitational-wave (GW) detectors were promising to see the very last stages of BH-binary inspirals; fascinating developments in String/M theory (SMT) were hinting at a connection between gauge theories and strong gravity effects; extensions of the standard model of particle physics were conjecturing the existence of extra dimensions, which only gravity had access to, and were predicting BH formation at accelerators! This, and more, required the ability to solve Einstein’s equations (numerically) in full generality in the nonlinear regime. The small “annoying nuisance” rapidly grew to become an elephant in the room that had to be tamed.

But necessity is the mother of inventions. In 2005, several groups achieved the first long-term stable evolutions of BH-binaries in four-dimensional, asymptotically flat spacetimes, starting a phase transition in the field. It is common to refer to such activity — numerically solving Einstein’s equations

1 $${R_{\mu \nu}} - {1 \over 2}R{g_{\mu \nu}} = {{8\pi G} \over {{c^4}}}{T_{\mu \nu}},$$

or extensions thereof — as “numerical relativity” (NR). In practice, *any* numerical procedure is a means to an end, which is *to know*. In this sense, NR is a gray area which could lie at the intersection between numerical analysis, general relativity (GR) and high-energy physics. Many different numerical techniques have been used to solve the field equations in a variety of contexts. NR usually entails solving the full set of nonlinear, time-dependent Einstein-type equations.

This is a review on NR. We will cover all aspects of the main developments in the last decade, focusing for the most part on evolutions of BH spacetimes. The numerical resolution of Einstein’s equations in a computer has a five-decade long history and many important ingredients. In fact, NR is sufficiently complex that a number of outstanding review works have already been dedicated to specific aspects, like construction of initial data, finding horizons in numerical spacetimes, evolving the field equations in the presence of matter, etc. We will not attempt to cover these in any detail; we refer the reader to the relevant section of *Living Reviews*^1^ for this and to textbooks on the subject at large [21, 79, 111, 364]. The present work is mostly intended to make the reader familiar with new developments, which have not and could not have been covered in those works, given the pace at which the field is evolving.

**Figure 1:**
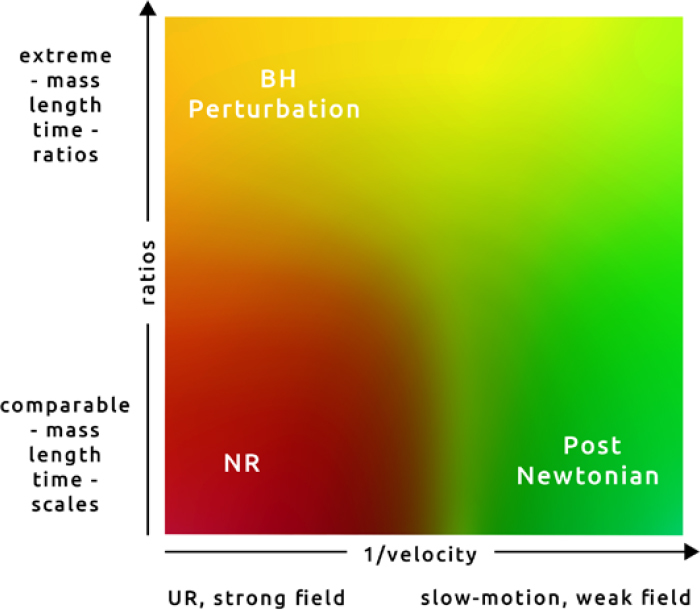
Range of various approximation tools (”UR” stands for ultra-relativistic). NR is mostly limited by resolution issues and therefore by possible different scales in the problem.

A few words about the range and applicability of NR methods are in order, as they help clarify the content of this review work. NR is but one, albeit important and complex, tool that helps us to get through solving and understanding certain processes. Traditionally, the two-body problem in GR for instance, was approached via a slow-motion, large separation post-Newtonian expansion. The PN expansion breaks down when the distances between the bodies are small and the velocities are large. BH perturbation theory on the other hand, can handle the two-body problem for any separation and velocity, but as long as there is a decoupling of mass scales, i.e, one of the objects must be much more massive than the other. The remaining is NR turf: large velocities, small separations, strong field and similar masses. This is depicted in Figure 1, which we have extended to allow for generic situations. NR methods typically break down (due to large computational requirements) when there are extremely different scales in the problem, i.e., when extremely large or small dimensionless quantities appear. For instance, the two-body problem in GR can be handled for a relatively short timescale, *and* as long as the two bodies do not have extreme mass ratios. In spacetimes with other lengthscales, for instance AdS, NR encounters difficulties when the binary lengthscale is much smaller than the AdS lengthscale for example. While such simulations can in principle be done, they may not capture the relevant physics associated with the AdS boundary.

To conclude this discussion, neither NR nor perturbative techniques are paradisiac islands in isolation; input and interplay from and with other solutions is often required. As such, we will also discuss in some detail some of the perturbative tools and benchmarks used in the field.

NR has been crucial to answer important questions in astrophysics, GW physics, high-energy physics and fundamental physics, and as such we thought it convenient — and fun — to start with a timeline and main theoretical landmarks that have stimulated research in the last years. This will hopefully help the reader getting started by understanding which are the main breakthroughs and where exactly do we stand.

## Milestones

Numerical solving is a thousand-year-old art, which developed into modern numerical analysis several decades ago with the advent of modern computers and supercomputers. For a compelling account of the early history of numerical analysis and computing we refer the reader to Goldstine [359, 360].

It is impossible to summarize all the important work on the subject in this review, but we find it instructive to list a chronogram of several relevant milestones taking us to 2014, in the context of GR. The following is a list — necessarily incomplete and necessarily biased — of works which, in our opinion, have been instrumental to shape the evolution of the field. A more complete set of references can be found in the rest of this review.

- 1910 — The analysis of finite difference methods for PDEs is initiated with Richardson [648].
- 1915 — Einstein develops GR [293, 294].
- 1916 — Schwarzschild derives the first solution of Einstein’s equations, describing the gravitational field generated by a point mass. Most of the subtleties and implications of this solution will only be understood many years later [687].
- 1917 — de Sitter derives a solution of Einstein’s equations describing a universe with constant, positive curvature A. His solution would later be generalized to the case Λ < 0 [255].
- 1921, 1926 — In order to unify electromagnetism with GR, Kaluza and Klein propose a model in which the spacetime has five dimensions, one of which is compactified on a circle [463, 476].
- 1928 — Courant, Friedrichs and Lewy use finite differences to establish existence and uniqueness results for elliptic boundary-value and eigenvalue problems, and for the initial-value problem for hyperbolic and parabolic PDEs [228].
- 1931 — Chandrasekhar derives an upper limit for white dwarf masses, above which electron degeneracy pressure cannot sustain the star [193]. The Chandrasekhar limit was subsequently extended to NSs by Oppenheimer and Volkoff [590].
- 1939 — Oppenheimer and Snyder present the first dynamical collapse solution within GR [589].
- 1944 — Lichnerowicz [515] proposes the conformal decomposition of the Hamiltonian constraint laying the foundation for the solution of the initial data problem.
- 1947 — Modern numerical analysis is considered by many to have begun with the influential work of John von Neumann and Herman Goldstine [763], which studies rounding error and includes a discussion of what one today calls scientific computing.
- 1952 — Choquet-Bruhat [327] shows that the Cauchy problem obtained from the spacetime decomposition of the Einstein equations has locally a unique solution.
- 1957 — Regge and Wheeler [641] analyze a special class of gravitational perturbations of the Schwarzschild geometry. This effectively marks the birth of BH perturbation theory, even before the birth of the BH concept itself.
- 1958 — Finkelstein understands that the *r* = 2*M* surface of the Schwarzschild geometry is not a singularity but a horizon [320]. The so-called “golden age of GR” begins: in a few years there would be enormous progress in the understanding of GR and of its solutions.
- 1961 — Brans and Dicke propose an alternative theory of gravitation, in which the metric tensor is non-minimally coupled with a scalar field [128].
- 1962 — Newman and Penrose [575] develop a formalism to study gravitational radiation using spin coefficients.
- 1962 — Bondi, Sachs and coworkers develop the characteristic formulation of the Einstein equations [118, 667].
- 1962 — Arnowitt, Deser and Misner [47] develop the canonical 3 + 1 formulation of the Einstein equations.
- 1963 — Kerr [466] discovers the mathematical solution of Einstein’s field equations describing rotating BHs. In the same year, Schmidt identifies the first quasar (quasi-stellar radio source) [681]. Quasars are now believed to be supermassive BHs, described by the Kerr solution.
- 1963 — Tangherlini finds the higher-dimensional generalization of the Schwarzschild solution [740].
- 1964 — Chandrasekhar and Fock develop the post-Newtonian theory [194, 325].
- 1964 — First documented attempt to solve Einstein’s equations numerically by Hahn & Lindquist [385]. Followed up by Smarr & Eppley about one decade later [710, 311].
- 1964 — Seymour Cray designs the CDC 6600, generally considered the first supercomputer. Speeds have increased by over one billion times since.
- 1964 — Using suborbital rockets carrying Geiger counters new sources of cosmic X-rays are discovered. One of these X-ray sources, Cygnus X-1, confirmed in 1971 with the UHURU orbiting X-ray observatory, is soon accepted as the first plausible stellar-mass BH candidate (see, e.g., [110]). The UHURU orbiting X-ray observatory makes the first surveys of the X-ray sky discovering over 300 X-ray “stars”.
- 1965 — Penrose and Hawking prove that collapse of ordinary matter leads, under generic conditions, to spacetime singularities (the so-called “singularity theorems”) [608, 401]. A few years later, Penrose conjectures that these singularities, where quantum gravitational effects become important, are generically contained within BHs — The *cosmic censorship conjecture* [610, 767].
- 1965 — Weber builds the first GW detector, a resonant alluminium cylinder [771, 772].
- 1966 — May and White perform a full nonlinear numerical collapse simulation for some realistic equations of state [543].
- 1967 — Wheeler [661, 778] coins the term *black hole* (see the April 2009 issue of *Physics Today*, and Ref. [779] for a fascinating, first-person historical account).
- 1967, 1971 — Israel, Carter and Hawking prove that any stationary, vacuum BH is described by the Kerr solution [453, 188, 403, 406]. This result motivates Wheeler’s statement that “a BH has no hair” [661].
- 1968 — Veneziano proposes his dual resonance model, which will later be understood to be equivalent to an oscillating string [759]. This date is considered the dawn of SMT.
- 1969 — Penrose shows that the existence of an ergoregion allows to extract energy and angular momentum from a Kerr BH [610]. The wave analogue of the Penrose process is subsequently shown to occur by Zeldovich, who proves that dissipative rotating bodies (such as Kerr BHs, for which the dissipation is provided by the horizon) amplify incident waves in a process now called superradiance [827, 828].
- 1970 — Zerilli [829, 830] extends the Regge-Wheeler analysis to general perturbations of a Schwarzschild BH. He shows that the problem can be reduced to the study of a pair of Schröodingerlike equations, and applies the formalism to the problem of gravitational radiation emitted by infalling test particles.
- 1970 — Vishveshwara [762] studies numerically the scattering of GWs by BHs: at late times the waveform consists of damped sinusoids (now called ringdown waves, or quasi-normal modes).
- 1971 — Davis et al. [250] carry out the first quantitative calculation of gravitational radiation emission within BH perturbation theory, considering a particle falling radially into a Schwarzschild BH. Quasi-normal mode (QNM) ringing is excited when the particle crosses the maximum of the potential barrier of the Zerilli equation, located close to the unstable circular orbit for photons.
- 1973 — Bardeen, Carter and Hawking derive the four laws of BH mechanics [74].
- 1973 — Teukolsky [743] decouples and separates the equations for perturbations in the Kerr geometry using the Newman-Penrose formalism [575].
- 1973 — York [808, 809] introduces a split of the extrinsic curvature leading to the *Lichnerowicz-York conformai decomposition*, which underlies most of the initial data calculations in NR.
- 1973 — Thorne provides a criterium for BH formation, the *hoop conjecture* [750]; it predicts collapse to BHs in a variety of situations including very high-energy particle collisions, which were to become important in TeV-scale gravity scenarios.
- 1974 — Hulse and Taylor find the first pulsar, i.e., a radiating neutron star (NS), in a binary star system [447]. The continued study of this system over time has produced the first solid observational evidence, albeit indirect, for GWs. This, in turn, has further motivated the study of dynamical compact binaries and thus the development of NR and resulted in the 1993 Nobel Prize for Hulse and Taylor.
- 1975 — Using quantum field theory in curved space, Hawking finds that BHs have a thermal emission [405]. This result is one of the most important links between GR and quantum mechanics.
- 1977 — NR is born with coordinated efforts to evolve BH spacetimes [708, 287, 711].
- 1978 — Cunningham, Price and Moncrief [229, 230, 231] study radiation from relativistic stars collapsing to BHs using perturbative methods. QNM ringing is excited.
- 1979 — York [810] reformulates the canonical decomposition by ADM, casting the Einstein equations in a form now commonly (and somewhat misleadingly) referred to as the ADM equations.
- 1980 — Bowen & York develop the *conformai imaging* approach resulting in analytic solutions to the momentum constraints under the assumption of maximal slicing as well as conformal and asymptotic flatness [121].
- 1983 — Chandrasekhar’s monograph [195] summarizes the state of the art in BH perturbation theory, elucidating connections between different formalisms.
- 1985 — Stark and Piran [724] extract GWs from a simulation of rotating collapse to a BH in NR.
- 1985 — Leaver [504, 505, 506] provides the most accurate method to date to compute BH QNMs using continued fraction representations of the relevant wavefunctions.
- 1986 — McClintock and Remillard [547] show that the X-ray nova A0620-00 contains a compact object of mass almost certainly larger than 3 *M*_*⊙*_, paving the way for the identification of many more stellar-mass BH candidates.
- 1986 — Myers and Perry construct higher-dimensional rotating, topologically spherical, BH solutions [565].
- 1987 —’ t Hooft [736] argues that the scattering process of two point-like particles above the fundamental Planck scale is well described and calculable using classical gravity. This idea is behind the application of GR for modeling trans-Planckian particle collisions.
- 1989 — Echeverria [290] estimates the accuracy with which one can estimate the mass and angular momentum of a BH from QNM observations. The formalism is substantially refined in Refs. [97, 95].
- 1992 — The LIGO detector project is funded by the National Science Foundation. It reaches design sensitivity in 2005 [6]. A few years later, in 2009, the Virgo detector also reaches its design sensitivity [10].
- 1992 — Bona and Massó show that harmonic slicing has a singularity-avoidance property, setting the stage for the development of the “l+log” slicing [115].
- 1992 — D’Eath and Payne [256, 257, 258, 259] develop a perturbative method to compute the gravitational radiation emitted in the head-on collision of two BHs at the speed of light. Their second order result will be in good agreement with later numerical simulations of high-energy collisions.
- 1993 — Christodoulou and Klainerman show that Minkowski spacetime is nonlinearly stable [219].
- 1993 — Anninos et al. [37] first succeed in simulating the head-on collision of two BHs, and observe QNM ringing of the final BH.
- 1993 — Gregory and Laflamme show that black strings, one of the simplest higher-dimensional solutions with horizons, are unstable against axisymmetric perturbations [367]. The instability is similar to the Rayleigh-Plateau instability seen in fluids [167, 162]; the end-state was unclear.
- 1993 — Choptuik finds evidence of universality and scaling in gravitational collapse of a massless scalar field. “Small” initial data scatter, while “large” initial data collapse to BHs [212]; first use of mesh refinement in NR.
- 1994 — The “Binary Black Hole Grand Challenge Project”, the first large collaboration with the aim of solving a specific NR problem (modeling a binary BH coalescence), is launched [542, 213].
- 1995, 1998 — Through a conformal decomposition, split of the extrinsic curvature and use of additional variables, Baumgarte, Shapiro, Shibata and Nakamura [695, 78] recast the ADM equations as the so-called BSSN system, partly building on earlier work by Nakamura, Oohara and Kojima [569].
- 1996 — Brü gmann [140] uses mesh refinement for simulations of BH spacetimes in 3 + 1 dimensions.
- 1997 — Cactus 1.0 is released in April 1997. Cactus [154] is a freely available environment for collaboratively developing parallel, scalable, high-performance multidimensional component-based simulations. Many NR codes are based on this framework. Recently, Cactus also became available in the form of the Einstein Toolkit [521, 300].
- 1997 — Brandt & Brügmann [126] present *puncture* initial data as a generalization of Brill-Lindquist data to the case of generic Bowen-York extrinsic curvature.
- 1997 — Maldacena [536] formulates the AdS/CFT duality conjecture. Shortly afterward, the papers by Gubser, Klebanov, Polyakov [372] and Witten [798] establish a concrete quantitative recipe for the duality. The AdS/CFT era begins. In the same year, the correspondence is generalized to non-conformal theories in a variety of approaches (see [15] for a review). The terms “gauge/string duality”, “gauge/gravity duality” and “holography” appear (the latter had been previously introduced in the context of quantum gravity [737, 734]), referring to these generalized settings.
- 1998 — The hierarchy problem in physics — the huge discrepancy between the electroweak and the Planck scale — is addressed in the so-called *braneworld scenarios*, in which we live on a four-dimensional subspace of a higher-dimensional spacetime, and the Planck scale can be lowered to the TeV [46, 40, 638, 639].
- 1998 — First stable simulations of a single BH spacetime in fully *D* = 4 dimensional NR within a “characteristic formulation” [508, 362], and two years later within a Cauchy formulation [23].
- 1998 — The possibility of BH formation in braneworld scenarios is first discussed [45, 69]. Later work suggests BH formation could occur at the LHC [279, 353] or in ultra-high energy cosmic ray collisions [315, 33, 304].
- 1999 — Friedrich & Nagy [335] present the first well-posed formulation of the initial-boundary-value problem (IBVP) for the Einstein equations.
- 2000 — Brandt et al. [127] simulate the first grazing collisions of BHs using a revised version of the Grand Challenge Alliance code [227].
- 2000 — Shibata and Uryū [698] perform the first general relativistic simulation of the merger of two NSs. More recent simulations [62], using a technique developed by Baiotti and Rezzolla that circumvents singularity excision [64], confirm that ringdown is excited when the merger leads to BH formation. In 2006, Shibata and Uryū perform NR simulations of BH-NS binaries [699].
- 2001 — Emparan and Reall provide the first example of a stationary asymptotically flat vacuum solution with an event horizon of non-spherical topology — the “black ring” [307].
- 2001 — Horowitz and Maeda suggest that black strings do not fragment and that the end-state of the Gregory-Laflamme instability may be an inhomogeneous string [440], driving the development of the field. Non-uniform strings are constructed perturbatively by Gubser [371] and numerically by Wiseman, who, however, shows that these cannot be the end-state of the Gregory-Laflamme instability [789].
- 2003 — In a series of papers [479, 452, 480], Kodama and Ishibashi extend the Regge-Wheeler-Zerilli formalism to higher dimensions.
- 2003 — Schnetter et al. [684] present the publically available Carpet mesh refinement package, which has constantly been updated since and is being used by many NR groups.
- 2005 — Pretorius [629] achieves the first long-term stable numerical evolution of a BH binary. Soon afterwards, other groups independently succeed in evolving merging BH binaries using different techniques [159, 65]. The waveforms indicate that ringdown contributes a substantial amount to the radiated energy.
- 2007 — First results from NR simulations show that spinning BH binaries can coalesce to produce BHs with very large recoil velocities [363, 161].
- 2007 — Boyle et al. [122] achieve unprecedented accuracy and number of orbits in simulating a BH binary through inspiral and merger with a spectral code that later becomes known as “SpEC” and uses multi-domain decomposition [618] and a dual coordinate frame [678].
- 2008 — The first simulations of high-energy collisions of two BHs are performed [719]. These were later generalized to include spin and finite impact parameter collisions, yielding zoom-whirl behavior and the largest known luminosities [697, 720, 717, 716].
- 2008 — First NR simulations in AdS for studying the isotropization of a strongly coupled ${\mathcal N} = 4$ supersymmetric Yang-Mills plasma through the gauge/gravity duality [205].
- 2009 — Dias et al. show that rapidly spinning Myers-Perry BHs present zero-modes, signalling linear instability against axially symmetric perturbations [272], as previously argued by Emparan and Myers [305]. Linearly unstable modes were subsequently explored in Refs. [271, 270].
- 2009 — Shibata and Yoshino evolve Myers-Perry BHs nonlinearly and show that a non-axisymmetric instability is present [701].
- 2009 — Collisions of boson stars show that at large enough energies a BH forms, in agreement with the hoop conjecture [216]. Subsequent investigations extend these results to fluid stars [288, 647].
- 2010 — Building on previous work [215], Lehner and Pretorius study the nonlinear development of the Gregory-Laflamme instability in five dimensions, which shows hints of pinch-off and cosmic censorship violation [511].
- 2010, 2011 — First nonlinear simulations describing collisions of higher-dimensional BHs, by Zilhao et al., Witek et al. and Okawa et al. [841, 797, 587].
- 2011 — Bizoń and Rostworowski extend Choptuik’s collapse simulations to asymptotically AdS spacetimes [108], finding evidence that generic initial data collapse to BHs, thereby conjecturing a nonlinear instability of AdS.
- 2013 — Collisions of spinning BHs provide evidence that multipolar structure of colliding objects is not important at very large energies [716].

## Strong Need for Strong Gravity

The need for NR is almost as old as GR itself, but the real push to develop these tools came primarily from the necessity to understand conceptual issues such as the end-state of collapse and the two-body problem in GR as well as from astrophysics and GW astronomy. The breakthroughs in the last years have prompted a serious reflexion and examination of the multitude of problems and fields that stand to gain from NR tools and results, if extended to encompass general spacetimes. The following is a brief description of each of these topics. The range of fundamental issues for which accurate strong-gravity simulations are required will hopefully become clear.

### Astrophysics

#### Gravitational wave astronomy

GWs are one of the most fascinating predictions of GR. First conceived by Einstein [294, 296], it was unclear for a long time whether they were truly physical. Only in the 1960s were their existence and properties founded on a sound mathematical basis (see [450, 451] and references therein). In the same period, after the seminal work of Weber [770], the scientific community was starting a growing experimental effort to directly detect GWs. The first detectors were resonant antennas; their sensitivity was far too low to detect any signal (unless a nearby galactic supernova exploded when the detector was taking data), and they were eventually replaced by interferometric detectors. The first generation of such detectors (LIGO, Virgo, GEO600, TAMA) did not reveal any gravitational signal, but the second generation (Advanced LIGO/Virgo [517, 761]) should be operative by 2015 and is expected to make the first detection of GWs. In parallel, Pulsar Timing Arrays are promising to detect ultra-low frequency GWs [507], whereas the polarization of the cosmic microwave background can be used as a detector of GWs from an inflationary epoch in the very early universe [690, 369, 725, 659, 7]. In the subsequent years more sensitive detectors, such as the underground cryogenic interferometer KAGRA [462] (and, possibly, ET [299]) and possibly a space-based detector such as LISA/eLISA [302], will allow us to know the features of the signal in more detail, and then to use this information to learn about the physics of the emitting sources, and the nature of the gravitational interaction.

Soon after the beginning of the experimental efforts to build a GW detector, it became clear that the detection of GWs emitted by astrophysical sources would open a new window of observational astronomy, in addition to the electromagnetic spectrum, neutrinos, cosmic rays, etc. The impact of such a detection would be similar to that of X-rays from astrophysical sources, i.e., the birth of a new branch of astronomy: “GW astronomy” [628, 370, 686]. In this new field, source modelling is crucial, since a theoretical understanding of the expected GW sources is needed to enhance the chances of detection and to extract the relevant physics. Indeed, template-matching techniques — frequently used in data analysis — can be helpful to extract the signal from the detector noise, but they require an a-priori knowledge of the waveforms [752].

A wide scientific community formed, with the aim to model the physical processes that are expected to produce a detectable GW signal, and to compute the emitted gravitational waveform (which depends on the unknown parameters of the source and of the emitting process). Together with the understanding of the two-body problem in GR, this effort was one of the main driving forces leading to the development of NR. Indeed, many promising GW sources can only be modeled by solving the fully nonlinear Einstein equations numerically.

Ground-based interferometers are (and are expected to be in the next decades) sensitive to signals with frequencies ranging from some tens of Hz to about one kHz. Space-based interferometers would be sensitive at much lower frequencies: from some mHz to about one tenth of Hz. GW astronomy, of course, is presently concerned with sources emitting GWs in these frequency bands.

Many astrophysical processes are potential sources for GW detectors. In the following, we shall briefly discuss only some of them, i.e., those that require NR simulations to be modeled: compact binary inspirals, and instabilities of rotating NSs. We shall not discuss supernova core collapse — one of the first GW sources that have been studied with NR, and one of the most problematic to model — since it will be discussed in Section 3.1.2.

Compact binary inspirals, i.e., the inspiral and merger of binary systems formed by BHs and/or NSs, are the most promising GW sources to be detected. Advanced LIGO/Virgo are expected to detect some tens of these sources per year [5]. While the inspiral phase of a compact binary system can be accurately modeled through PN approaches, and the final (”ringdown”) phase, when the BH resulting from the coalescence oscillates in its characteristic proper modes, can be accurately described through perturbative approaches, the intermediate merger phase can only be modeled by NR. This task has posed formidable theoretical and computational challenges to the scientific community.

The numerical simulation of the merger phase of a BH-BH binary coalescence, and the determination of the emitted gravitational waveform, had been an open problem for decades, until it was solved in 2005 [629, 159, 65]. This challenge forced the gravitational community to reflect on deep issues and problems arising within Einstein’s theory, such as the role of singularities and horizons, and the possible ways to locally define energy and momentum.

BH-NS and NS-NS binary coalescences pose a different sort of problems than those posed by BH-BH coalescences. They are not a “clean” system such as purely vacuum BH spacetimes, characterized by the gravitational interaction only. An accurate numerical modeling involves various branches of physics (nuclear physics, neutrinos, electromagnetic fields), and requires the understanding of many different processes. Typically, NR simulations of BH-NS and NS-NS mergers make simplifying assumptions, both because taking into account all aspects at the same time would be too complicated, and because some of them are not fully understood. Currently, the behaviour of matter in the inner core of a NS is one of the challenges to be tackled. Indeed, nuclear physicists still do not understand which is the equation of state of matter at such extreme conditions of density and temperature (see, e.g., [501] and references therein). This uncertainty reflects our ignorance on the behaviour of the hadronic interactions in the non-perturbative regime. On the other hand, understanding the NS equation of state is considered one of the main outcomes expected from the detection of a GW signal emitted by NSs, for instance in compact binary coalescences [583, 640, 80, 738].

Neutron star oscillations are also a candidate GW source for ground-based interferometers. When perturbed by an external or internal event, a NS can be set into non-radial damped oscillations, which are associated to the emission of GWs. The characteristic frequencies of oscillation, the QNMs, are characterized by their complex frequency *ω* = *σ* + *i*/*τ*, where *σ* is the pulsation frequency, and *σ* is the damping time of the oscillation (for detailed discussions on the QNMs of NSs and BHs see [487, 580, 316, 95] and references therein).

If a NS rotates, its oscillations can become unstable. In this case, the oscillation grows until the instability is suppressed by some damping mechanism or by nonlinear effects; this process can be associated to a large GW emission (see, e.g., [34] and references therein). These instabilities may explain the observed values of the NS rotation rates [101]. Their numerical modeling, however, is not an easy task. Perturbative approaches, which easily allow one to compute the QNMs of non-rotating NSs, become very involved in the presence of rotation. Therefore, the perturbation equations can only be solved with simplifying assumptions, which make the model less accurate. Presently, NR is the only way to model stationary, rapidly rotating NSs (see, e.g., [728] and references therein), and it has recently been applied to model their oscillations [842].

#### Collapse in general relativity

Decades before any observation of supermassive compact objects, and long before BHs were understood, Chandrasekhar showed that the electron degeneracy pressure in very massive white dwarfs is not enough to prevent them from imploding [193]. Similar conclusions were reached later by Oppenheimer and Volkoff, for neutron degeneracy pressure in NSs [590]. We can use Landau’s original argument to understand these results [498, 499, 691]: consider a star of radius *R* composed of *N* fermions, each of mass *m*_*F*_. The momentum of each fermion is *p*_*F*_ ∼ *ħn*^1/3^, with *n* = *N*/*R*^3^ the number density of fermions. In the relativistic regime, the Fermi energy per particle then reads *E*_*F*_ = *p*_*F*_*c* = *ħcN*^1/3^/*R*. The gravitational energy per fermion is approximately ${E_G} \sim - Gm_F^2/R$, and the star’s total energy is thus,

2 $$E \equiv {E_F} + {E_G} = {{\hbar c{N^{1/3}} - GNm_F^2} \over R}.$$

For small *N*, the total energy is positive, and we can decrease it by increasing *R*. At some point the fermion becomes non-relativistic and ${E_F} \sim p_F^2 \sim 1/{R^2}$. In this regime, the gravitational binding energy *E*_*G*_ dominates over *E*_*F*_, the total energy is negative and tends to zero as *R* → *∞*. Thus there is a local minimum and the star is stable. However, for large *N* in the relativistic regime the total energy is negative, and can be made even more negative by *decreasing R*: it is energetically favoured for the star to continually collapse! The threshold for stability occurs at a zero of the total energy, when

3 $${N_{\max}} > {\left({{{\hbar c} \over {Gm_F^2}}} \right)^{3/2}},$$

4 $${M_{\max}} \sim {N_{\max}}{m_F} \sim {\left({{{\hbar c} \over {Gm_F^{4/3}}}} \right)^{3/2}}.$$

for neutrons, stars with masses above ∼ 3 *M*_*⊙*_ cannot attain equilibrium.

What is the fate of massive stars whose pressure cannot counter-balance gravity? Does the star’s material continually collapse to a single point, or is it possible that pressure or angular momentum become so important that the material bounces back? The answer to these questions would take several decades more, and was one of the main driving forces to develop solid numerical schemes to handle Einstein’s equations.

Other developments highlighted the importance of understanding gravitational collapse in GR. One was the advent of GW detectors. The strongest sources of GWs are compact and moving relativistically, and supernovae are seemingly ideal: they occur frequently and are extremely violent. Unfortunately, Birkhoff’s theorem implies that spherically symmetric sources do not radiate. Thus a careful, and much more complex analysis of collapse is required to understand these sources.

In parallel, BH physics was blooming. In the 1970s one key result was established: the uniqueness theorem, stating that — under general regularity assumptions — the only stationary, asymptotically flat, vacuum solution of Einstein’s field equations is the Kerr BH. Thus, *if* a horizon forms, the final stationary configuration is expected to be of the Kerr family. This important corollary of Einstein’s field equations calls for a dynamical picture of BH formation through collapse and an understanding of how the spacetime multipolar structure dynamically changes to adapt to the final Kerr solution as a BH forms.

#### Kicks

It has been known since the early 1960s that GWs emitted by accelerated particles do not only carry energy but also momentum away from the system on which thus is imparted a *kick* or *recoil*. This effect was first studied by Bonnor & Rotenberg [119] for the case of a system of oscillating particles, and has been identified by Peres [612] to be at leading order due to the interference of the mass quadrupole radiation with the mass octupole or flow quadrupole.

From an astrophysical point of view, the most important processes generating such gravitational recoil are the collapse of a stellar core to a compact object and the inspiral and merger of compact binaries. Supermassive BHs with masses in the range of 10^5^ *M*_*⊙*_ to 10^10^ *M*_*⊙*_ in particular are known to reside at the centre of many galaxies and are likely to form inspiralling binary systems as a consequence of galaxy mergers. Depending on the magnitude of the resulting velocities, kicks can in principle displace or eject BHs from their hosts and therefore play an important role in the formation history of these supermassive BHs.

The first calculations of recoil velocities based on perturbative techniques have been applied to gravitational collapse scenarios by Bekenstein [84] and Moncrief [556]. The first analysis of GW momentum flux generated by binary systems was performed by Fitchett [322] in 1983 for two masses in Keplerian orbit. The following two decades saw various (semi-)analytic calculations for inspiraling compact binary systems using the particle approximation, post-Newtonian techniques and the close-limit approach (see Section 5 for a description of these techniques and main results). In conclusion of these studies, it appeared likely that the gravitational recoil from non-spinning binaries was unlikely to exceed a few hundred km/s. Precise estimates, however, are dependent on an accurate modeling of the highly nonlinear late inspiral and merger phase and therefore required NR simulations. Furthermore, the impact of spins on the resulting velocities remained essentially uncharted territory until the 2005 breakthroughs of NR made possible the numerical simulations of these systems. As it turned out, some of the most surprising and astrophysically influencial results obtained from NR concern precisely the question of the gravitational recoil of spinning BH binaries.

#### Astrophysics beyond Einstein gravity

Although GR is widely accepted as the standard theory of gravity and has survived all experimental and observational (weak field) scrutiny, there is convincing evidence that it is not the ultimate theory of gravity: since GR is incompatible with quantum field theory, it should be considered as the low energy limit of some, still elusive, more fundamental theory. In addition, GR itself breaks down at small length scales, since it predicts singularities. For large scales, on the other hand, cosmological observations show that our universe is filled with dark matter and dark energy, of as yet unknown nature.

This suggests that the strong-field regime of gravity — which has barely been tested so far — could be described by some modification or extension of GR. In the next few years both GW detectors [786, 826] and astrophysical observations [635] will provide an unprecedented opportunity to probe the strong-field regime of the gravitational interaction, characterized by large values of the gravitational field $\sim {{GM} \over {r{c^2}}}$ or of the spacetime curvature $\sim {{GM} \over {{r^3}{c^2}}}$ (it is a matter of debate which of the two parameters is the most appropriate for characterizing the strong-field gravity regime [635, 826]). However, our present theoretical knowledge of strong-field astrophysical processes is based, in most cases, on the a-priori assumption that GR *is* the correct theory of gravity. This sort of *theoretical bias* [825] would strongly limit our possibility of testing GR.

It is then of utmost importance to understand the behaviour of astrophysical processes in the strong gravity regime beyond the assumption that GR is the correct theory of gravity. The most powerful tool for this purpose is probably NR; indeed, although NR has been developed to solve Einstein’s equations (possibly coupled to other field equations), it can in principle be extended and modified, to model physical processes in alternative theories of gravity. In summary, NR can be applied to specific, well motivated theories of gravity. These theories should derive from — or at least be inspired by — some more fundamental theories or frameworks, such as for instance SMT [366, 624] (and, to some extent, Loop Quantum Gravity [657]). In addition, such theories should allow a well-posed initial-value formulation of the field equations. Various arguments suggest that the modifications to GR could involve [826] (i) additional degrees of freedom (scalar fields, vector fields); (ii) corrections to the action at higher order in the spacetime curvature; (iii) additional dimensions.

Scalar-tensor theories for example (see, e.g., [337, 783] and references therein), are the most natural and simple generalizations of GR including additional degrees of freedom. In these theories, which include for instance Brans-Dicke gravity [128], the metric tensor is non-minimally coupled with one or more scalar fields. In the case of a single scalar field (which can be generalized to multi-scalar-tensor theories [242]), the action can be written as

5 $$S = {1 \over {16\pi G}}\int {{{\rm{d}}^4}x} \sqrt {- g} [F(\phi)R - 8\pi Gz(\phi){g^{\mu \nu}}{\partial _\mu}\phi {\partial _\nu}\phi - U(\phi)] + {S_m}({\psi _m},{g_{\mu \nu}})$$

where *R* is the Ricci scalar associated to the metric *g*_*μν*_, *F, Z, U* are arbitrary functions of the scalar field *ϕ*, and *S*_*m*_ is the action describing the dynamics of the other fields (which we call “matter fields”, *ψm*). A more general formulation of scalar-tensor theories yielding second order equations of motion has been proposed by Horndeski [435] (see also Ref. [260]).

Scalar-tensor theories can be obtained as low-energy limits of SMT [342]; this provides motivation for studying these theories on the grounds of fundamental physics. An additional motivation comes from the recently proposed “axiverse” scenario [49, 50], in which ultra-light axion fields (pseudo-scalar fields, behaving under many respects as scalar fields) arise from the dimensional reduction of SMT, and play a role in cosmological models.

Scalar-tensor theories are also appealing alternatives to GR because they predict new phenomena, which are not allowed in GR. In these theories, the GW emission in compact binary coalescences has a dipolar (*ℓ* =1) component, which is absent in GR; if the scalar field has a (even if extremely small) mass, superradiant instabilities occur [183, 604, 794], which can determine the formation of floating orbits in extreme mass ratio inspirals [165, 824], and these orbits affect the emitted GW signal; last but not least, under certain conditions isolated NSs can undergo a phase transition, acquiring a nontrivial scalar-field profile (*spontaneous scalarization* [242, 243]) while dynamically evolving NSs — requiring full NR simulations to understand — may display a similar effect (*dynamical scalarization* [73, 596]). A detection of one of these phenomena would be a smoking gun of scalar-tensor gravity.

These theories, whose well-posedness has been proved [669, 670], are a perfect arena for NR. Recovering some of the above smoking-gun effects is extremely challenging, as the required timescales are typically very large when compared to any other timescales in the problem.

Other examples for which NR can be instrumental include theories in which the EinsteinHilbert action is modified by including terms quadratic in the curvature (such as *R*^2^, *R*_*μν*_*R*^*μν*^, ${R_{\mu \nu \alpha \beta}}{R^{\mu \nu \alpha \beta}},{\epsilon _{\mu \nu \alpha \beta}}{R^{\mu \nu \rho \sigma}}{R^{\alpha \beta}}_{\rho \sigma}$), possibly coupled with scalar fields, or theories which explicitly break Lorentz invariance. In particular, Einstein-Dilaton-Gauss-Bonnet gravity and Dynamical Chern-Simons gravity [602, 27] can arise from SMT compactifications, and Dynamical Chern-Simons gravity also arises in Loop Quantum Gravity; theories such as Einstein-Aether [456] and “Horava-Lifshitz” gravity [433], which break Lorentz invariance (while improving, for instance, renormalizability properties of GR), allow the basic tenets of GR to be challenged and studied in depth.

### Fundamental and mathematical issues

#### Cosmic censorship

Spacetime singularities signal the breakdown of the geometric description of the spacetime, and can be diagnosed by either the blow-up of observer-invariant quantities or by the impossibility to continue timelike or null geodesics past the singular point. For example, the Schwarzschild geometry has a curvature invariant *R*^*abcd*^*R*_*abcd*_ = 48 *G*^2^*M*^2^/(c^4^r^6^) in Schwarzschild coordinates, which diverges at *r* = 0, where tidal forces are also infinite. Every timelike or null curve crossing the horizon necessarily hits the origin in finite proper time or affine parameter and, therefore, the theory breaks down at these points: it fails to predict the future development of an object that reaches the singular point. Thus, the classical theory of GR, from which spacetimes with singularities are obtained, is unable to describe these singular points and contains its own demise. Adding to this classical breakdown, it is likely that quantum effects take over in regions where the curvature radius becomes comparable to the scale of quantum processes, much in the same way as quantum electrodynamics is necessary in regions where EM fields are large enough (as characterized by the invariant *E*^2^ − *B*^2^) that pair creation occurs. Thus, a quantum theory of gravity might be needed close to singularities.

It seems therefore like a happy coincidence that the Schwarzschild singularity is cloaked by an event horizon, which effectively causally disconnects the region close to the singularity from outside observers. This coincidence introduces a miraculous cure to GR’s apparently fatal disease: one can continue using classical GR for all practical purposes, while being blissfully ignorant of the presumably complete theory that smoothens the singularity, as all those extra-GR effects do not disturb processes taking place outside the horizon.

Unfortunately, singularities are expected to be quite generic: in a remarkable set of works, Hawking and Penrose have proved that, under generic conditions and symmetries, collapse leads to singularities [608, 402, 408, 570]. Does this always occur, i.e., are such singularities *always* hidden to outside observers by event horizons? This is the content of Penrose’s “cosmic censorship conjecture”, one of the outstanding unsolved questions in gravity. Loosely speaking, the conjecture states that physically reasonable matter under generic initial conditions only forms singularities hidden behind horizons [767].

The cosmic-censorship conjecture and the possible existence of naked singularities in our universe has triggered interest in complex problems which can only be addressed by NR. This is a very active line of research, with problems ranging from the collapse of matter to the nonlinear stability of “black” objects.

#### Stability of black hole interiors

As discussed in Section 3.1.2, the known fermionic degeneracies are unable to prevent the gravitational collapse of a sufficiently massive object. Thus, if no other (presently unkown) physical effect can prevent it, according to GR, a BH forms. From the uniqueness theorems (cf. Section 4.1.1), this BH is described by the Kerr metric. Outside the event horizon, the Kerr family — a 2-parameter family described by mass *M* and angular momentum *J* — varies smoothly with its parameters. But inside the event horizon a puzzling feature occurs. The interior of the *J* = 0 solution — the Schwarzschild geometry — is qualitatively different from the *J* = 0 case. Indeed, inside the Schwarzschild event horizon a point-like, spacelike singularity creates a boundary for spacetime. Inside the 0 < *J* < *M*^2^ Kerr event horizon, by contrast, there is a ring-like, timelike singularity, beyond which another asymptotically flat spacetime region, with *r* < 0 in Boyer-Lindquist coordinates, may be reached by causal trajectories. The puzzling feature is then the following: according to these exact solutions, the interior of a Schwarzschild BH, when it absorbes an infinitesimal particle with angular momentum, must drastically change, in particular by creating another asymptotically flat region of spacetime.

This latter conclusion is quite unreasonable. It is more reasonable to expect that the internal structure of an eternal Kerr BH must be very different from that of a Kerr BH originating from gravitational collapse. Indeed, there are arguments, of both physical [609] and mathematical nature [198], indicating that the Cauchy horizon (i.e., inner horizon) of the eternal charged or rotating hole is unstable against small (linear) perturbations, and therefore against the accretion of any material. The natural question is then, what is the endpoint of the instability?

As a toy model for the more challenging Kerr case, the aforementioned question was considered in the context of spherical perturbations of the RN BH by Poisson and Israel. In their seminal work, the phenomenon of *mass inflation* was unveiled [621, 622]: if ingoing and outgoing streams of matter are simultaneously present near the inner horizon, then relativistic counter-streaming^2^ between those streams leads to exponential growth of gauge-invariant quantities such as the interior (Misner-Sharp [552]) mass, the center-of-mass energy density, or curvature scalar invariants. Since this effect is causally disconnected from any external observers, the mass of the BH measured by an outside observer remains unchanged by the mass inflation going on in the interior. But this inflation phenomenon causes the spacetime curvature to grow to Planckian values in the neighbourhood of the Cauchy horizon. The precise nature of this evolution for the Kerr case is still under study. For the simpler RN case, it has been argued by Dafermos, using analytical methods, that the singularity that forms is not of space-like nature [234]. Fully nonlinear numerical simulations will certainly be important for understanding this process.

#### Most luminous events in the universe

The most advanced laser units on the planet can output luminosities as high as ∼ 10^18^ W [301], while at ∼ 10^26^ W the Tsar Bomba remains the most powerful artificial explosion ever [732]. These numbers pale in comparison with strongly dynamical astrophysical events: a *γ*-ray burst, for instance, reaches luminosities of approximately ∼ 10^45^ W. A simple order of magnitude estimate can be done to estimate the total luminosity of the universe in the EM spectrum, by counting the total number of stars, roughly 10^23^ [443]. If all of them have a luminosity equal to our Sun, we get a total luminosity of approximately ∼ 10^49^ W, a number which can also be arrived at through more careful considerations [781]. Can one possibly surpass this astronomical number?

In four spacetime dimensions, there is only one constant with dimensions of energy per second that can be built out of the classical universal constants. This is the Planck luminosity ${\mathcal L_G}$,

6 $${\mathcal L}_G \equiv {{{c^5}} \over G} = 3.7 \times {10^{52}}{\rm{W}}.$$

The quantity ${\mathcal L_G}$ should control gravity-dominated dynamical processes; as such it is no wonder that these events release huge luminosities. Take the gravitational collapse of a compact star with mass *M* and radius *R* ∼ *GM*/*c*^2^. During a collapse time of the order of the infall time, $\tau \sim R/\sqrt {GM/R} \sim GM/{c^3}$, the star can release an energy of up to *Mc*^2^. The process can therefore yield a power as large as ${c^5}/G = {\mathcal L_G}$. It was conjectured by Thorne [751] that the Planck luminosity is in fact an upper limit for the luminosity of *any* process in the universe.^3^ The conjecture was put on a somewhat firmer footing by Gibbons who has shown that there is an upper limit to the *tension* of *c*^4^/(4G), implying a limit in the luminosity of ${{\mathcal L}_G}/4$ [349].

*Are* such luminosities ever attained in practice, is there any process that can reach the Planck luminosity and outshine the entire universe? The answer to this issue requires once again a peek at gravity in strongly dynamical collisions with full control of strong-field regions. It turns out that high energy collisions of BHs do come close to saturating the bound (6) and that in general colliding BH binaries are more luminous than the entire universe in the EM spectrum [719, 720, 717, 716].

#### Higher dimensions

Higher-dimensional spacetimes are a natural framework for mathematicians and have been of general interest in physics, most notably as a tool to unify gravity with the other fundamental interactions. The quest for a unified theory of all known fundamental interactions is old, and seems hopeless in four-dimensional arenas. In a daring proposal however, Kaluza and Klein, already in 1921 and 1926 showed that such a programme might be attainable if one is willing to accept higher-dimensional theories as part of the fundamental picture [463, 476] (for a historical view, see [283]).

Consider first for simplicity the *D*-dimensional Klein-Gordon equation □*ϕ*(*x*^*μ*^,*z*^*i*^) = 0 (*ϕ* = 0,…,3, *i* = 4,…, *D* − 1), where the (*D* − 4) extra dimensions are compact of size *L*. Fourier decompose in ${z^j},\;\;{\rm{i}}{\rm{.e}},\;\;\phi ({x^\mu},{z^j}) = \sum\nolimits_n {\psi ({x^\mu})} {e^{in{z^j}/L}}$, to get $\square \psi - {{{n^2}} \over {{L^2}}}\psi = 0$, where here □ is the four-dimensional d’Alembertian operator. As a consequence,

i) the fundamental, homogenous mode *n* = 0 is a massless *four-dimensional* field obeying the same Klein-Gordon equation, whereas
ii) even though we started with a higher-dimensional massless theory, we end up with a tower of massive modes described by the four-dimensional massive Klein-Gordon equation, with mass terms proportional to *n*/*L*. One important, generic conclusion is that the higher-dimensional (fundamental) theory imparts mass terms as imprints of the extra dimensions. As such, the effects of extra dimensions are in principle testable. However, for very small *L* these modes have a very high-energy and are very difficult to excite (to “see” an object of length *L* one needs wavelengths of the same order or smaller), thereby providing a plausible explanation for the non-observation of extra dimensions in everyday laboratory experiments.

The attempts by Kaluza and Klein to unify gravity and electromagnetism considered five-dimensional Einstein field equations with the metric appropriately decomposed as,

7 $${\rm{d}}{\hat s^2} = {e^{\alpha \phi}}{\rm{d}}{s^2} + {e^{- 2\alpha \phi}}{({\rm{d}}z + {\mathcal A})^2}.$$

Here, d*s*^2^ = *g*_*μν*_ d*x*^*μ*^ *dx*^*ν*^ is a four-dimensional geometry, $\mathcal A = {A_\mu}\;{\rm{d}}{x^\mu}$ is a gauge field and *ϕ* is a scalar; the constant *α* can be chosen to yield the four-dimensional theory in the Einstein frame. *Assuming* all the fields are independent of the extra dimension *z*, one finds a set of *four-dimensional* Einstein-Maxwell-scalar equations, thereby *almost* recovering both GR and EM [283]. This is the basic idea behind the Kaluza-Klein procedure, which unfortunately failed due to the presence of the (undetected) scalar field.

The idea of using higher dimensions was to be revived decades later in a more sophisticated model, eventually leading to SMT. The development of the gauge/gravity duality (see Section 3.3.1 below) and TeV-scale scenarios in high-energy physics (see Section 3.3.2) highlighted the importance of understanding Einstein’s equations in a generic number of dimensions. Eventually, the study of Einstein’s field equations in *D*-dimensional backgrounds branched off as a subject of its own, where *D* is viewed as just another parameter in the theory. This area has been extremely active and productive and provides very important information on the content of the field equations and the type of solutions it admits. Recently, GR in the large *D* limit has been suggested as a new tool to gain insight into the *D* dependence of physical processes [309].

The uniqueness theorems, for example, are known to break down in higher dimensions, at least in the sense that solutions are uniquely characterized by asymptotic charges. BHs of spherical topology — the extension of the Kerr solution to higher dimensions — can co-exist with black rings [307]. In fact, a zoo of black objects are known to exist in higher dimensions, but the dynamical behavior of this zoo (of interest to understand stability of the solutions and for collisions at very high energies) is poorly known, and requires NR methods to understand.

One other example requiring NR tools is the instability of black strings. Black strings are one of the simplest vacuum solutions one can construct, by extending trivially a four-dimensional Schwarzschild BH along an extra, flat direction. Such solutions are unstable against long wavelength perturbations in the fifth dimension, which act to fragment the string. This instability is known as the Gregory-Laflamme instability [367]. The instability is similar in many aspects to the Rayleigh-Plateau instability seen in fluids, which does fragment long fluid cylinders [167]. However, the same scenario in the black string case would seem to lead to cosmic censorship violation, since the pinch-off would be accompanied by (naked) regions of unbounded curvature.^4^ Evidence that the Gregory-Laflamme does lead to disruption of strings was recently put forward [511].

### High-energy physics

#### The gauge/gravity duality

The gauge/gravity duality, or AdS/CFT correspondence, is the conjecture, first proposed by Maldacena in 1998 [536], and further developed in [798, 372], that string theory on an AdS spacetime (times a compact space) is *dual* (i.e., equivalent under an appropriate mapping) to a CFT defined on the boundary of the AdS space. Since its proposal, this conjecture has been supported by impressive and compelling evidence, it has branched off to, e.g., the AdS/Condensed Matter correspondence [396], and it has inspired other proposals of duality relations with a similar spirit, such as the dS/CFT correspondence [731] and the Kerr/CFT correspondence [373]. All these dualities are examples of the *holographic principle*, which has been proposed in the context of quantum gravity [737, 734], stating that the information contained in a *D*-dimensional gravitational system is encoded in some boundary of the system. The paradigmatic example of this idea is a BH spacetime, whose entropy is proportional to the horizon area.

These dualities — in which strong gravity systems play a crucial role — offer tools to probe strongly coupled gauge theories (in *D* − 1 dimensions) by studying classical gravity (in *D* dimensions). For instance, the confinement/deconfinement phase transition in quantum chromodynamicslike theories has been identified with the Hawking-Page phase transition for AdS BHs [799]. Away from thermal equilibrium, the quasi-normal frequencies of AdS BHs have been identified with the poles of retarded correlators describing the relaxation back to equilibrium of a perturbed dual field theory [439, 104]. The strongly coupled regime of gauge theories is inaccessible to perturbation theory and therefore this new tool has created expectations for understanding properties of the plasma phase of non-Abelian quantum field theories at non-zero temperature, including the transport properties of the plasma and the propagation and relaxation of plasma perturbations, experimentally studied at the Relativistic Heavy Ion Collider and now also at the LHC [189]. Strong coupling can be tackled by lattice-regularized thermodynamical calculations of quantum chromo-dynamics, but the generalization of these methods beyond static observables to characterizing transport properties has limitations, due to computational costs. An example of an experimentally accessible transport property is the dimensionless ratio of the shear viscosity to the entropy density. Applying the gauge/gravity duality, this property can be computed by determining the absorption cross section of low-energy gravitons in the dual geometry (a BH/black brane) [490], obtaining a result compatible with the experimental data. This has offered the holographic description of heavy ion collisions phenomenological credibility. An outstanding theoretical challenge in the physics of heavy ion collisions is the understanding of the ‘early thermalization problem’: the mechanism driving the short — less than 1 fm/c [414] — time after which experimental data agrees with a hydrodynamic description of the quark-gluon plasma. Holography uses ${\mathcal N} = 4$ Super Yang-Mills theory as a learning ground for the real quark-gluon plasma. Then, the formation of such plasma in the collision of high-energy ions has been modeled, in its gravity dual, by colliding gravitational shock waves in five-dimensional AdS space [205]. These strong gravity computations have already offered some insight into the early thermalization problem, by analyzing the formation and settling down of an AdS BH in the collision process. But the use of shock waves is still a caricature of the process, which could be rendered more realistic, for instance, by colliding other highly boosted lumps of energy or BHs in AdS.

Another example of gauge/gravity duality is the AdS/Condensed Matter correspondence, between field theories that may describe superconductors and strong gravity [396, 437, 397]. The simplest gravity theory in this context is Einstein-Maxwell-charged scalar theory with negative cosmological constant. The RN-AdS BH solution of this theory, for which the scalar field vanishes, is unstable for temperatures *T* below a critical temperature *T*_*c*_. If triggered, the instability leads the scalar field to condense into a non-vanishing profile creating a scalar hair for the BH and breaking the *U* (1)-gauge symmetry spontaneously. The end point of the instability is a static solution that has been constructed numerically and has properties similar to those of a superconductor [398]. Thus, this instability of the RN-AdS BH at low temperature was identified with a superconducting phase transition, and the RN-AdS and hairy BHs in the gravitational theory, respectively, were identified with the normal and superconducting phases of a holographic superconductor, realized within the dual field theory. Holographic superconductors are a promising approach to understanding strongly correlated electron systems. In particular, non-equilibrium processes of strongly correlated systems, such as superconductors, are notoriously difficult and this holographic method offers a novel tool to tackle this longstanding problem. In the gauge/gravity approach, the technical problem is to solve the classical dynamics of strong gravitational systems in the dual five-dimensional spacetime. Using the AdS/CFT dictionary, one then extracts the dynamics of the phase transition for the boundary theory and obtains the time dependence of the superconducting order parameter and the relaxation time scale of the boundary theory.

#### Theories with lower fundamental Planck scale

As discussed in Section 3.2.4, higher-dimensional theories have been suggested since the early days of GR as a means to achieve unification of fundamental interactions. The extra dimensions have traditionally been envisaged as compact and very small (∼ Planck length), in order to be compatible with high energy experiments. Around the turn of the millennium, however, a new set of scenarios emerged wherein the extra dimensions are only probed by the gravitational interaction, because a confining mechanism ties the standard model interactions to a 3 + 1-dimensional subspace (which is called the “brane”, while the higher-dimensional spacetime is called the “bulk”). These models — also called “braneworld scenarios” — can be considered SMT inspired. The main ideas behind them are provided by SMT, including the existence of extra dimensions and also the existence of subspaces, namely *Dirichlet-branes*, on which a well defined mechanism exists to confine the standard model fields.

Our poor knowledge of the gravitational interaction at very short scales (below the millimeter at the time of these proposals, below ≲ 10^−4^ meters at the time of writing [802, 783]), allows large [40, 46, 279] or infinitely large extra dimensions [638, 639]. The former are often called *ADD models*, whereas the latter are known as *Randall-Sundrum scenarios*. Indeed these types of extra dimensions are compatible with high energy phenomenology. Besides being viable, these models (or at least some of them) have the conceptual appeal of providing an explanation for the “hierarchy problem” of particle physics: the large hierarchy between the electroweak scale (∼ 250 GeV) and the Planck scale (∼ 10^19^ GeV), or in other words, why the gravitational interaction seems so feeble as compared to the other fundamental interactions. The reason would be that whereas nuclear and electromagnetic interactions propagate in 3 +1 dimensions, gravity propagates in *D* dimensions. A 3 + 1-dimensional application of Gauss’s law then yields an incomplete account of the total gravitational flux. Thus, the apparent (3 + 1-dimensional) gravitational coupling appears smaller than the real (*D*-dimensional) one. Or, equivalently, the real fundamental Planck energy scale becomes much smaller than the apparent one. An estimate is obtained considering the *D*-dimensional gravitational action and integrating the compact dimensions by assuming the metric is independent of them:

8 $${\mathcal S}{\propto}{1 \over {{G_D}}}\int {{{\rm{d}}^D}x\sqrt {{}^Dg}} {}^DR = {{{V_{D - 4}}} \over {{G_D}}}\int {{{\rm{d}}^4}x} \sqrt {{}^4g} {}^4R,$$

thus the four-dimensional Newton’s constant is related to the *D*-dimensional one by the volume of the compact dimensions *G*_4_ = *G*_*D*_/*V*_*D*−4_.

In units such that *c* = *ħ* =1 (different from the units *G* = *c* =1 used in the rest of this paper), the mass-energy Planck scale in four dimensions $E_{{\rm{Planck}}}^{(4)}$ is related to Newton’s constant by ${G_4} = {(E_{{\rm{Planck}}}^{(4)})^{- 2}}$, since $\int {\rm d}^{4}x \sqrt {\ ^{4}g}\ ^{4}R$ has the dimension of length squared; similar dimensional arguments in Eq. (8) show that in *D* dimensions ${G_D} = {(E_{{\rm{Planck}}}^{(D)})^{- (D - 2)}}$. Therefore, the *D*-dimensional Planck energy $E_{{\rm{Planck}}}^{(D)}$ is related to the four-dimensional one by

9 $${{E_{{\rm{Planck}}}^{(D)}} \over {E_{{\rm{Planck}}}^{(4)}}} = {\left({{1 \over {{{(E_{{\rm{Planck}}}^{(4)})}^{D - 4}}{V_{D - 4}}}}} \right)^{{1 \over {D - 2}}}} = {\left({{{{{(L_{{\rm{Planck}}}^{(4)})}^{D - 4}}} \over {{V_{D - 4}}}}} \right)^{{1 \over {D - 2}}}},$$

where we have defined the four-dimensional Planck length as $L_{{\rm{Planck}}}^{(4)} = 1/E_{{\rm{Planck}}}^{(4)}$. For instance, for *D* = 10 and taking the six extra dimensions of the order of the Fermi, Eq. (9) shows that the fundamental Planck scale would be of the order of a TeV. For a more detailed account of the braneworld scenario, we refer the reader to the reviews [658, 532].

The real fundamental Planck scale sets the regime in particle physics beyond which gravitational phenomena cannot be ignored and actually become dominant [736]; this is the trans-Planckian regime in which particle collisions lead to BH formation and sizeable GW emission. A Planck scale at the order of TeV (*TeV gravity scenario*) could then imply BH formation in particle accelerators, such as the LHC, or in ultra high-energy cosmic rays [69, 279, 353]. Well into the trans-Planckian regime, i.e., for energies significantly larger than the Planck scale, classical gravity described by GR in *D*-dimensions is the appropriate description for these events, since the formed BHs are large enough so that quantum corrections may be ignored on and outside the horizon.

In this scenario, phenomenological signatures for BH formation would be obtained from the Hawking evaporation of the micro BHs, and include a large multiplicity of jets and large transverse momentum as compared to standard model backgrounds [1]. Preliminary searches of BH formation events in the LHC data have already been carried out, considering *pp* collisions with center-of-mass energies up to 8 TeV; up to now, no evidence of BH creation has been found [201, 3, 202, 4]. To filter experimental data from particle colliders, Monte Carlo event generators have been coded, e.g., [336], which need as input the cross section for BH formation and the inelasticity in the collisions (gravitationally radiated energy). The presently used values come from apparent horizon (AH) estimates, which in *D* = 4 are known to be off by a factor of 2 (at least). In *D*-dimensions, these values must be obtained from numerical simulations colliding highly boosted lumps of energy, BHs or shock waves, since it is expected that in this regime ‘matter does not matter’; all that matters is the amount of gravitational charge, i.e., energy, carried by the colliding objects.

## Exact Analytic and Numerical Stationary Solutions

Any numerical or analytic analysis of dynamical processes must start with a careful analysis of the static or stationary solutions underlying those dynamics. In GR this is particularly relevant, as stationary solutions are known and have been studied for many decades, and important catalogs have been built. Furthermore, stationary solutions are also relevant in a NR context: they can be used as powerful benchmarks, initial data for nonlinear evolutions, and as a final state reference to interpret results. We now briefly review some of the most important, and recent, work on the subject directly relevant to ongoing NR efforts. This Section does not dispense with the reading of other reviews on the subject, for instance Refs. [727, 308, 438, 790].

### Exact solutions

#### Four-dimensional, electrovacuum general relativity with Λ

Exact solutions of a nonlinear theory, such as GR, provide invaluable insights into the physical properties of the theory. Finding such solutions analytically and through a direct attack, that is by inserting an educated ansatz into the field equations, can be a *tour de force*, and, in general, only leads to success if a large isometry group is assumed from the beginning for the spacetime geometry. For instance, assuming spherical symmetry, in vacuum, leads to a fairly simple problem, whose general solution is the Schwarzschild metric [687]. This simplicity is intimately connected with the inexistence of a spherically symmetric mode for gravitational radiation in Einstein gravity, which means that, in vacuum, a spherically symmetric solution must be static, as recognized by Birkhoff [103]. On the other hand, assuming only axial symmetry leads to a considerably more difficult problem, even under the additional assumption of staticity. This problem was first considered by Weyl [776] who unveiled a curious and helpful mapping from these solutions to axially symmetric solutions of Newtonian gravity in an auxiliary 3-dimensional flat space; under this mapping, a solution to the latter problem yields a solution to the vacuum Einstein equations: *a Weyl solution*. For instance, the Schwarzschild solution of mass *M* can be recovered as a Weyl solution from the Newtonian gravitational field of an infinitely thin rod of linear density 1/2 and length 2 *M*. As we shall discuss in Section 4.1.2, the generalization of Weyl solutions plays an important role in the construction of qualitatively new solutions to the higher-dimensional Einstein equations.

Within the axially symmetric family of solutions, the most interesting case from the astrophysical viewpoint is the solution for a rotating source, which could describe the gravitational field exterior to a rotating star or the one of a rotating BH. An exact solution of Einstein’s equations describing the exterior of a rotating star has not been found (rotating stars are described using perturbative and numerical approaches [728]),^5^ but in the case of a rotating BH, such a solution does exist. To obtain this stationary, rather than static, geometry, the Weyl approach by itself is unhelpful and new methods had to be developed. These new methods started with Petrov’s work on the classification of the Weyl tensor types [616]. The Weyl tensor determines four null complex ‘eigenvectors’ at each point, and the spacetime is called ‘algebraically special’ if at least two of these coincide. Imposing the algebraically special condition has the potential to reduce the complicated nonlinear PDEs in two variables, obtained for a vacuum axially symmetric stationary metric, to ordinary differential equations. Using the (then) recently shown Goldberg-Sachs theorem [357], Kerr eventually succeeded in obtaining the celebrated Kerr metric in 1963 [466]. This family of solutions was generalized to include charge by Newman et al. — the Kerr-Newman solution [576] — and to include a cosmological constant by Carter [187]. In Boyer-Lindquist coordinates, the Kerr-Newman-(A)dS metric reads:

10 $${\rm{d}}{s^2} = - {{{\Delta _r}} \over {{\rho ^2}}}{\left[ {{\rm{d}}t - {{a{{\sin}^2}\theta} \over \Sigma}{\rm{d}}\phi} \right]^2} + {{{\rho ^2}} \over {{\Delta _r}}}{\rm{d}}{r^2} + {{{\rho ^2}} \over {{\Delta _\theta}}}{\rm{d}}{\theta ^2} + {{{\Delta _\theta}{{\sin}^2}\theta} \over {{\rho ^2}}}{\left[ {a\;{\rm{d}}t - {{{r^2} + {a^2}} \over \Sigma}{\rm{d}}\phi} \right]^2},$$

where

11 $${\rho ^2} = {r^2} + {a^2}{\cos ^2}\theta, \quad \Sigma = 1 + {{{a^2}\Lambda} \over 3},$$

12 $${\Delta _r} = ({r^2} + {a^2})\left({1 - {{{r^2}\Lambda} \over 3}} \right) - 2Mr + {Q^2} + {P^2},\quad {\Delta _\theta} = 1 + {{{a^2}\Lambda} \over 3}{\cos ^2}\theta.$$

Here, *M*, *aM*, *Q*, *P*, Λ are respectively, the BH mass, angular momentum, electric charge, magnetic charge and cosmological constant.

At the time of its discovery, the Kerr metric was presented as an example of a stationary, axisymmetric (BH) solution. The outstanding importance of the Kerr metric was only realized some time later with the establishment of the *uniqueness theorems* [188, 654]: the only asymptotically flat, stationary and axisymmetric, electrovacuum solution to the Einstein equations, which is nonsingular on and outside an event horizon is the Kerr-Newman geometry. Moreover, Hawking’s rigidity theorem [406] made the axisymmetric assumption unnecessary: a stationary BH must indeed be axisymmetric. Although the stability of the Kerr metric is not a closed subject, the bottom line is that it is widely believed that the final equilibrium state of the gravitational collapse of an enormous variety of different stars is described by the Kerr geometry, since the electric charge should be astrophysically negligible. If true, this is indeed a truly remarkable fact (see, however, Section 4.2 for “hairy” BHs).

Even if we are blessed to know precisely the metric that describes the final state of the gravitational collapse of massive stars or of the merger of two BHs, the geometry of the time-dependent stages of these processes seems desperately out of reach as an exact, analytic solution. To understand these processes we must then resort to approximate or numerical techniques.

#### Beyond four-dimensional, electrovacuum general relativity with Λ

As discussed in Section 3 there are various motivations to consider generalizations of (or alternative theories to) four-dimensional electrovacuum GR with A. A natural task is then to address the exact solutions of such theories. Here we shall briefly address the exact solutions in two different classes of modifications of Einstein electrovacuum gravity: i) changing the dimension, *D* ≠ 4; ii) changing the equations of motion, either by changing the right-hand side — i.e., theories with different matter fields, including non-minimally coupled ones —, or by changing the left-hand side — i.e., higher curvature gravity. We shall focus on relevant solutions for the topic of this review article, referring to the specialized literature where appropriate.

- Changing the number of dimensions: GR in *D* ≠ 4. Exact solutions in *higher-dimensional* GR, *D* > 4, have been explored intensively for decades and an excellent review on the subject is Ref. [308]. In the following we shall focus on the vacuum case.
  The first classical result is the *D* > 4 generalization of the Schwarzschild BH, i.e., the vacuum, spherically — that is *SO*(*D* − 1) — symmetric solution to the *D*-dimensional Einstein equations (with or without cosmological constant), obtained by Tangherlini [740] in the same year the Kerr solution was found. Based on his solution, Tangherlini suggested an argument to justify the (apparent) dimensionality of spacetime. But apart from this insight, the solution is qualitatively similar to its four-dimensional counterpart: an analog of Birkhoff’s theorem holds and it is perturbatively stable.
  On the other hand, the existence of extra dimensions accommodates a variety of extended objects with *reduced* spherical symmetry — that is *SO*(*D* − 1 − *p*) — surrounded by an event horizon, generically dubbed as *p-branes*, where *p* stands for the spatial dimensionality of the object [441, 285]. Thus, a point-like BH is a 0-brane, a string is a 1-brane and so on. The charged counterparts of these objects have played a central role in SMT, especially when charged under a type of gauge field called ‘Ramond-Ramond’ fields, in which case they are called *Dp*-branes or simply *D*-branes [284]. Here we wish to emphasize that the Gregory-Laflamme instability discussed in Section 3.2.4 was unveiled in the context of *p*-branes, in particular black strings [367, 368]. The understanding of the nonlinear development of such instability is a key question requiring numerical techniques.
  The second classical result was the generalization of the Kerr solution to higher dimensions, i.e., a vacuum, stationary, axially — that is^6^ $SO{(2)^{[{{D - 1} \over 2}]}}$ — symmetric solution to the *D*-dimensional Einstein equations, obtained in 1986 by Myers and Perry [565] (and later generalized to include a cosmological constant [351, 350]). The derivation of this solution was quite a technical achievement, made possible by using a Kerr-Schild type ansatz. The solution exhibits a number of new qualitative features, in particular in what concerns its stability. It has $[{{D - 1} \over 2}]$ independent angular momentum parameters, due to the nature of the rotation group in *D* dimensions. If only one of these rotation parameters is non-vanishing, i.e., for the singly spinning Myers-Perry solution, in dimensions *D* ≥ 6 there is no bound on the angular momentum *J* in terms of the BH mass *M*. *Ultra-spinning* Myers-Perry BHs are then possible and their horizon appears highly deformed, becoming locally analogous to that of a *p*-brane. This similarity suggests that ultra-spinning BHs should suffer from the Gregory-Laflamme instability. Entropic arguments also support the instability of these BHs [305] (see Section 7.4 for recent developments).
  The third classical result was the recent discovery of the black ring in *D* = 5 [307], a black object with a non-simply connected horizon, having spatial sections that are topologically *S*^2^ × *S*^1^. Its discovery raised questions about how the *D* = 4 results on uniqueness and stability of vacuum solutions generalized to higher-dimensional gravity. Moreover, using the generalization to higher dimensions of Weyl solutions [306] and of the inverse scattering technique [394], geometries with a non-connected event horizon — i.e., multi-object solutions — which are asymptotically flat, regular on and outside an event horizon have been found, most notably the black Saturn [303]. Such solutions rely on the existence of black objects with non-spherical topology; regular multi-object solutions with only Myers-Perry BHs do not seem to exist [425], just as regular multi-object solutions with only Kerr BHs in *D* = 4 are inexistent [574, 424].
  Let us briefly mention that BH solutions in *lower dimensional* GR have also been explored, albeit new ingredients are necessary for such solutions to exist. *D* = 3 vacuum GR has no BH solutions, a fact related to the lack of physical dimensionality of the would be Schwarzschild radius *MG*^(3)^, where *G*^(3)^ is the 3-dimensional Newton’s constant. The necessary extra ingredient is a negative cosmological constant; considering it leads to the celebrated Bañados-Teitelboim-Zanelli (BTZ) BH [68]. In *D* = 2 a BH spacetime was obtained by Callan, Giddings, Harvey and Strominger (the CGHS BH), by considering GR non-minimally coupled to a scalar field theory [156]. This solution provides a simple, tractable toy model for numerical investigations of dynamical properties; for instance see [55, 54] for a numerical study of the evaporation of these BHs.
- Changing the equations: Different matter fields and higher curvature gravity.
  The uniqueness theorems of four-dimensional electrovacuum GR make clear that BHs are selective objects. Their equilibrium state only accommodates a specific gravitational field, as is clear, for instance, from its constrained multipolar structure. In enlarged frameworks where other matter fields are present, this selectiveness may still hold, and various “no-hair theorems” have been demonstrated in the literature, i.e., proofs that under a set of assumptions no stationary regular BH solutions exist, supporting (nontrivial) specific types of fields. A prototypical case is the set of no-hair theorems for asymptotically flat, static, spherically symmetric BHs with scalar fields [546]. Note, however, that hairy BHs, do exist in various contexts, cf. Section 4.2.
  The inexistence of an exact stationary BH solution, i.e., of an equilibrium state, supporting (say) a specific type of scalar field does not mean, however, that a scalar field could not exist long enough around a BH so that its effect becomes relevant for the observed dynamics. To analyse such possibilities dynamical studies must be performed, typically involving numerical techniques, both in linear and nonlinear analysis. A similar discussion applies equally to the study of scalar-tensor theories of gravity, where the scalar field may be regarded as part of the gravitational field, rather than a matter field. Technically, these two perspectives may be interachanged by considering, respectively, the Jordan or the Einstein frame. The emission of GWs in a binary system, for instance, may depend on the ‘halo’ of other fields surrounding the BH and therefore provide smoking guns for testing this class of alternative theories of gravity.
  Finally, the change of the left-hand side of the Einstein equations may be achieved by considering higher curvature gravity, either motivated by ultraviolet corrections to GR, i.e., changing the theory at small distance scales, such as Gauss-Bonnet [844] (in *D* ≥ 5), Einstein-Dilaton-Gauss-Bonnet gravity and Dynamical Chern-Simons gravity [602, 27]; or infrared corrections, changing the theory at large distance scales, such as certain *f* (*R*) models. This leads, generically, to modifications of the exact solutions. For instance, the spherically symmetric solution to Gauss-Bonnet theory has been discussed in Ref. [120] and differs from, but asymptotes to, the Tangherlini solution. In specific cases, the higher curvature model may share some GR solutions. For instance, Chern-Simons gravity shares the Schwarzschild solution but not the Kerr solution [27]. Dynamical processes in these theories are of interest but their numerical formulation, for fully nonlinear processes, may prove challenging or even, apart from special cases (see, e.g., [265] for a study of critical collapse in Gauss-Bonnet theory), ill-defined.

#### State of the art

- *D* ≠ 4: The essential results in higher-dimensional vacuum gravity are the Tangherlini [740] and Myers-Perry [565] BHs, the (vacuum) black *p*-branes [441, 285] and the Emparan-Reall black ring [307]. Solutions with multi-objects can be obtained explicitly in *D* = 5 with the inverse scattering technique. Their line element is typically quite involved and given in Weyl coordinates (see [308] for a list and references). The Myers-Perry geometry with a cosmological constant was obtained in *D* = 5 in Ref. [407] and for general *D* and cosmological constant in [351, 350]. Black rings have been generalized, as numerical solutions, to higher *D* in Ref. [472]. Black p-branes have been discussed, for instance, in Ref. [441, 285]. In *D* = 3, 2 the best known examples of BH solutions are, respectively, the BTZ [68] and the CGHS BHs [156].
- Changing the equations of motion: Hawking showed [404] that in Brans-Dicke gravity the only stationary BH solutions are the same as in GR. This result was recently extended by Sotiriou and Faraoni to more general scalar-tensor theories [712]. Such type of no-hair statements have also been proved for spherically symmetric solutions in GR (non-)minimally coupled to scalar fields [85] *and* to the electromagnetic field [546]; but they are not universal: for instance, a harmonic time dependence for a (complex) scalar field or a generic potential (together with gauge fields) are ways to circumvent these results (see Section 4.2 and e.g. the BH solutions in [352]). BHs with scalar hair have also been recently argued to exist in generalized scalar-tensor gravity [713].

### Numerical stationary solutions

Given the complexity of the Einstein equations, it is not surprising that, in many circumstances, *stationary* exact solutions cannot be found in closed analytic form. In this subsection we shall very briefly mention numerical solutions to such *elliptic problems* for cases relevant to this review.

The study of the Einstein equations coupled to *nonlinear matter sources* must often be done numerically, even if stationarity and spatial symmetries — typically spherical or axisymmetry — are imposed.^7^ The study of numerical solutions of elliptic problems also connects to research on soliton-like solutions in nonlinear field theories without gravity. Some of these solitons can be promoted to *gravitating solitons* when gravity is included. Skyrmions are one such case [107]. In other cases, the nonlinear field theory does not have solitons but, when coupled to gravity, gravitating solitons arise. This is the case of the Bartnik-McKinnon particle-like solutions in Einstein-Yang-Mills theory [77]. Moreover, for some of these gravitating solitons it is possible to include a BH at their centre giving rise to “hairy BHs”. For instance, in the case of Einstein-Yang-Mills theory, these have been named “colored BHs” [105]. We refer the reader interested in such gravitating solitons connected to hairy BHs to the review by Bizoń [106] and to the paper by Ashtekar et al. [51].

A particularly interesting type of gravitating solitons are *boson stars* (see [685, 516] for reviews), which have been suggested as BH mimickers and dark matter candidates. These are solutions to Einstein’s gravity coupled to a complex massive scalar field, which may, or may not, have self-interactions. Boson stars are horizonless gravitating solitons kept in equilibrium by a balance between their self-generated gravity and the dispersion effect of the scalar field’s wave-like character. All known boson star solutions were obtained numerically; and both static and rotating configurations are known. The former ones have been used in numerical high energy collisions to model particles and test the hoop conjecture [216] (see Section 7.3 and also Ref. [599] for earlier boson star collisions and [561] for a detailed description of numerical studies of boson star binaries). The latter ones have been shown to connect to rotating BHs, both for *D* = 5 Myers-Perry BHs in AdS [275] and for *D* = 4 Kerr BHs [422], originating families of rotating BHs with scalar hair. Crucial to these connections is the phenomenon of superradiance (see Section 7.5), which also afflicts rotating boson stars [182]. The BHs with scalar hair branch off from the Kerr or Myers-Perry-AdS BHs precisely at the threshold of the superradiant instability for a given scalar-field mode [423], and display new physical properties, e.g., new shapes of ergo-regions [419].

The situation we have just described, i.e., the branching off of a solution to Einstein’s field equations into a new family at the onset of a classical instability, is actually a recurrent situation. An earlier and paradigmatic example — occurring for the vacuum Einstein equations in higher dimensions — is the branching off of black strings at the onset of the Gregory-Laflamme instability [367] (see Section 3.2.4 and Section 7.2) into a family of non-uniform black strings. The latter were found numerically by Wiseman [789] following a perturbative computation by Gubser [371]. We refer the reader to Ref. [470] for more non-uniform string solutions, to Refs. [11, 790] for a discussion of the techniques to construct these numerical (vacuum) solutions and to [442] for a review of (related) Kaluza-Klein solutions. Also in higher dimensions, a number of other numerical solutions have been reported in recent years, most notably generalizations of the Emparan-Reall black ring [474, 472, 473] and BH solutions with higher curvature corrections (see, e.g., [131, 475, 132]). Finally, numerical rotating BHs with higher curvature corrections but in *D* = 4, within dilatonic Einstein-Gauss-Bonnet theory, were reported in [471].

In the context of holography (see Section 3.3.1 and Section 7.8), numerical solutions have been of paramount importance. Of particular interest to this review are the hairy AdS BHs that play a role in the AdS-Condensed matter duality, by describing the superconducting phase of holographic superconductors. These were first constructed (numerically) in [398]. See also the reviews [396, 436] for further developments.

In the context of Randall-Sundrum scenarios, large BHs were first shown to exist via a numerical calculation [318], and later shown to agree with analytic expansions [8].

Finally, let us mention, as one application to mathematical physics of numerical stationary solutions, the computation of Ricci-flat metrics on Calabi-Yau manifolds [409].

## Approximation Schemes

The exact and numerically-constructed stationary solutions we outlined above are, as a rule, objects that can also have interesting dynamics. A full understanding of these dynamics is the subject of NR, but before attempting fully nonlinear evolutions of the field equations, approximations are often useful. These work as benchmarks for numerical evolutions, as order-of-magnitude estimates and in some cases (for example extreme mass ratios) remain the only way to attack the problem, as it becomes prohibitively costly to perform full nonlinear simulations, see Figure 1. The following is a list of tools, techniques, and results that have been instrumental in the field. For an analysis of approximation schemes and their interface with NR in four-dimensional, asymptotically flat spacetimes, see Ref. [502].

### Post-Newtonian schemes

#### Astrophysical systems in general relativity

For many physical phenomena involving gravity, GR predicts small deviations from Newtonian gravity because for weak gravitational fields and low velocities Einstein’s equations reduce to the Newtonian laws of physics. Soon after the formulation of GR, attempts were therefore made (see, e.g., [295, 254, 522, 298, 324, 606, 619, 194, 292]) to express the dynamics of GR as deviations from the Newtonian limit in terms of an expansion parameter *ϵ*. This parameter can be identified, for instance, with the typical velocities of the matter composing the source, or with the compactness of the source:

13 $$\epsilon \sim {v \over c} \sim \sqrt {{{GM} \over {r{c^2}}}},$$

which uses the fact that, for bound systems, the virial theorem implies *υ*^2^ ∼ *GM*/*r*. In this approach, called “post-Newtonian”, the laws of GR are expressed in terms of the quantities and concepts of Newtonian gravity (velocity, acceleration, etc.). A more rigorous definition of the parameter *ϵ* can be found elsewhere [109], but as a book-keeping parameter it is customary to consider *ϵ* = *υ*/*c*. The spacetime metric and the stress-energy tensor are expanded in powers of *ϵ* and terms of order *ϵ*^*n*^ are commonly referred to as (*n*/2)-PN corrections. The spacetime metric and the motion of the source are found by solving, order by order, Einstein’s equations.

Strictly speaking, the PN expansion can only be defined in the near zone, which is the region surrounding the source, with dimensions much smaller than the wavelength *λ*_*Gw*_ of the emitted GWs. Outside this region, and in particular in the wave zone (e.g., at a distance ≫ *λ*_*gw*_ from the source), radiative processes make the PN expansion ill-defined, and different approaches have to be employed, such as the post-Minkowskian expansion, which assumes weak fields but not slow motion. In the post-Minkowskian expansion the gravitational field, described by the quantities ${h^{\alpha \beta}} = {\eta ^{\alpha \beta}} - \sqrt {- g} {g^{\alpha \beta}}$ (in harmonic coordinates, such that ${h^{\mu \nu}}_{,\nu} = 0$) is formally expanded in powers of Newton’s constant *G*. Using a variety of different tools (PN expansion in the near zone, post-Minkowskian expansion in the wave zone, multipolar expansions, regularization of point-like sources, etc.), it is possible to solve Einstein’s equations, and to determine both the motion of the source and its GW emission. Since each term of the post-Minkowskian expansion can itself be PN-expanded, the final output of this computation has the form of a PN expansion; therefore, these methods are commonly referred to as *PN approximation schemes*.

PN schemes are generally used to study the motion of *N*-body systems in GR, and to compute the GW signal emitted by these systems. More specifically, most of the results obtained so far with PN schemes refer to the *relativistic two-body problem*, which can be applied to study compact binary systems formed by BHs and/or NSs (see Section 3.1.1). In the following we shall provide a brief summary of PN schemes, their main features and results as applied to the study of compact binary systems. For a more detailed description, we refer the reader to one of the many reviews that have been written on the subject; see e.g. [109, 620, 676, 454].

Two different but equivalent approaches have been developed to solve the relativistic two-body problem, finding the equations of motion of the source and the emitted gravitational waveform: the multipolar post-Minkowskian approach of Blanchet, Damour and Iyer [109], and the direct integration of the relaxed Einstein’s equations, developed by Will and Wiseman [785]. In these approaches, Einstein’s equations are solved iteratively in the near zone, employing a PN expansion, and in the wave zone, through a post-Minkowskian expansion. In both cases, multipolar expansions are performed. The two solutions, in the near and in the wave zone, are then matched. These approaches yield the equations of motion of the bodies, i.e., their accelerations as functions of their positions and velocities, and allow the energy balance equation of the system to be written as

14 $${{{\rm{d}}E} \over {{\rm{d}}t}} = - {\mathcal L}.$$

Here, *E* (which depends on terms of integer PN orders) can be considered as the energy of the system), and ${\mathcal L}$ (depending on terms of half-integer PN orders) is the emitted GW flux. The lowest PN order in the GW flux is given by the quadrupole formula [297] (see also [553]), $\mathcal L = G/(5{c^5})({Q_{ab}}{Q_{ab}} + O(1/{c^3}))$ where *Q*_*ab*_ is the (traceless) quadrupole moment of the source. The leading term in C is then of 2.5-PN order (i.e., ∼ 1/c^5^), but since *Q*_*ab*_ is computed in the Newtonian limit, it is often considered as a “Newtonian” term. A remarkable result of the multipolar post-Minkowskian approach and of the direct integration of relaxed Einstein’s equations, is that once the equations are solved at *n*-th PN order both in the near zone and in the wave zone, *E* is known at *n*-PN order, and ${\mathcal L}$ is known at *n*-PN order with respect to its leading term, i.e., at (*n* + 2.5)-PN order. Once the energy and the GW flux are known with this accuracy, the gravitational waveform can be determined, in terms of them, at *n*-PN order.

Presently, PN schemes determine the motion of a compact binary, and the emitted gravitational waveform, up to 3.5-PN order for non-spinning binaries in circular orbits [109], but up to lower PN-orders for eccentric orbits and for spinning binaries [48, 148]. It is estimated that Advanced LIGO/Virgo data analysis requires 3.5-PN templates [123], and therefore some effort still has to go into the modeling of eccentric orbits and spinning binaries. It should also be remarked that the state-of-the-art PN waveforms have been compared with those obtained with NR simulations, showing a remarkable agreement in the inspiral phase (i.e., up to the late inspiral stage) [122, 389].

An alternative to the schemes discussed above is the ADM-Hamiltonian approach [676], in which using the ADM formulation of GR, the source is described as a canonical system in terms of its Hamiltonian. The ADM-Hamiltonian approach is equivalent to the multipolar post-Minkowskian approach and to the direct integration of relaxed Einstein’s equations, as long as the evolution of the source is concerned [246], but since Einstein’s equations are not solved in the wave zone, the radiative effects are only known with the same precision as the motion of the source. This framework has been extended to spinning binaries (see [726] and references therein). Recently, an alternative way to compute the Hamiltonian of a post-Newtonian source has been developed, the effective field theory approach [358, 149, 627, 340], in which techniques originally derived in the framework of quantum field theory are employed. This approach was also extended to spinning binaries [626, 625]. ADM-Hamiltonian and effective field theory are probably the most promising approaches to extend the accuracy of PN computations for spinning binaries.

The effective one-body (EOB) approach developed at the end of the last century [147] and recently improved [247, 600] (see, e.g., [240, 249] for a more detailed account) is an extension of PN schemes, in which the PN Taylor series is suitably resummed, in order to extend its validity up to the merger of the binary system. This approach maps the dynamics of the two compact objects into the dynamics of a single test particle in a deformed Kerr spacetime. It is a canonical approach, so the Hamiltonian of the system is computed, but the radiative part of the dynamics is also described. Since the mapping between the two-body system and the “dual” one-body system is not unique, the EOB Hamiltonian depends on a number of free parameters, which are fixed using results of PN schemes, of gravitational self-force computations, and of NR simulations. After this calibration, the waveforms reproduce with good accuracy those obtained in NR simulations (see, e.g., [240, 249, 600, 61]). In the same period, a different approach has been proposed to extend PN templates to the merger phase, matching PN waveforms describing the inspiral phase, with NR waveforms describing the merger [17, 673]. Both this “phenomenological waveform” approach and the EOB approach use results from approximation schemes and from NR simulations in order to describe the entire waveform of coalescing binaries, and are instrumental for data analysis [584].

To conclude this section, we mention that PN schemes originally treated compact objects as point-like, described by delta functions in the stress-energy tensor, and employing suitable regularization procedures. This is appropriate for BHs, and, as a first approximation, for NSs, too. Indeed, finite size effects are formally of 5-PN order (see, e.g., [239, 109]). However, their contribution can be larger than what a naive counting of PN orders may suggest [557]. Therefore, the PN schemes and the EOB approach have been extended to include the effects of tidal deformation of NSs in compact binary systems and on the emitted gravitational waveform using a set of parameters (the “Love numbers”) encoding the tidal deformability of the star [323, 248, 760, 102].

#### Beyond general relativity

PN schemes are also powerful tools to study the nature of the gravitational interaction, i.e., to describe and design observational tests of GR. They have been applied either to build general parametrizations, or to determine observable signatures of specific theories (two kinds of approaches that have been dubbed *top-down* and *bottom-up*, respectively [636]).

Let us discuss *top-down* approaches first. Nearly fifty years ago, Will and Nordtvedt developed the PPN formalism [784, 581], in which the PN metric of an *N*-body system is extended to a more general form, depending on a set of parameters describing possible deviations from GR. This approach (which is an extension of a similar approach by Eddington [291]) facilitates tests of the weak-field regime of GR. It is particularly well suited to perform tests in the solar system. All solar-system tests can be expressed in terms of constraints on the PPN parameters, which translates into constraints on alternative theories of gravity. For instance, the measurement of the Shapiro time-delay from the Cassini spacecraft [99] yields the strongest bound on one of the PPN parameters; this bound determines the strongest constraint to date on many modifications of GR, such as Brans-Dicke theory.

More recently, a different parametrized extension of the PN formalism has been proposed which, instead of the PN metric, expands the gravitational waveform emitted by a compact binary inspiral in a set of parameters describing deviations from GR [825, 203]. The advantage of this so-called “parametrized post-Einsteinian” approach — which is different in spirit from the PPN expansion, since it does not try to describe the spacetime metric — is its specific design to study the GW output of compact binary inspirals which are the most promising sources for GW detectors (see Section 3.1.1).

As mentioned above, PN approaches have also been applied *bottom-up*, i.e., in a manner that directly calculates the observational consequences of specific theories. For instance, the motion of binary pulsars has been studied, using PN schemes, in specific alternative theories of gravity, such as scalar-tensor theories [244]. The most promising observational quantity to look for evidence of GR deviations is probably the gravitational waveform emitted in compact binary inspirals, as computed using PN approaches. In the case of theories with additional fundamental fields, the leading effect is the increase in the emitted gravitational flux arising from the additional degrees of freedom. This increase typically induces a faster inspiral, which affects the phase of the gravitational waveform (see, e.g., [91]). For instance, in the case of scalar-tensor theories a dipolar component of the radiation can appear [787]. In other cases, as in massive graviton theories, the radiation has ℓ ≥ 2 as in GR, but the flux is different. For further details, we refer the interested reader to [782] and references therein.

#### State of the art

The post-Newtonian approach has mainly been used to study the relativistic two-body problem, i.e., to study the motion of compact binaries and the corresponding GW emission. The first computation of this kind, at leading order, was done by Peters and Mathews for generic eccentric orbits [614, 613]. It took about thirty years to understand how to extend this computation at higher PN orders, consistently modeling the motion and the gravitational emission of a compact binary [109, 785]. The state-of-the-art computations give the gravitational waveform emitted by a compact binary system, up to 3.5-PN order for non-spinning binaries in circular orbits [109], up to 3-PN order for eccentric orbits [48], and up to 2-PN order for spinning binaries [148]. An alternative approach, based on the computation of the Hamiltonian [676], is currently being extended to higher PN orders [726, 457, 399]; however, in this approach the gravitational waveform is computed with less accuracy than the motion of the binary.

Recently, different approaches have been proposed to extend the validity of PN schemes up to the merger, using results from NR to fix some of the parameters of the model (as in the EOB approach [249, 600, 61, 240]), or matching NR with PN waveforms (as in the “phenomenological waveform” approach [17, 673]). PN and EOB approaches have also been extended to include the effects of tidal deformation of NSs [323, 248, 760, 102].

PN approaches have been extended to test GR against alternative theories of gravity. Some of these extensions are based on a parametrization of specific quantities, describing possible deviations from GR. This is the case in the PPN approach [784, 581], most suitable for solar-system tests (see [782, 783] for extensive reviews on the subject), and in the parametrized post-Einsteinian approach [825, 203], most suitable for the analysis of data from GW detectors. Other extensions, instead, start from specific alternative theories and compute — using PN schemes — their observational consequences. In particular, the motion of compact binaries and the corresponding gravitational radiation have been extensively studied in scalar-tensor theories [244, 787, 30].

### Spacetime perturbation approach

#### Astrophysical systems in general relativity

The PN expansion is less successful at describing strong-field, relativistic phenomena. Different tools have been devised to include this regime and one of the most successful schemes consists of describing the spacetime as a small deviation from a known exact solution. Systems well described by such a perturbative approach include, for instance, the inspiral of a NS or a stellar-mass BH of mass *μ* into a supermassive BH of mass *M* ≫ *μ* [354, 32], or a BH undergoing small oscillations around a stationary configuration [487, 316, 95].

In this approach, the spacetime is assumed to be, at any instant, a small deviation from the background geometry, which, in the cases mentioned above, is described by the Schwarzschild or the Kerr solution here denoted by $g_{\mu \nu}^{(0)}$. The deformed spacetime metric ${g_{\mu \nu}}$ can then be decomposed as

15 $${g_{\mu \nu}} = g_{\mu \nu}^{(0)} + {h_{\mu \nu}},$$

where *h*_*μν*_ ≪ 1 describes a small perturbation induced by a small object or by any perturbing event.^8^ Einstein’s equations are linearized around the background solution, by keeping only first-order terms in *h*_*μν*_ (and in the other perturbation quantities, if present).

The simple expansion (15) implies a deeper geometrical construction (see, e.g., [730]), in which one considers a family of spacetime manifolds ${\mathcal M}_{\lambda}$, parametrized by a parameter λ; their metrics g(λ) satisfy Einstein’s equations, for each λ. The λ = 0 element of this family is the background spacetime, and the first term in the Taylor expansion in λ is the perturbation. Therefore, in the spacetime perturbation approach it is the spacetime manifold itself to be perturbed and expanded. However, once the perturbations are defined (and the gauge choice, i.e., the mapping between quantities in different manifolds, is fixed), perturbations can be treated as genuine fields living on the background spacetime ${\mathcal M_o}$. In particular, the linearized Einstein equations can be considered as linear equations on the background spacetime, and all the tools to solve linear differential equations on a curved manifold can be applied.

The real power of this procedure comes into play once one knows how to separate the angular dependence of the perturbations *h*_*μν*_. This was first addressed by Regge and Wheeler in their seminal paper [641], where they showed that in the case of a Schwarzschild background, the metric perturbations can be expanded in tensor spherical harmonics [541], in terms of a set of perturbation functions which only depend on the coordinates *t* and *r*. They also noted that the terms of this expansion belong to two classes (even and odd perturbations, sometimes also called polar and axial), with different behaviour under parity transformations (i.e., *θ → π − θ*, *ϕ → ϕ* + *π*). The linearized Einstein equations, expanded in tensor harmonics, yield the dynamical equations for the perturbation functions. Furthermore, perturbations corresponding to different harmonic components or different parities decouple due to the fact that the background is spherically symmetric. After a Fourier transformation in time, the dynamical equations reduce to ordinary differential equations in *r*.

Regge and Wheeler worked out the equations for axial perturbations of Schwarzschild BHs; later on, Zerilli derived the equations for polar perturbations [830]. With their gauge choice (the “Regge-Wheeler gauge”, which allows us to set to zero some of the perturbation functions), the harmonic expansion of the metric perturbation is

16 $${h_{\mu \nu}}(t,r,\theta, \phi) = \sum\limits_{l,m} {\int\nolimits_{- \infty}^{+ \infty} {{e^{- i\omega t}}} [h_{\mu \nu}^{{\rm{ax}},lm}(\omega, r, \theta, \phi) + h_{\mu \nu}^{{\rm{pol}},lm}(\omega, r, \theta, \phi)]\;{\rm{d}}\omega}$$

with

17 $$h_{\mu \nu}^{{\rm{ax}},lm}\;{\rm{d}}{x^\mu}\;{\rm{d}}{x^\nu} = 2\;\left[ {h_0^{lm}(\omega ,r)\;{\rm{d}}t + h_1^{lm}(\omega ,r)\;{\rm{d}}r} \right]\;\,[\csc \theta {\partial _\phi}{Y_{lm}}(\theta ,\phi)\;{\rm{d}}\theta - \sin \theta {\partial _\theta}{Y_{lm}}(\theta ,\phi)\;{\rm{d}}\phi ]$$

18 $$\begin{array}{*{20}c} {h_{\mu \nu}^{{\rm{pol}},lm}\;{\rm{d}}{x^\mu}\;{\rm{d}}{x^\nu} = \left[ {f(r)H_0^{lm}(\omega ,r)\;{\rm{d}}{t^2} + 2{H_1}{{(\omega ,r)}^{lm}}\;{\rm{d}}t\;{\rm{d}}r + H_2^{lm}(\omega ,r)\;{\rm{d}}{r^2}} \right.\quad \;\;} \\ {\left. {+ {r^2}{K^{lm}}(\omega ,r)({\rm{d}}{\theta ^2} + {{\sin}^2}\theta \;{\rm{d}}{\phi ^2})} \right]\;{Y_{lm}}(\theta ,\phi),} \\ \end{array}$$

where *f*(*r*) = 1 − *2M*/*r*, and *Y*_*lm*_(*θ*, *ϕ*) are the scalar spherical harmonics.

It turns out to be possible to define a specific combination $Z_{{\rm{RW}}}^{lm}(\omega, r)$ of the axial perturbation functions $h_{\rm{0}}^{lm},h_{\rm{1}}^{lm}$, and a combination $Z_{{\rm{Zer}}}^{lm}(\omega, r)$ of the polar perturbation functions $H_{0,1,2}^{lm},\;{K^{lm}}$, *K*^*lm*^ which describe completely the propagation of GWs. These functions, called the Regge-Wheeler and the Zerilli function, satisfy Schrödinger-like wave equations of the form

19 $${{{{\rm{d}}^2}{\Psi _{{\rm{RW}},{\rm{Zer}}}}} \over {{\rm{d}}r_\ast^2}} + ({\omega ^2} - {V_{{\rm{RW}},{\rm{Zer}}}}){\Psi _{{\rm{RW}},{\rm{Zer}}}} = {{\mathcal S}_{{\rm{RW}},{\rm{Zer}}}}.$$

Here, r_*_ is the tortoise coordinate [553] and *S* represents nontrivial source terms. The energy flux emitted in GWs can be calculated straightforwardly from the solutions Ψ_RW, Zer_.

This approach was soon extended to general spherically symmetric BH backgrounds and a gauge-invariant formulation in terms of specific combinations of the perturbation functions that remain unchanged under perturbative coordinate transformations [555, 346]. In the same period, an alternative spacetime perturbation approach was developed by Bardeen, Press and Teukolsky [75, 744], based on the Newman-Penrose formalism [575], in which the spacetime perturbation is not described by the metric perturbation *h*_*μν*_, but by a set of gauge-invariant complex scalars, the Weyl scalars, obtained by projecting the Weyl tensor *C*_*αβγδ*_ onto a complex null tetrad $\ell, \;k,\;m,\;\bar m$ defined such that all their inner products vanish except $- k \;\cdot \;\ell = 1 = m \;\cdot \;\bar m$. One of these scalars, Ψ_4_, describes the (outgoing) gravitational radiation; it is defined as

20 $${\Psi _4} \equiv - {C_{\alpha \beta \gamma \delta}}{\ell ^\alpha}{\bar m^\beta}{\ell ^\gamma}{\bar m^\delta}.$$

In the literature one may also find Ψ_4_ defined without the minus sign, but all physical results derived from Ψ_4_ are invariant under this ambiguity. We further note that the Weyl and Riemann tensors are identical in vacuum. Most BH studies in NR consider vacuum spacetimes, so that we can replace *C*_*αβγδ*_ in Eq. (20) with *R*_*αβγδ*_.

In this framework, the perturbation equations reduce to a wave equation for (the perturbation of) which is called the Teukolsky equation [743]. For a general account on the theory of BH perturbations (with both approaches) see Chandrasekhar’s book [195].

The main advantage of the Bardeen-Press-Teukolsky approach is that it is possible to separate the angular dependence of perturbations of the Kerr background, even though such background is not spherically symmetric. Its main drawback is that it is very difficult to extend it beyond its original setup, i.e., perturbations of Kerr BHs. The tensor harmonic approach is much more flexible. In particular, spacetime perturbation theory (with tensor harmonic decomposition) has been extended to spherically symmetric stars [753, 518, 266, 196] (the extension to rotating stars is much more problematic [330]). As we discuss in Section 5.2.3, spacetime perturbation theory with tensor harmonic decomposition can be extended to higher-dimensional spacetimes. It is not clear whether such generalizations are possible with the Bardeen-Press-Teukolsky approach.

The sources ${\mathcal S_{{\rm{RW,Zer}}}}$ describe the objects that excite the spacetime perturbations, and can arise either directly from a non-vanishing stress-energy tensor or by imposing suitable initial conditions on the spacetime. These two alternative forms of exciting BH spacetimes have branched into two distinct tools, which can perhaps be best classified as the “point particle” [250, 179, 569, 93] and the “close limit” approximations [634, 637].

In the point particle limit the source term is a nontrivial perturbing stress-tensor, which describes for instance the infall of a small object along generic geodesics. The “small” object can be another BH, or a star, or even matter accreting into the BH. While the framework is restricted to objects of mass *μ* ≪ *M*, it is generically expected that the extrapolation to *μ ∼ M* yields at least a correct order of magnitude. Thus, the spacetime perturbation approach is in principle able to describe qualitatively, if not quantitatively, highly dynamic BHs under general conditions. The original approach treats the small test particle moving along a geodesic of the background spacetime. Gravitational back-reaction can be included by taking into account the energy and angular momentum loss of the particle due to GW emission [232, 445, 548]. More sophisticated computations are required to take into account the conservative part of the “self-force”. For a general account on the self-force problem, we refer the interested reader to the *Living Reviews* article on the subject [623]. In this approach *μ* is restricted to be a very small quantity. It has been observed by many authors [37, 718] that promoting *μ*/*M* to the symmetric mass ratio *M*_1_*M*_2_/(*M*_1_ + *M*_2_) describes surprisingly well the dynamics of generic BHs with masses *M*_1_, *M*_2_.

In the close limit approximation the source term can be traced back to nontrivial initial conditions. In particular, the original approach tackles the problem of two colliding, equal-mass BHs, from an initial separation small enough that they are initially surrounded by a common horizon. Thus, this problem can be looked at as a single perturbed BH, for which some initial conditions are known [634, 637].

A universal feature of the dynamics of BH spacetimes as given by either the point particle or the close limit approximation is that the waveform Ψ decays at late times as a universal, exponentially damped sinusoid called ringdown or QNM decay. Because at late times the forcing caused by the source term ${\mathcal S}$ has died away, it is natural to describe this phase as the free oscillations of a BH, or in other words as solutions of the homogeneous version of Eq. (19). Together with the corresponding boundary conditions, the Regge-Wheeler and Zerilli equations then describe a freely oscillating BH. In vacuum, such boundary conditions lead to an eigenvalue equation for the possible frequencies *ω*. Due to GW emission, these oscillations are damped, i.e., they have discrete, complex frequencies called *quasi-normal mode* frequencies of the BH [487, 316, 95]. Such intuitive picture of BH ringdown can be given a formally rigorous meaning through contour integration techniques [506, 95].

The extension of the Regge-Wheeler-Zerilli approach to asymptotically dS or AdS spacetimes follows with the procedure outlined above and decomposition (16); see also Ref. [176]. It turns out that the Teukolsky procedure can also be generalized to these spacetimes [192, 277, 276].

#### Beyond electrovacuum GR

The Regge-Wheeler-Zerilli approach has proved fruitful also in other contexts including alternative theories of gravity. Generically, the decomposition works by using the same metric ansatz as in Eq. (16), but now augmented to include perturbations in matter fields, such as scalar or vector fields, or further polarizations for the gravitational field. Important examples where this formalism has been applied include scalar-tensor theories [668, 165, 824], Dynamical Chern-Simons theory [175, 554, 603], Einstein-Dilaton-Gauss-Bonnet [602], Horndeski gravity [477, 478], and massive theories of gravity [135].

#### Beyond four dimensions

Spacetime perturbation theory is a powerful tool to study BHs in higher-dimensional spacetimes. The tensor harmonic approach has been successfully extended by Kodama and Ishibashi [479, 452] to GR in higher-dimensional spacetimes, with or without cosmological constant. Their approach generalizes the gauge-invariant formulation of the Regge-Wheeler-Zerilli construction to perturbations of Tangherlini’s solution describing spherically symmetric BHs.

Since many dynamical processes involving higher-dimensional BHs (in particular, the collisions of BHs starting from finite distance) can be described in the far field limit by a perturbed spherically symmetric BH spacetime, the Kodama and Ishibashi approach can be useful to study the GW emission in these processes. The relevance of this approach therefore extends well beyond the study of spherically symmetric solutions. For applications of this tool to the wave extraction of NR simulations see for instance [797].

In the Kodama and Ishibashi approach, the *D*-dimensional spacetime metric is assumed to have the form ${g_{\mu \nu}} = g_{\mu \nu}^{(0)} + {h_{\mu \nu}}$ where $g_{\mu \nu}^{(0)}$ is the Tangherlini solution and *h*_*μν*_ represents a small perturbation. Decomposing the *D*-dimensional spherical coordinates into ${x^\mu} = (t,r,\vec \phi)$ with *D* − 2 angular coordinates $\vec \phi = {\{{\phi ^a}\} _{a = 1, \ldots D - 2}}$, the perturbation *h*_*μν*_ can be expanded in spherical harmonics, as in the four-dimensional case (see Section 5.2.1). However, the expansion in *D* > 4 is more complex than its four-dimensional counterpart: there are three classes of perturbations called the “scalar”, “vector” and “tensor” perturbations. The former two classes correspond, in *D* = 4, to polar and axial perturbations, respectively. These perturbations are decomposed into scalar $({\mathcal S^{ll{\prime} \ldots}})$, vector $(\mathcal V_a^{ll{\prime} \ldots})$ and tensor $(\mathcal T_{ab}^{ll{\prime} \ldots})$ harmonics on the (*D* − 2)-sphere *S*^*D−*2^ and their gradients, as follows:

21 $${h_{\mu \nu}}(t,r,\vec \phi) = \sum\limits_{ll{\prime} \ldots} {\int\nolimits_{- \infty}^{+ \infty} {{e^{- i\omega t}}\left[ {h_{\mu \nu}^{{\rm{S}},ll{\prime} \ldots}(\omega, r, \vec \phi) + h_{\mu \nu}^{{\rm{V}},ll{\prime} \ldots}(\omega, r, \vec \phi) + h_{\mu \nu}^{{\rm{T}},ll{\prime} \ldots}(\omega, r, \vec \phi)} \right]} \,\;{\rm{d}}\omega,}$$

where *ll′*… denote harmonic indices on *S*^*D−*2^ and the superscripts S,V,T refer to scalar, vector and tensor perturbations, respectively. Introducing early upper case Latin indices *A, B*, … = 0, 1 and *x*^*A*^ = (*t,r*), the metric perturbations can be written as

22 $$\begin{array}{*{20}c} {h_{\mu \nu}^{{\rm{S}},ll\prime \ldots}(\omega ,r,\vec \phi)\;{\rm{d}}{x^\mu}\;{\rm{d}}{x^\nu} = \quad \quad \quad \quad \quad \quad \quad \quad \quad \quad \quad \quad \quad \quad \quad} \\ {\left[ {f_{A\,B}^{{\rm{S}}\;ll\prime \ldots}(\omega ,r)\;{\rm{d}}{x^A}\;{\rm{d}}{x^B} + H_L^{{\rm{S}}\;ll\prime \ldots}(\omega ,r)\;{\Omega _{ab}}\;{\rm{d}}{\phi ^a}\;{\rm{d}}{\phi ^b}} \right]\;{{\mathcal S}^{ll\prime \ldots}}(\vec \phi)\quad \quad} \\ {+ f_A^{{\rm{S}}\;ll\prime \ldots}(\omega ,r)\;{\rm{d}}{x^A}{\mathcal S}_a^{ll\prime \ldots}(\vec \phi)\;{\rm{d}}{\phi ^a} + H_T^{{\rm{S}}\;ll\prime \ldots}(\omega ,r){\mathcal S}_{ab}^{ll\prime \ldots}(\vec \phi)\;{\rm{d}}{\phi ^a}\;{\rm{d}}{\phi ^b}\quad} \\ {h_{\mu \nu}^{{\rm{V}},ll\prime \ldots}(\omega ,r,\vec \phi)\;{\rm{d}}{x^\mu}\;{\rm{d}}{x^\nu} = \quad \quad \quad \quad \quad \quad \quad \quad \quad \quad \quad \quad \quad \quad \quad} \\ {\left[ {f_A^{{\rm{V}}\;ll\prime \ldots}(\omega ,r)\;{\rm{d}}{x^A}} \right]\;{\mathcal V}_a^{ll\prime \ldots}(\vec \phi)\;{\rm{d}}{\phi ^a} + H_T^{{\rm{V}}\;ll\prime \ldots}(\omega ,r){\mathcal V}_{ab}^{ll\prime \ldots}(\vec \phi)\;{\rm{d}}{\phi ^a}\;{\rm{d}}{\phi ^b}\;} \\ {h_{\mu \nu}^{{\rm{T}},ll\prime \ldots}(\omega ,r,\vec \phi)\;{\rm{d}}{x^\mu}\;{\rm{d}}{x^\nu} = H_T^{{\rm{T}}\;ll\prime \ldots}(\omega ,r){\mathcal T}_{ab}^{ll\prime \ldots}(\vec \phi)\;{\rm{d}}{\phi ^a}\;{\rm{d}}{\phi ^b},\quad \quad \quad \;} \\ \end{array}$$

where $f_{AB}^{S,ll{\prime} \ldots}(\omega, r),f_A^{S,ll{\prime} \ldots}(\omega, r), \ldots$ are the spacetime perturbation functions. In the above expressions, Ω_*ab*_ is the metric on *S*^*D*−2^, *S*_*a*_ = −*S*,_*a*_/*k*, *S*_*ab*_ = *S*_:*ab*_/*k*^2^ minus trace terms, where *κ*^2^ = *l*(*l* + *D* − 3) is the eigenvalue of the scalar harmonics, and the “:” denotes the covariant derivative on *S*^*D*−*2*^; the traceless ${\mathcal V_{ab}}$ is defined in a similar way.

A set of gauge-invariant variables and the so-called “master functions”, generalizations of the Regge-Wheeler and Zerilli functions, can be constructed out of the metric perturbation functions and satisfy wave-like differential equations analogous to Eq. (19). The GW amplitude and its energy and momentum fluxes can be expressed in terms of these master functions.

For illustration of this procedure, we consider here the special case of scalar perturbations. We define the gauge-invariant quantities

23 $$F = {H_L} + {1 \over {D - 2}}{H_T} + {1 \over r}{X_A}{\hat D^A}r,\quad {F_{AB}} = {f_{AB}} + {\hat D_B}{X_A} + {\hat D_A}{X_B},$$

where we have dropped harmonic indices,

24 $${X_A} \equiv {r \over k}\left({{f_A} + {r \over k}{{\hat D}_A}{H_T}} \right),$$

and ${\hat D_A}$ denotes the covariant derivative associated with (*t,r*) sub-sector of the background metric. A master function Φ can be conveniently defined in terms of its time derivative according to

25 $${\partial _t}\Phi = (D - 2){r^{{{D - 4} \over 2}}}{{- {F^r}_t + 2r{\partial _t}F} \over {{k^2} - D + 2 + {{(D - 2)(D - 1)} \over 2}{{R_{\rm{S}}^{D - 3}} \over {{r^{D - 3}}}}}}.$$

From the master function, we can calculate the GW energy flux

26 $${{{\rm{d}}{E_{\ell m}}} \over {{\rm{d}}t}} = {1 \over {32\pi}}{{D - 3} \over {D - 2}}{k^2}({k^2} - D + 2){({\partial _t}{\Phi _{\ell m}})^2}.$$

The total radiated energy is obtained from integration in time and summation over all multipoles

27 $$E = \sum\limits_{\ell = 2}^\infty {\sum\limits_{m = - \ell}^\ell {\int\nolimits_{- \infty}^\infty {{{{\rm{d}}{E_{\ell m}}} \over {{\rm{d}}t}}} \;{\rm{d}}t.}}$$

In summary, this approach can be used, in analogy with the Regge-Wheeler-Zerilli formalism in four dimensions, to determine the quasi-normal mode spectrum (see, e.g., the review [95] and references therein), to determine the gravitational-wave emission due to a test source [98, 94], or to evaluate the flux of GWs emitted by a dynamical spacetime which tends asymptotically to a perturbed Tangherlini solution [797].

The generalization of this setup to higher-dimensional rotating (Myers-Perry [565]) BHs is still an open issue, since the decoupling of the perturbation equations has so far only been obtained in specific cases and for a subset of the perturbations [564, 496, 481].

Spacetime perturbation theory has also been used to study other types of higher-dimensional objects as for example black strings. Gregory and Laflamme [367, 368] considered a very specific sector of the possible gravitational perturbations of these objects, whereas Kudoh [495] performed a complete analysis that builds on the Kodama-Ishibashi approach.

#### State of the art

- Astrophysical systems. Perturbation theory has been applied extensively to the modelling of BHs and compact stars, either without source terms, including in particular quasi-normal modes [487, 316, 95], or with point particle sources. Note that wave emission from extended matter distributions can be understood as interference of waves from point particles [400, 693, 615]. Equations for BH perturbations have been derived for Schwarzschild [641, 830], RN [831], Kerr [744] and slowly rotating Kerr-Newman BHs [601]. Equations for perturbations of stars have been derived for spherically symmetric [753, 518, 196] and slowly rotating stars [197, 482].
  Equations of BH perturbations with a point particle source have been studied as a tool to understand BH dynamics. This is a decades old topic, historically divided into investigations of circular and quasi-circular motion, and head-ons or scatters.
  *Circular and quasi-circular motion*. Gravitational radiation from point particles in circular geodesics was studied in Refs. [551, 252, 130] for non-rotating BHs and in Ref. [267] for rotating BHs. This problem was reconsidered and thoroughly analyzed by Poisson, Cutler and collaborators, and by Tagoshi, Sasaki and Nakamura in a series of elegant works, where contact was also made with the PN expansion (see the *Living Reviews* article [675] and references therein). The emission of radiation, together with the self-gravity of the objects implies that particles do not follow geodesics of the background spacetime. Inclusion of dissipative effects is usually done by balance-type arguments [445, 446, 733, 338] but it can also be properly accounted for by computing the self-force effects of the particle motion (see the *Living Reviews* article [623] and references therein). EM waves from particles in circular motion around BHs were studied in Refs. [252, 130, 129].
  *Head-on or finite impact parameter collisions: non-rotating BHs*. Seminal work by Davis et al. [250, 251] models the gravitational radiation from BH collisions by a point particle falling from rest at infinity into a Schwarzschild BH. This work has been generalized to include head-on collisions at non-relativistic velocities [660, 317, 524, 93], at exactly the speed of light [179, 93], and to non-head-on collisions at non-relativistic velocities [269, 93].
  The infall of multiple point particles has been explored in Ref. [96] with particular emphasis on resonant excitation of QNMs. Shapiro and collaborators have investigated the infall or collapse of extended matter distributions through superpositions of point particle waveforms [400, 693, 615].
  Electromagnetic radiation from high-energy collisions of charged particles with uncharged BHs was studied in Ref. [181] including a comparison with zero-frequency limit (ZFL) predictions. Gravitational and EM radiation generated in collisions of charged BHs has been considered in Refs. [459, 460].
  *Head-on or finite impact parameter collisions: rotating BHs*. Gravitational radiation from point particle collisions with Kerr BHs has been studied in Refs. [484, 483, 485, 486]. Suggestions that cosmic censorship might fail in high-energy collisions with near-extremal Kerr BHs, have recently inspired further scrutiny of these scenarios [71, 72] as well as the investigation of enhanced absorption effects in the ultra-relativistic regime [376].
  *Close Limit approximation*. The close limit approximation was first compared against nonlinear simulations of equal-mass, non-rotating BHs starting from rest [634]. It has since been generalized to unequal-mass [35] or even the point particle limit [524], rotating BHs [494] and boosted BHs at second-order in perturbation theory [577]. Recently the close limit approximation has also been applied to initial configurations constructed with PN methods [503].
- Beyond electrovacuum GR. The resurgence of scalar-tensor theories as a viable and important prototype of alternative theories of gravity, as well as the conjectured existence of a multitude of fundamental bosonic degrees of freedom, has revived interest in BH dynamics in the presence of fundamental fields. Radiation from collisions of scalar-charged particles with BHs was studied in Ref. [134]. Radiation from massive scalar fields around rotating BHs was studied in Ref. [165] and shown to lead to floating orbits. Similar effects do *not* occur for massless gravitons [464].
- Beyond four-dimensions and asymptotic flatness. The gauge/gravity duality and related frameworks highlight the importance of (A)dS and higher-dimensional background spacetimes. The formalism to handle gravitational perturbations of four-dimensional, spherically symmetric asymptotically (A)dS BHs has been developed in Ref. [176], whereas perturbations of rotating AdS BHs were recently tackled [192, 277, 276]. Gravitational perturbations of higher-dimensional BHs can be handled through the elegant approach by Kodama and Ishibashi [479, 480], generalized in Ref. [495] to include perturbations of black strings. Perturbations of higher-dimensional, rotating BHs can be expressed in terms of a single master variable only in few special cases [496]. The generic case has been handled by numerical methods in the linear regime [270, 395].
  Scalar radiation by particles around Schwarzschild-AdS BHs has been studied in Refs. [180, 178, 177]. We are not aware of any studies on gravitational or electromagnetic radiation emitted by particles in orbit about BHs in spacetimes with a cosmological constant.
  The quadrupole formula was generalized to higher-dimensional spacetimes in Ref. [170]. The first fully relativistic calculation of GWs generated by point particles falling from rest into a higher-dimensional asymptotically flat non-rotating BH was done in Ref. [98], and later generalized to arbitrary velocity in Ref. [94]. The mass multipoles induced by an external gravitational field (i.e., the “Love numbers”) to a higher-dimensional BH, have been determined in Ref. [488].
  The close limit approximation was extended to higher-dimensional, asymptotically flat, space-times in Refs. [822, 823].

### The zero-frequency limit

#### Astrophysical systems in general relativity

While conceptually simple, the spacetime perturbation approach does involve solving one or more second-order, non-homogeneous differential equations. A very simple and useful estimate of the energy spectrum and total radiated gravitational energy can be obtained by using what is known as the ZFL or instantaneous collision approach.

The technique was derived by Weinberg in 1964 [773, 774] from quantum arguments, but it is equivalent to a purely classical calculation [707]. The approach is a consequence of the identity

28 $${\left. {\overline {(\dot h)}} \right\vert _{\omega = 0}} = \underset{\omega \rightarrow 0}{\lim} \int\nolimits_{- \infty}^{+ \infty} {\dot h{e^{- i\omega t}}} {\rm{d}}t = h(t = + \infty) - h(t = - \infty),$$

for the Fourier transform $\overline {(\dot h)} (\omega)$ of the time derivative of any metric perturbation *h*(*t*) (we omitted unimportant constant overall factors in the definition of the transform). Thus, the low-frequency spectrum depends exclusively on the asymptotic state of the colliding particles which can be readily computed from their Coulomb gravitational fields. Because the energy spectrum is related to $\bar \dot h(\omega)$ via

29 $${{{{\rm{d}}^2}E} \over {{\rm{d}}\Omega \;{\rm{d}}\omega}} \propto {r^2}{\overline {\left({(\dot h)} \right)} ^2},$$

we immediately conclude that the energy spectrum at low-frequencies depends only on the asymptotic states [774, 14, 707, 93, 489, 513]. Furthermore, if the asymptotic states are an accurate description of the collision at all times, as for instance if the colliding particles are point-like, then one expects the ZFL to be an accurate description of the problem.

For the head-on collision of two equal-mass objects each with mass *M*_*γ*_/2, Lorentz factor *γ* and velocity *υ* in the center-of-mass frame, one finds the ZFL prediction [707, 513]

30 $${{{{\rm{d}}^2}E} \over {{\rm{d}}\omega \;{\rm{d}}\Omega}} = {{{M^2}{\gamma ^2}{v^4}} \over {4{\pi ^2}}}{{{{\sin}^4}\theta} \over {{{(1 - {v^2}{{\cos}^2}\theta)}^2}}}.$$

The particles collide head-on along the *z*-axis and we use standard spherical coordinates. The spectrum is flat, i.e., *ω*-independent, thus the total radiated energy is formally divergent. The approach neglects the details of the interaction and the internal structure of the colliding and final objects, and the price to pay is the absence of a lengthscale, and therefore the appearance of this divergence. The divergence can be cured by introducing a phenomenological cutoff in frequency. If the final object has typical size *R*, we expect a cutoff *ω*_cutoff_ ∼ 1/*R* to be a reasonable assumption. BHs have a more reasonable cutoff in frequency given by their lowest QNMs; because QNMs are defined within a multipole decomposition, one needs first to decompose the ZFL spectrum into multipoles (see Appendix B of Ref. [93] and Appendix B2 of Ref. [513]). Finally, one observes that the high-energy limit *υ* →1 yields isotropic emission; when translated to a multipole dependence, it means that the energy in each multipole scales as 1/*l*^2^ in this limit.

The ZFL has been applied in a variety of contexts, including electromagnetism where it can be used to compute the electromagnetic radiation given away in *β*-decay [181, 455]; Wheeler used the ZFL to estimate the emission of gravitational and electromagnetic radiation from impulsive events [777]; the original treatment by Smarr considered only head-on collisions and computed only the spectrum and total emitted energy. These results have been generalized to include collisions with finite impact parameter and to a computation of the radiated momentum as well [513, 93]. Finally, recent nonlinear simulations of high-energy BH or star collisions yield impressive agreement with ZFL predictions [719, 93, 288, 134].

#### State of the art

- Astrophysical systems. The zero-frequency limit for head-on collisions of particles was used by Smarr [707] to understand gravitational radiation from BH collisions and in Ref. [14] to understand radiation from supernovae-like phenomena. It was later generalized to the nontrivial finite impact parameter case [513], and compared extensively with fully nonlinear numerical simulations [93]. Ref. [181] reports on collisions of an electromagnetic charge with a non-rotating BH in a spacetime perturbation approach and compares the results with a ZFL calculation.
- Beyond four-dimensional, electrovacuum GR. Recent work has started applying the ZFL to other spacetimes and theories. Brito [134] used the ZFL to understand head-on collisions of *scalar* charges with four-dimensional BHs. The ZFL has been extended to higher dimensions in Refs. [170, 513] and recently to specific AdS soliton spacetimes in Ref. [173].

### Shock wave collisions

An alternative technique to model the dynamics of collisons of two particles (or two BHs) at high energies describes the particles as gravitational shock waves. This method yields a bound on the emitted gravitational radiation using an exact solution, and provides an estimate of the radiation using a perturbative method. In the following we shall review both.

In *D* = 4 vacuum GR, a point-like particle is described by the Schwarzschild metric of mass *M*. The gravitational field of a particle moving with velocity *υ* is then obtained by boosting the Schwarzschild metric. Of particular interest is the limiting case where the velocity approaches the speed of light *υ* → *c*. Taking simultaneously the limit *M* →0 so that the zeroth component of the 4-momentum, *E*, is held fixed, $E = M/\sqrt {1 - {v^2}/{c^2}}$, one observes an infinite Lorentz contraction of the curvature in the spatial direction of the motion. In this limit, the geometry becomes that of an *impulsive or shock* gravitational *pp*-wave, i.e., a plane-fronted gravitational wave with parallel rays, sourced by a null particle. This is the Aichelburg-Sexl geometry [16] for which the curvature has support only on a null plane. In Brinkmann coordinates, the line element is:

31 $${\rm{d}}{s^2} = - {\rm{d}}u\;{\rm{d}}v + \kappa \Phi (\rho)\delta (u)\;{\rm{d}}{u^2} + {\rm{d}}{\rho ^2}{\rm{+}}{\rho ^2}{\rm{d}}{\phi ^2},\quad - \Delta [\kappa \Phi (\rho)] = 4\pi \kappa \delta (\rho){.}$$

Here the shock wave is moving in the positive *z*-direction, where (*u* = *t*−*z*, *υ* = *t*+*z*). This geometry solves the Einstein equations with energy momentum tensor *T*_*uu*_ = *Eδ*(*u*)*δ*(*ρ*) — corresponding to a null particle of energy *E* = *κ*/4*G*, traveling along *u* = 0 = *ρ* — provided the equation on the right-hand side of (31) is satisfied, where the Laplacian is in the flat 2-dimensional transverse space. Such a solution is given in closed analytic form by Φ(*ρ*) = − 2ln(*ρ*).

The usefulness of shock waves in modelling collisions of particles or BHs at very high energies relies on the following fact. Since the geometry of a single shock wave is flat outside a null plane, one can superimpose two shock wave solutions traveling in opposite directions and still obtain an *exact* solution of the Einstein equations, valid up to the moment when the two shock waves collide. The explicit metric is obtained by superimposing two copies of (31), one with support at *u* = 0 and another one with support at *υ* = 0. But it is more convenient to write down the geometry in coordinates for which test particle trajectories vary continuously as they cross the shock. These are called Rosen coordinates, $(\bar u,\bar v,\bar \rho, \phi)$; their relation with Brinkmann coordinates can be found in [420] and the line element for the superposition becomes

32 $${\rm{d}}{s^2} = - {\rm{d}}\bar u\;{\rm{d}}\bar v + \left[ {{{\left({1 + {{\kappa \bar u\theta (\bar u)} \over 2}\Phi {\prime\prime}} \right)}^2} + {{\left({1 + {{\kappa \bar v\theta (\bar v)} \over 2}\Phi {\prime\prime}} \right)}^2} - 1} \right]{\rm{d}}{\bar \rho ^2}$$

33 $$+ {\bar \rho ^2}\left[ {{{\left({1 + {{\kappa \bar u\theta (\bar u)} \over {2\bar \rho}}\Phi {\prime}} \right)}^2} + {{\left({1 + {{\kappa \bar v\theta (\bar v)} \over {2\bar \rho}}\Phi {\prime}} \right)}^2} - 1} \right]{\rm{d}}{\phi ^2}.$$

This metric is a valid description of the spacetime with the two shock waves except in the future light-cone of the collision, which occurs at $\bar u = 0 = \bar v$. Remarkably, and despite not knowing anything about the future development of the collision, an AH can be found for this geometry within its region of validity, as first pointed out by Penrose. Its existence indicates that a BH forms and moreover its area provides a lower bound for the mass of the BH [766]. This AH is the union of two surfaces,

$$\{{{\mathcal S}_1},\;{\rm{on}}\;\bar u = 0\;{\rm{and}}\;\bar v = - {\psi _1}(\bar \rho) \leq 0\}, \quad {\rm{and}}\;\quad \{{{\mathcal S}_2},\;{\rm{on}}\;\bar v = 0\;{\rm{and}}\;\bar u = - {\psi _2}(\bar \rho) \leq 0\},$$

for some functions *ψ*_1_,*ψ*_2_ to be determined. The relevant null normals to *S*_1_ and *S*_2_ are, respectively,

34 $${l_1} = {\partial _{\bar u}} - {1 \over 2}\psi _1^\prime{g^{\bar \rho \bar \rho}}{\partial _{\bar \rho}} + {1 \over 4}{(\psi _1^\prime)^2}{g^{\bar \rho \bar \rho}}{\partial _{\bar v}}\;,\quad \;{l_2} = {\partial _{\bar v}} - {1 \over 2}\psi _2^\prime{g^{\bar \rho \bar \rho}}{\partial _{\bar \rho}} + {1 \over 4}{(\psi _2^\prime)^2}{g^{\bar \rho \bar \rho}}{\partial _{\bar u}}.$$

One must then guarantee that these normals have zero expansion and are continuous at the intersection $\bar u = 0 = \bar v$. This yields the solution ${\psi _1}(\bar \rho) = \kappa \Phi (\bar \rho/\kappa) = {\psi _2}(\bar \rho)$. In particular, at the intersection, the AH has a polar radius $\bar \rho = \kappa$. The area of the AH is straightforwardly computed to be 2*π*^2^*κ*^2^, and provides a lower bound on the area of a section of the event horizon, and hence a lower bound on the mass of the BH: $M/\kappa > 1\sqrt 8$. By energy conservation, we then obtain an upper bound on the inelasticity *ϵ*, i.e., the fraction of the initial centre of mass energy which can be emitted in gravitational radiation:

35 $${\epsilon _{{\rm{AH}}}} \leq 1 - {1 \over {\sqrt 2}} \simeq 0{.}29{.}$$

Instead of providing a bound on the inelasticity, a more ambitious program is to determine the exact inelasticity by solving the Einstein equations in the future of the collision. Whereas an analytic exact solution seems out of reach, a numerical solution of the fully nonlinear field equations might be achievable, but none has been reported. The approach that has produced the most interesting results, so far, is to solve the Einstein equations perturbatively in the future of the collision.

To justify the use of a perturbative technique and introduce a perturbation expansion parameter, D’Eath and Payne [257, 258, 259] made the following argument. In a boosted frame, say in the negative *z* direction, one of the shock waves will become blueshifted whereas the other will become redshifted. These are, respectively, the waves with support on *u* = 0 and *υ* = 0. The geometry is still given by (33), but with the energy parameter *κ* multiplying *ū* terms (*ῡ* terms) replaced by a new energy parameter *ν* (parameter *λ*). For a large boost, *λ*/*ν* ≪ 1, or in other words, in the boosted frame there are a strong shock (at *u* = 0) and a weak shock (at *υ* = 0). The weak shock is regarded as a perturbation of the spacetime of the strong shock, and *λ*/*ν* provides the expansion parameter to study this perturbation. Moreover, to set up initial conditions for the post-collision perturbative expansion, one recasts the exact solution on the immediate future of the strong shock, *u* = 0^+^, in a perturbative form, even though it is an *exact* solution. It so happens that expressing the exact solution in such perturbative fashion only has terms up to second order:

36 $${g_{\mu \nu}}{\vert _{u = {0^ +}}} = {\nu ^2}\left[ {{\eta _{\mu \nu}} + {\lambda \over \nu}h_{\mu \nu}^{(1)} + {{\left({{\lambda \over \nu}} \right)}^2}h_{\mu \nu}^{(2)}} \right].$$

This perturbative expansion is performed in dimensionless coordinates of Brinkmann type, as in Eq. (31), since the latter are more intuitive than Rosen coordinates. The geometry to the future of the strong shock, on the other hand, will be of the form

37 $${g_{\mu \nu}}{\vert _{u > 0}} = {\nu ^2}\left[ {{\eta _{\mu \nu}} + \sum\limits_{i = 1}^\infty {{{\left({{\lambda \over \nu}} \right)}^i}} h_{\mu \nu}^{(i)}} \right],$$

where each of the $h_{\mu \nu}^{(i)}$ will be obtained by solving the Einstein equations to the necessary order. For instance, to obtain $h_{\mu \nu}^{(1)}$ one solves the linearized Einstein equations. In the de Donder gauge these yield a set of decoupled wave equations of the form $\square \bar h_{\mu \nu}^{(1)} = 0$, where the $\bar h_{\mu \nu}^{(1)}$ is the trace reversed metric perturbation. The wave equation must then be subjected to the boundary conditions (36). At higher orders, the problem can also be reduced to solving wave equations for $h_{\mu \nu}^{(i)}$ but now with sources provided by the perturbations of lower order [221].

After obtaining the metric perturbations to a given order, one must still compute the emitted gravitational radiation, in order to obtain the inelasticity. In the original work [256, 257, 258, 259], the metric perturbations were computed to second order and the gravitational radiation was extracted using Bondi’s formalism and the Bondi mass loss formula. The first-order results can equivalently be obtained using the Landau-Lifshitz pseudo-tensor for GW extraction [420]. The results in first and second order are, respectively:

38 $${\epsilon ^{(1)}} = 0.25,\quad {\epsilon ^{(2)}} = 0{.}164{.}$$

Let us close this subsection with three remarks on these results. Firstly, the results (38) are below the AH bound (35), as they should. Secondly, and as we shall see in Section 7.6, the second-order result is in excellent agreement with results from NR simulations. Finally, as we comment in the next subsection, the generalisation to higher dimensions of the first-order result reveals a remarkably simple pattern.

#### State of the art

The technique of superimposing two Aichelburg-Sexl shock waves [16] was first used by Penrose in unpublished work but quoted, for instance, in Ref. [257]. Penrose showed the existence of an AH for the case of a head-on collision, thus suggesting BH formation. Computing the area of the AH yields an upper bound on the fraction of the overall energy radiated away in GWs, i.e., the inelasticity. In the early 2000s, the method of superimposing shock waves and finding an AH was generalized to *D* ≥ 5 and non-zero impact parameter in Refs. [286, 818] and refined in Ref. [819] providing, in addition to a measure of the inelasticity, an estimate of the cross section for BH formation in a high-energy particle collision. A potential improvement to the AH based estimates was carried out in a series of papers by D’Eath and Payne [256, 257, 258, 259]. They computed the metric in the future of the collision perturbatively to second order in the head-on case. This method was generalized to *D* ≥ 5 in first-order perturbation theory [420, 222] yielding a very simple result: *ϵ*^(1)^ = 1/2 − 1/*D*. A formalism for higher order and the caveats of the method in the presence of electric charge were exhibited in [221]. AH formation in shock wave collisions with generalized profiles and asymptotics has been studied in [19, 739, 31, 282].

## Numerical Relativity

Generating time-dependent solutions to the Einstein equations using numerical methods involves an extended list of ingredients which can be loosely summarized as follows.

- Cast the field equations as an IBVP.
- Choose a specific formulation that admits a *well-posed* IBVP, i.e., there exist suitable choices for the following ingredients that ensure well posedness.
- Choose numerically suitable coordinate or *gauge* conditions.
- Discretize the resulting set of equations.
- Handle singularities such that they do not result in the generation of *non-assigned numbers* which rapidly swamp the computational domain.
- Construct initial data that solve the Einstein constraint equations and represent a realistic snapshot of the physical system under consideration.
- Specify suitable outer boundary conditions.
- Fix technical aspects: mesh refinement and/or multi-domains as well as use of multiple computer processors through parallelization.
- Apply diagnostic tools that measure GWs, BH horizons, momenta and masses, and other fields.

In this section, we will discuss state-of-the-art choices for these ingredients.

### Formulations of the Einstein equations

#### The ADM equations

The Einstein equations in *D* dimensions describing a spacetime with cosmological constant Λ and energy-matter content *T*_*αβ*_ are given by

39 $${R_{\alpha \beta}} - {1 \over 2}R{g_{\alpha \beta}} + \Lambda {g_{\alpha \beta}} = 8\pi {T_{\alpha \beta}}\quad \Leftrightarrow \quad {R_{\alpha \beta}} = 8\pi \left({{T_{\alpha \beta}} - {1 \over {D - 2}}{g_{\alpha \beta}}T} \right) + {2 \over {D - 2}}\Lambda {g_{\alpha \beta}}.$$

Elegant though this tensorial form of the equations is from a mathematical point of view, it is not immediately suitable for a numerical implementation. For one thing, the character of the equations as a *hyperbolic, parabolic* or *elliptic* system is not evident. In other words, are we dealing with an *initial-value* or a *boundary-value problem*? In fact, the Einstein equations are of mixed character in this regard and represent an IBVP. Well-posedness of the IBVP then requires a suitable formulation of the evolution equations, boundary conditions and initial data. We shall discuss this particular aspect in more detail further below, but first consider the general structure of the equations. The multitude of possible ways of writing the Einstein equations are commonly referred to as *formulations* of the equations and a good starting point for their discussion is the canonical “3 + 1” or “(*D* − 1) + 1” split originally developed by Arnowitt, Deser & Misner [47] and later reformulated by York [810, 812].

The tensorial form of the Einstein equations (39) fully reflects the unified viewpoint of space and time; it is only through the Lorentzian signature (−, +, …, +) of the metric that the timelike character of one of the coordinates manifests itself.^9^ It turns out crucial for understanding the character of Einstein’s equations to make the distinction between spacelike and timelike coordinates more explicit.

Let us consider for this purpose a spacetime described by a manifold *ℳ* equipped with a metric *g*_*αβ*_ of Lorentzian signature. We shall further assume that there exists a *foliation* of the spacetime in the sense that there exists a scalar function t : *ℳ* →ℝ with the following properties. (i) The 1-form **d***t* associated with the function *t* is timelike everywhere; (ii) The hypersurfaces Σ_*t*_ defined by *t* = const are non-intersecting and ∪_*t∈ℝ*_Σ_*t*_ = *ℳ*. Points inside each hypersurface Σ_*t*_ are labelled by spatial coordinates *x*^*I*^, *I* = 1, …, *D* − 1, and we refer to the coordinate system (*t, x*^*I*^) as *adapted* to the spacetime split.

**Figure 2:**
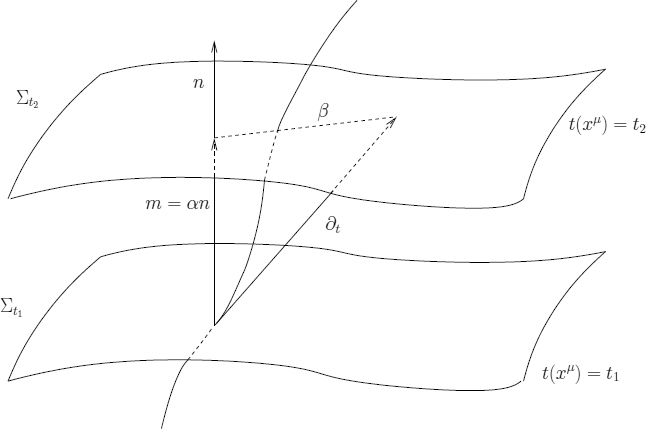
Illustration of two hypersurf aces of a foliation Σ_*t*_. Lapse *α* and shift *β*^*μ*^ are defined by the relation of the timelike unit normal field *n*^*μ*^ and the basis vector ***∂***_*t*_ associated with the coordinate *t*. Note that 〈**d***t*, *αn*〉 = 1 and, hence, the shift vector *β* is tangent to Σ_*t*_.

Next, we define the *lapse function α* and *shift vector β* through

40 $$\alpha \equiv {1 \over {\vert \vert {\bf{d}}t\vert \vert}},\quad \;\;{\beta ^\mu} \equiv {({\partial _t})^\mu} - \alpha {n^\mu},$$

where ***n*** ≡ − *α***d***t* is the timelike unit normal field. The geometrical interpretation of these quantities in terms of the timelike unit normal field *n*^*α*^ and the coordinate basis vector *∂*_*t*_ is illustrated in Figure 2. Using the relation 〈**d**_*t*_, **∂**_*t*_〉 = 1 and the definition of *α* and ***β***, one directly finds 〈**d***t*, ***β***〉 = 0, so that the shift ***β*** is tangent to the hypersurfaces Σ_*t*_. It measures the deviation of the coordinate vector ***∂***_*t*_ from the normal direction ***n***. The lapse function relates the proper time measured by an observer moving with four velocity *n*^*α*^ to the coordinate time *t*: Δ*τ* = *α*Δ*t*.

A key ingredient for the spacetime split of the equations is the projection of tensors onto time and space directions. For this purpose, the space projection operator is defined as ${\bot ^\alpha}_\mu \equiv {\delta ^\alpha}_\mu + {n^\alpha}{n_\mu}$. For a generic tensor ${T^{{\alpha _1}{\alpha _2} \ldots}}_{{\beta _1}{\beta _2} \ldots}$, its spatial projection is given by projecting each index speparately

41 $${(\bot T)^{{\alpha _1}{\alpha _2} \ldots}}_{{\beta _1}{\beta _2} \ldots} \equiv {\bot ^{{\alpha _1}}}_{{\mu _1}}{\bot ^{{\alpha _2}}}_{{\mu _2}} \ldots {\bot ^{{\nu _1}}}_{{\beta _1}}{\bot ^{{\nu _2}}}_{{\beta _2}} \ldots {T^{{\mu _1}{\mu _2} \ldots}}_{{\nu _1}{\nu _2} \ldots}.$$

A tensor ***S*** is called *tangent* to Σ_*t*_ if it is invariant under projection, i.e., ⊥***S*** = ***S***. In adapted coordinates, we can ignore the time components of such spatial tensors and it is common practice to denote their components with Latin indices *I, J*, … = 1, …, (*D* − 1). We similarly obtain time projections of a tensor by contracting its indices with *n*^*α*^. Mixed projections are obtained by contracting any combination of tensor indices with *n*^*α*^ and projecting the remaining ones with ${\bot ^\alpha}_\mu$. A particularly important tensor is obtained from the spatial projection of the spacetime metric

42 $${\gamma _{\alpha \beta}} \equiv {(\bot g)_{\alpha \beta}} = {\bot ^\mu}_\alpha {\bot ^\nu}_\beta {g_{\mu \nu}} = ({\delta ^\mu}_\alpha + {n^\mu}{n_\alpha})({\delta ^\nu}_\beta + {n^\nu}{n_\beta}){g_{\mu \nu}} = {g_{\alpha \beta}} + {n_\alpha}{n_\beta} = {\bot _{\alpha \beta}}.$$

*γ*_*αβ*_ is known as the *first fundamental form* or *spatial metric* and describes the intrinsic geometry of the spatial hypersurfaces Σ_*t*_. As we see from Eq. (42), it is identical to the projection operator. In the remainder, we will use both the ⊥ and *γ* symbols to denote this tensor depending on whether the emphasis is on the projection or the hypersurface geometry.

With our definitions, it is straightforward to show that the spacetime metric in adapted coordinates (*t, x*^*I*^) can be written as *ds*^2^ = −*α*^2^ d*t*^2^ + *γ*_*IJ*_(d*x*^*I*^ + *β*^*I*^ d*t*)(d*x*^*J*^ + *β*^*J*^ d*t*) or, equivalently, 43 It can be shown [364] that the spatial metric *γ*_*IJ*_ defines a unique, torsion-free and metric-compatible connection $\Gamma _{JK}^I = {1 \over 2}{\gamma ^{IM}}({\partial _J}{\gamma _{KM}} + {\partial _K}{\gamma _{MJ}} - {\partial _M}{\gamma _{JK}})$ whose covariant derivative for an arbitrary spatial tensor is given by

44 $${D_\gamma}{S^{{\alpha _1}{\alpha _2} \ldots}}_{{\beta _1}{\beta _2} \ldots} = {\bot ^\lambda}_\gamma {\bot ^{{\alpha _1}}}_{{\mu _1}}{\bot ^{{\alpha _2}}}_{{\mu _2}} \ldots {\bot ^{{\nu _1}}}_{{\beta _1}}{\bot ^{{\nu _2}}}_{{\beta _2}} \ldots {\nabla _\lambda}{S^{{\mu _1}{\mu _2} \ldots}}_{{\nu _1}{\nu _2} \ldots}.$$

The final ingredient required for the spacetime split of the Einstein equations is the *extrinsic curvature* or *second fundamental form* defined as

45 $${K_{\alpha \beta}} \equiv - \bot {\nabla _\beta}{n_\alpha}.$$

The sign convention employed here is common in NR but the “−” is sometimes omitted in other studies of GR. The definition (45) provides an intuitive geometric interpretation of the extrinsic curvature as the change in direction of the timelike unit normal field ***n*** as we move across the hypersurface Σ_*t*_. As indicated by its name, the extrinsic curvature thus describes the embedding of Σ_*t*_ inside the higher-dimensional spacetime manifold. The projection ⊥_*β*_*n*_*α*_ is symmetric under exchange of its indices in contrast to its non-projected counterpart ∇_*β*_*n*_*α*_. For the formulation of the Einstein equations in the spacetime split, it is helpful to introduce the vector field *m*^*μ*^ ≡ *αn*^*μ*^ = (*∂t*)^*μ*^ − *β*^*μ*^. A straightforward calculation shows that the extrinsic curvature can be expressed in terms of the Lie derivative of the spatial metric along either ***n*** or ***m*** according to

46 $${K_{\alpha \beta}} = - {1 \over 2}{{\mathcal L}_n}{\gamma _{\alpha \beta}} = - {1 \over {2\alpha}}{{\mathcal L}_m}{\gamma _{\alpha \beta}}.$$

We have now assembled all tools to calculate the spacetime projections of the Riemann tensor. In the following order, these are known as the Gauss, the contracted Gauss, the scalar Gauss, the Codazzi, the contracted Codazzi equation, as well as the final projection of the Riemann tensor and its contractions:

47 $$\begin{array}{*{20}c} {{\bot ^\mu}_\alpha {\bot ^\nu}_\beta {\bot ^\gamma}_\rho {\bot ^\sigma}_\delta {R^\rho}_{\sigma \mu \nu} = {{\mathcal R}^\gamma}_{\delta \alpha \beta} + {K^\gamma}_\alpha {K_{\delta \beta}} - {K^\gamma}_\beta {K_{\delta \alpha}},\quad \quad \quad \quad \quad \quad \quad \quad \quad \quad \quad \quad \quad \quad \quad \quad \quad} \\ {{\bot ^\mu}_\alpha {\bot ^\nu}_\beta {R_{\mu \nu}} + {\bot _{\mu \alpha}}{\bot ^\nu}_\beta {n^\rho}{n^\sigma}{R^\mu}_{\rho \nu \sigma} = {{\mathcal R}_{\alpha \beta}} + K{K_{\alpha \beta}} - {K^\mu}_\beta {K_{\alpha \mu}},\quad \quad \quad \quad \quad \quad \quad \quad \quad \quad \quad \quad \quad \,\quad \quad \quad \quad \quad \quad \quad \quad \quad \quad} \\ {R + 2{R_{\mu \nu}}{n^\mu}{n^\nu} = {\mathcal R} + {K^2} - {K^{\mu \nu}}{K_{\mu \nu}},\quad \quad \quad \quad \quad \quad \quad \quad \quad \quad \quad \quad \quad \,\,\quad \quad \quad} \\ {{\bot ^\gamma}_\rho {n^\sigma}{\bot ^\mu}_\alpha {\bot ^\nu}_\beta {R^\rho}_{\sigma \mu \nu} = {D_\beta}{K^\gamma}_\alpha - {D_\alpha}{K^\gamma}_\beta ,\,\quad \quad \quad \quad \quad \quad \quad \quad \quad \quad \quad \quad \quad \quad \quad \quad \quad \quad \quad \quad \;\;} \\ {{n^\sigma}{\bot ^\nu}_{\;\beta}{R_{\sigma \nu}} = {D_\beta}K - {D_\mu}{K^\mu}_\beta ,\,\quad \quad \quad \quad \quad \quad \quad \quad \quad \quad \quad \quad \quad \,\,\,\,\quad \quad \quad} \\ {{\bot _{\alpha \mu}}{\bot ^\nu}_\beta {n^\sigma}{n^\rho}{R^\mu}_{\rho \nu \sigma} = {1 \over \alpha}{{\mathcal L}_m}{K_{\alpha \beta}} + {K_{\alpha \mu}}{K^\mu}_\beta + {1 \over \alpha}{D_\alpha}{D_\beta}\alpha ,\,\quad \quad \quad \quad \quad \quad \quad \quad \quad \quad \quad \quad \quad} \\ {{\bot ^\mu}_\alpha {\bot ^\nu}_\beta {R_{\mu \nu}} = - {1 \over \alpha}{{\mathcal L}_m}{K_{\alpha \beta}} - 2{K_{\alpha \mu}}{K^\mu}_\beta - {1 \over \alpha}{D_\alpha}{D_\beta}\alpha + {{\mathcal R}_{\alpha \beta}} + K{K_{\alpha \beta}},\,\,\quad \quad \quad} \\ {R = - {2 \over \alpha}{{\mathcal L}_m}K - {2 \over \alpha}{\gamma ^{\mu \nu}}{D_\mu}{D_\nu}\alpha + {\mathcal R} + {K^2} + {K^{\mu \nu}}{K_{\mu \nu}}.\,\,} \\ \end{array}$$

Here, *ℛ* denotes the Riemann tensor and its contractions as defined in standard fashion from the spatial metric *γ*_*IJ*_. For simplicity, we have kept all spacetime indices here even for spatial tensors. As mentioned above, the time components can and will be discarded eventually.

By using Eq. (47), we can express the space and time projections of the Einstein equations (39) exclusively in terms of the first and second fundamental forms and their derivatives. It turns out helpful for this purpose to introduce the corresponding projections of the energy-momentum tensor which are given by

48 $$\rho = {T_{\mu \nu}}{n^\mu}{n^\nu},\quad \;{j_\alpha} = - {\bot ^\nu}_\alpha {T_{\mu \nu}}{n^\mu},$$

49 $${S_{\alpha \beta}} = {\bot ^\mu}_\alpha {\bot ^\nu}_\beta {T_{\mu \nu}},\quad S = {\gamma ^{\mu \nu}}{S_{\mu \nu}}.$$

then, the energy-momentum tensor is reconstructed according to *T*_*αβ*_ = *S*_*αβ*_+*n*_*α*_*j*_*β*_+*n*_*β*_*j*_*α*_+*ρn*_*α*_*n*_*β*_. Using the explicit expressions for the Lie derivatives

50 $${{\mathcal L}_m}{K_{IJ}} = {{\mathcal L}_{{\partial _t} - \beta}}{K_{IJ}} = {\partial _t}{K_{IJ}} - {\beta ^M}{\partial _M}{K_{IJ}} - {K_{MJ}}{\partial _I}{\beta ^M} - {K_{IM}}{\partial _J}{\beta ^M},$$

51 $${{\mathcal L}_m}{\gamma _{IJ}} = {{\mathcal L}_{{\partial _t} - \beta}}{\gamma _{IJ}} = {\partial _t}{\gamma _{IJ}} - {\beta ^M}{\partial _M}{\gamma _{IJ}} - {\gamma _{MJ}}{\partial _I}{\beta ^M} - {\gamma _{IM}}{\partial _J}{\beta ^M},$$

we obtain the spacetime split of the Einstein equations

52 $${\partial _t}{\gamma _{IJ}} = {\beta ^M}{\partial _M}{\gamma _{IJ}} + {\gamma _{MJ}}{\partial _I}{\beta ^M} + {\gamma _{IM}}{\partial _J}{\beta ^M} - 2\alpha {K_{IJ}},$$

53 $$\begin{array}{*{20}c} {{\partial _t}{K_{IJ}} = {\beta ^M}{\partial _M}{K_{IJ}} + {K_{MJ}}{\partial _I}{\beta ^M} + {K_{IM}}{\partial _J}{\beta ^M} - {D_I}{D_J}\alpha + \alpha ({{\mathcal R}_{IJ}} + K{K_{IJ}} - 2{K_{IM}}{K^M}_J)}\\ {+ 8\pi \alpha \left({{{S - \rho} \over {D - 2}}{\gamma _{IJ}} - {S_{IJ}}} \right) - {2 \over {D - 2}}\alpha \Lambda {\gamma _{IJ}},\quad \quad \quad \quad \quad \quad \quad \quad \quad \quad}\\ \end{array}$$

54 $$0 = {\mathcal R} + {K^2} - {K^{MN}}{K_{MN}} - 2\Lambda - 16\pi \rho,$$

55 $$0 = {D_I}K - {D_M}{K^M}_I + 8\pi {j_I}.$$

By virtue of the Bianchi identities, the constraints (54) and (55) are preserved under the evolution equations. Furthermore, we can see that *D*(*D* − 1)/2 second-order-in-time evolution equations for the *γ*_*IJ*_ are written as a first-order-in-time system through introduction of the extrinsic curvature. Additionally, we have obtained *D* constraint equations, the Hamiltonian and momentum constraints, which relate data within a hypersurface Σ_*t*_. We note that the Einstein equations do not determine the lapse *α* and shift *β*^*I*^. For the case of *D* = 4, these equations are often referred to as the ADM equations, although we note that Arnowitt, Deser & Misner used the canonical momentum in place of the extrinsic curvature in their original work [47]. Counting the degrees of freedom, we start with *D*(*D* + 1)/2 components of the spacetime metric. The Hamiltonian and momentum constraints determine *D* of these while *D* gauge functions represent the gauge freedom, leaving *D*(*D* − 3)/2 physical degrees of freedom as expected.

#### Well-posedness

The suitability of a given system of differential equations for a numerical time evolution critically depends on a continuous dependency of the solution on the initial data. This aspect is referred to as *well posedness* of the IBVP and is discussed in great detail in *Living Reviews* articles and other works [645, 674, 383, 427]. Here, we merely list the basic concepts and refer the interested reader to these articles.

Consider for simplicity an initial-value problem in one space and one time dimension for a single variable *u*(*t, x*) on an unbounded domain. Well-posedness requires a norm ∥ · ∥, i.e., a map from the space of functions *f*(*x*) to the real numbers ℝ, and a function *F*(*t*) independent of the initial data such that

56 $$\vert \vert \delta u(t, \cdot)\vert \vert \leq F(t)\vert \vert \delta u(0, \cdot)\vert \vert,$$

where *δu* denotes a linear perturbation relative to a solution *u*_0_(*t, x*) [380]. We note that *F*(*t*) may be a rapidly growing function, for example an exponential, so that well posedness represents a necessary but not sufficient criterion for suitability of a numerical scheme.

Well posedness of formulations of the Einstein equations is typically studied in terms of the *hyperbolicity* properties of the system in question. Hyperbolicity of a system of PDEs is often defined in terms of the *principal part*, that is, the terms of the PDE which contain the highest-order derivatives. We consider for simplicity a quasilinear first-order system for a set of variables ***u***(*t, x*)

57 $${\partial _t}u = P(t,x,u,{\partial _x})u.$$

The system is called strongly hyperbolic if *P* is a smooth differential operator and its associated principal symbol is symmetrizeable [567]. For the special case of constant coefficient systems this definition simplifies to the requirement that the principal symbol has only imaginary eigenvalues and a complete set of linearly independent eigenvectors. If linear independence of the eigenvectors is not satisfied, the system is called weakly hyperbolic. For more complex systems of equations, strong and weak hyperbolicity can be defined in a more general fashion [645, 567, 646, 674].

In our context, it is of particular importance that strong hyperbolicity is a necessary condition for a well posed IBVP [741, 742]. The ADM equations (52)–(53), in contrast, have been shown to be weakly but not strongly hyperbolic for fixed gauge [567]; likewise, a first-order reduction of the ADM equations has been shown to be weakly hyperbolic [468]. These results strongly indicate that the ADM formulation is not suitable for numerical evolutions of generic spacetimes.

A modification of the ADM equations which has been used with great success in NR is the BSSN system [78, 695] which is the subject of the next section.

#### The BSSN equations

It is interesting to note that the BSSN formulation had been developed in the 1990s before a comprehensive understanding of the hyperbolicity properties of the Einstein equations had been obtained; it was only about a decade after its first numerical application that strong hyperbolicity of the BSSN system [380] was demonstrated. We see here an example of how powerful a largely empirical approach can be in the derivation of successful numerical methods. And yet, our understanding of the mathematical properties is of more than academic interest as we shall see in Section 6.1.5 below when we discuss recent investigations of potential improvements of the BSSN system.

The modification of the ADM equations which results in the BSSN formulation consists of a trace split of the extrinsic curvature, a conformal decomposition of the spatial metric and of the traceless part of the extrinsic curvature and the introduction of the contracted Christoffel symbols as independent variables. For generality, we will again write the definitions of the variables and the equations for the case of an arbitrary number *D* of spacetime dimensions. We define

58 $$\begin{array}{*{20}c} {\chi = {\gamma ^{- 1/(D - 1)}},\quad \quad K = {\gamma ^{MN}}{K_{MN}},\quad \quad \quad}\\ {{{\tilde \gamma}_{IJ}} = \chi {\gamma _{IJ}}\quad \quad \quad \Leftrightarrow {{\tilde \gamma}^{IJ}} = {1 \over \chi}{\gamma ^{IJ}},\quad \quad \quad}\\ {{{\tilde A}_{IJ}} = \chi \left({{K_{IJ}} - {1 \over {D - 1}}{\gamma _{IJ}}K} \right)\quad \Leftrightarrow {K_{IJ}} = {1 \over \chi}\left({{{\tilde A}_{IJ}} + {1 \over {D - 1}}{{\tilde \gamma}_{IJ}}K} \right),\quad \quad}\\ {{{\tilde \Gamma}^I} = {{\tilde \gamma}^{MN}}\tilde \Gamma _{MN}^I,\quad \quad \quad \quad \quad \quad \quad \quad \quad \quad \quad \quad \quad \quad \quad}\\ \end{array}$$

where *γ* ≡ det*γ*_IJ_ and $\tilde \Gamma _{MN}^I$ is the Christoffel symbol defined in the usual manner in terms of the conformal metric ${\tilde \gamma _{IJ}}$. Note that the definition (58) implies two algebraic and one differential constraints

59 $$\tilde \gamma = 1,\quad {\tilde \gamma ^{MN}}{\tilde A_{MN}} = 0,\quad \;{{\mathcal G}^I} = {\tilde \Gamma ^I} - {\tilde \gamma ^{MN}}\tilde \Gamma _{MN}^I = 0.$$

Inserting the definition (58) into the ADM equations (52)–(53) and using the Hamiltonian and momentum constraints respectively in the evolution equations for *K* and ${\tilde \Gamma ^I}$ results in the BSSN evolution system

60 $${\partial _t}\chi = {\beta ^M}{\partial _M}\chi + {2 \over {D - 1}}\chi (\alpha K - {\partial _M}{\beta ^M}),$$

61 $${\partial _t}{\tilde \gamma _{IJ}} = {\beta ^M}{\partial _M}{\tilde \gamma _{IJ}} + 2{\tilde \gamma _{M(I}}{\partial _{J)}}{\beta ^M} - {2 \over {D - 1}}{\tilde \gamma _{IJ}}{\partial _M}{\beta ^M} - 2\alpha {\tilde A_{IJ}},$$

62 $$\begin{array}{*{20}c} {{\partial _t}K = {\beta ^M}{\partial _M}K - \chi {{\tilde \gamma}^{MN}}{D_M}{D_N}\alpha + \alpha {{\tilde A}^{MN}}{{\tilde A}_{MN}} + {1 \over {D - 1}}\alpha {K^2}}\\ {+ {{8\pi} \over {D - 2}}\alpha [S + (D - 3)\rho ] - {2 \over {D - 2}}\alpha \Lambda, \quad \quad \quad}\\ \end{array}$$

63 $$\begin{array}{*{20}c} {{\partial _t}{{\tilde A}_{IJ}} = {\beta ^M}{\partial _M}{{\tilde A}_{IJ}} + 2{{\tilde A}_{M(I}}{\partial _{J)}}{\beta ^M} - {2 \over {D - 1}}{{\tilde A}_{IJ}}{\partial _M}{\beta ^M} + \alpha K{{\tilde A}_{IJ}} - 2\alpha {{\tilde A}_{IM}}{{\tilde A}^M}_{\;\;\;\;J},}\\ {+ \chi {{(\alpha {{\mathcal R}_{IJ}} - {D_I}{D_J}\alpha - 8\pi \alpha {S_{IJ}})}^{{\rm{TF}}}},\quad \quad \quad \quad \quad \quad \quad \quad \quad \quad \quad}\\ \end{array}$$

64 $$\begin{array}{*{20}c} {{\partial _t}{{\tilde \Gamma}^I} = {\beta ^M}{\partial _M}{{\tilde \Gamma}^I} + {2 \over {D - 1}}{{\tilde \Gamma}^I}{\partial _M}{\beta ^M} - {{\tilde \Gamma}^M}{\partial _M}{\beta ^I} + {{\tilde \gamma}^{MN}}{\partial _M}{\partial _N}{\beta ^I} + {{D - 3} \over {D - 1}}{{\tilde \gamma}^{IM}}{\partial _M}{\partial _N}{\beta ^N}\quad \quad \quad \quad \quad \quad \quad \quad \quad \quad \quad \quad \quad \quad \quad}\\ {- {{\tilde A}^{IM}}\left[ {(D - 1)\alpha {{{\partial _M}\chi} \over \chi} + 2{\partial _M}\alpha} \right] + 2\alpha \tilde \Gamma _{MN}^I{{\tilde A}^{MN}} - 2{{D - 2} \over {D - 1}}\alpha {{\tilde \gamma}^{IM}}{\partial _M}K - 16\pi {\alpha \over \chi}{j^I}.\quad \quad \quad \quad \quad \quad \quad \quad \quad \quad \quad}\\ \end{array}$$

Here, the superscript “TF” denotes the trace-free part and we further use the following expressions that relate physical to conformal variables:

65 $$\Gamma _{JK}^I = \tilde \Gamma _{JK}^I - {1 \over {2\chi}}({\delta ^I}_K{\partial _J}\chi + {\delta ^I}_J{\partial _K}\chi - {\tilde \gamma _{JK}}{\tilde \gamma ^{IM}}{\partial _M}\chi),$$

66 $${{\mathcal R}_{IJ}} = {\tilde {\mathcal R}_{IJ}} + {\mathcal R}_{IJ}^\chi,$$

67 $${\mathcal R}_{IJ}^\chi = {{{{\tilde \gamma}_{IJ}}} \over {2\chi}}\left[ {{{\tilde \gamma}^{MN}}{{\tilde D}_M}{{\tilde D}_N}\chi - {{D - 1} \over {2\chi}}{{\tilde \gamma}^{MN}}{\partial _M}\chi \;{\partial _N}\chi} \right] + {{D - 3} \over {2\chi}}\left({{{\tilde D}_I}{{\tilde D}_J}\chi - {1 \over {2\chi}}{\partial _I}\chi \;{\partial _J}\chi} \right),$$

68 $${\tilde {\mathcal R}_{IJ}} = - {1 \over 2}{\tilde \gamma ^{MN}}{\partial _N}\;{\partial _N}{\tilde \gamma _{IJ}} + {\tilde \gamma _{M(I}}{\partial _{J)}}{\tilde \Gamma ^M} + {\tilde \Gamma ^M}{\tilde \Gamma _{(IJ)M}} + {\tilde \gamma ^{MN}}\left[ {2\tilde \Gamma _{M(I}^K{{\tilde \Gamma}_{J)KN}} + \tilde \Gamma _{IM}^K{{\tilde \Gamma}_{KJN}}} \right],$$

69 $${D_I}{D_J}\alpha = {\tilde D_I}{\tilde D_J}\alpha + {1 \over \chi}{\partial _{(I}}\chi {\partial _{J)}}\alpha - {1 \over {2\chi}}{\tilde \gamma _{IJ}}{\tilde \gamma ^{MN}}{\partial _M}\chi {\partial _N}\alpha.$$

In practical applications, it turns out necessary for numerical stability to enforce the algebraic constraint ${\tilde \gamma ^{MN}}{\tilde A_{MN}} = 0$ whereas enforcement of the unit determinant $\tilde \gamma = 1$ appears to be optional. A further subtlety is concerned with the presence of the conformal connection functions ${\tilde \Gamma ^I}$ on the right-hand side of the BSSN equations. Two recipes have been identified that provide long-term stable numerical evolutions. (i) The independently evolved ${\tilde \Gamma ^I}$ are only used when they appear in differentiated form but are replaced by their definition in terms of the conformal metric ${\tilde \gamma _{IJ}}$ everywhere else [23]. (ii) Alternatively, one can add to the right-hand side of Eq. (64) a term $- \sigma {\mathcal G^I}{\partial _M}{\beta ^M}$, where *σ* is a positive constant [803].

We finally note that in place of the variable χ, alternative choices for evolving the conformal factor are in use in some NR codes, namely *ϕ≡ −* (ln χ)/4 [65] or $W \equiv \sqrt \chi$ [540]. An overview of the specific choices of variables and treatment of the BSSN constraints for the present generation of codes is given in Section 4 of [429].

#### The generalized harmonic gauge formulation

It has been realized a long time ago that the Einstein equations have a mathematically appealing form if one imposes the *harmonic gauge* condition ${\square x^\alpha} = - {g^{\mu \nu}}\Gamma _{\mu \nu}^\alpha = 0$ [294]. Taking the derivative of this condition eliminates a specific combination of second derivatives from the Ricci tensor such that its principal part is that of the scalar wave operator

70 $${R_{\alpha \beta}} = - {1 \over 2}{g^{\mu \nu}}{\partial _\mu}{\partial _\nu}{g_{\alpha \beta}} + \ldots,$$

where the dots denote terms involving at most the first derivative of the metric. In consequence of this simplification of the principal part, the Einstein equations in harmonic gauge can straightforwardly be written as a strongly hyperbolic system. This formulation even satisfies the stronger condition of *symmetric hyperbolicity* which is defined in terms of the existence of a conserved, positive energy [674], and harmonic coordinates have played a key part in establishing local uniqueness of the solution to the Cauchy problem in GR [327, 141, 321].

This particularly appealing property of the Ricci tensor can be maintained for arbitrary coordinates by introducing the functions [333, 343]

71 $${H^\alpha} \equiv \square{x^\alpha} = - {g^{\mu \nu}}\Gamma _{\mu \nu}^\alpha,$$

and promoting them to the status of independently evolved variables; see also [630, 519]. This is called the *Generalized Harmonic Gauge formulation*.

With this definition, it turns out convenient to consider the generalized class of equations

72 $${R_{\alpha \beta}} - {\nabla _{(\alpha}}{{\mathcal C}_{\beta)}} = 8\pi \left({{T_{\alpha \beta}} - {1 \over {D - 2}}T{g_{\alpha \beta}}} \right) + {2 \over {D - 2}}\Lambda {g_{\alpha \beta}},$$

where ${\mathcal C^\alpha} \equiv {H^\alpha} - {\square x^\alpha}$. The addition of the term ${\nabla _{(\alpha}}{\mathcal C_{\beta)}}$ replaces the contribution of ${\nabla _{(\alpha}}{\square x_{\beta)}}$ to the Ricci tensor in terms of ${\nabla _{(\alpha}}{H_{\beta)}}$ and thus changes the principal part to that of the scalar wave operator. A solution to the Einstein equations is now obtained by solving Eq. (72) subject to the constraint ${\mathcal C_\alpha} = 0$.

The starting point for a Cauchy evolution are initial data *g*_*α*_*β* and *∂*_*t*_*g*_*α*_*β* which satisfy the constraints ${\mathcal C^\alpha} = 0 = {\partial _t}{\mathcal C^\alpha}$. A convenient manner to construct such initial data is to compute the initial *H*^*α*^ directly from Eq. (71) so that ${{\mathcal C}^\alpha} = 0$ by construction. It can then be shown [519] that the ADM constraints (54), (55) imply ${\partial _t}{\mathcal C^\mu} = 0$. By virtue of the contracted Bianchi identities, the evolution of the constraint system obeys the equation

73 $$\square{{\mathcal C}_\alpha} = - {{\mathcal C}^\mu}{\nabla _{(\mu}}{{\mathcal C}_{\alpha)}} - {{\mathcal C}^\mu}\left[ {8\pi \left({{T_{\mu \alpha}} - {1 \over {D - 2}}T{g_{\mu \alpha}}} \right) + {2 \over {D - 2}}\Lambda {g_{\mu \alpha}}} \right],$$

and the constraint ${{\mathcal C}^\alpha} = 0$ is preserved under time evolution in the continuum limit.

A key addition to the GHG formalism has been devised by Gundlach et al. [377] in the form of damping terms which prevent growth of numerical violations of the constraints ${{\mathcal C}^\alpha} = 0$ due to discretization or roundoff errors.

Including these damping terms and using the definition (71) to substitute higher derivatives in the Ricci tensor, the generalized Einstein equations (72) can be written as

74 $$\begin{array}{*{20}c} {{g^{\mu \nu}}{\partial _\mu}{\partial _\nu}{g_{\alpha \beta}} = - 2{\partial _\nu}{g_{\mu (\alpha}}\,{\partial _{\beta)}}{g^{\mu \nu}} - 2{\partial _{(\alpha}}{H_{\beta)}} + 2{H_\mu}\Gamma _{\alpha \beta}^\mu - 2\Gamma _{\nu \alpha}^\mu \Gamma _{\mu \beta}^\nu \quad \quad \quad \quad \quad \quad \quad} \\ {- 8\pi {T_{\alpha \beta}} + {{8\pi T - 2\Lambda} \over {D - 2}}{g_{\alpha \beta}} - 2\kappa \;[2{n_{(\alpha}}{{\mathcal C}_{\beta)}} - \lambda {g_{\alpha \beta}}{n^\mu}{{\mathcal C}_\mu}]\;\;,} \\ \end{array}$$

where *κ*, λ are user-specified constraint-damping parameters. An alternative first-order system of the GHG formulation has been presented in Ref. [519].

#### Beyond BSSN: Improvements for future applications

The vast majority of BH evolutions in generic 4-dimensional spacetimes have been performed with the GHG and the BSSN formulations. It is interesting to note in this context the complementary nature of the two formulations’ respective strengths and weaknesses. In particular, the constraint subsystem of the BSSN equations contains a zero-speed mode [100, 379, 378] which may lead to large Hamiltonian constraint violations. The GHG system does not contain such modes and furthermore admits a simple way of controlling constraint violations in the form of damping terms [377]. Finally, the wave-equation-type principal part of the GHG system allows for the straightforward construction of constraint-preserving boundary conditions [650, 492, 665]. On the other hand, the BSSN formulation is remarkably robust and allows for the simulation of BH binaries over a wide range of the parameter space with little if any modifications of the gauge conditions; cf. Section 6.4. Combination of these advantages in a single system has motivated the exploration of improvements to the BSSN system and in recent years resulted in the identification of a conformal version of the *Z*4 system, originally developed in Refs. [113, 112, 114], as a highly promising candidate [28, 163, 775, 428].

The key idea behind the *Z*4 system is to replace the Einstein equations with a generalized class of equations given by

75 $${G_{\alpha \beta}} = 8\pi {T_{\alpha \beta}} - {\nabla _\alpha}{Z_\beta} - {\nabla _\beta}{Z_\alpha} + {g_{\alpha \beta}}{\nabla _\mu}{Z^\mu} + {\kappa _1}[{n_\alpha}{Z_\beta} + {n_\beta}{Z_\alpha} + {\kappa _2}{g_{\alpha \beta}}{n_M}{Z^M}],$$

where *Z*_*α*_ is a vector field of constraints which is decomposed into space and time components according to $\Theta \equiv - {n^\mu}{Z_\mu}\;{\rm{and}}\;{Z_I} = \;{\bot ^\mu}_I{Z_\mu}$. Clearly, a solution to the Einstein equations is recovered provided the constraint *Z*_*μ*_ = 0 is satisfied. The conformal version of the *Z*4 system is obtained in the same manner as for the BSSN system and leads to time evolution equations for a set of variables nearly identical to the BSSN variables but augmented by the constraint variable Θ. The resulting evolution equations given in the literature vary in details, but clearly represent relatively minor modifications for existing BSSN codes [28, 163, 428]. Investigations have shown that the conformal *Z*4 system is indeed suitable for implementation of constraint preserving boundary conditions [664] and that constraint violations in simulations of gauge waves and BH and NS spacetimes are indeed smaller than those obtained for the BSSN system, in particular when constraint damping is actively enforced [28, 428]. This behaviour also manifests itself in more accurate results for the gravitational radiation in binary inspirals [428]. In summary, the conformal *Z*4 formulation is a very promising candidate for future numerical studies of BH spacetimes, including in particular the asymptotically AdS case where a rigorous control of the outer boundary is of utmost importance; cf. Section 6.6 below.

Another modification of the BSSN equations is based on the use of densitized versions of the trace of the extrinsic curvature and the lapse function as well as the traceless part of the extrinsic curvature with mixed indices [497, 795]. Some improvements in simulations of colliding BHs in higher-dimensional spacetimes have been found by careful exploration of the densitization parameter space [791].

#### Alternative formulations

The formulations discussed in the previous subsections are based on a spacetime split of the Einstein equations. A natural alternative to such a split is given by the characteristic approach pioneered by Bondi et al. and Sachs [118, 667]. Here, at least one coordinate is null and thus adapted to the characteristics of the vacuum Einstein equations. For generic four-dimensional spacetimes with no symmetry assumptions, the characteristic formalism results in a natural hierarchy of two evolution equations, four hypersurface equations relating variables on hypersurfaces of constant retarded (or advanced) time, as well as three supplementary and one trivial equations. A comprehensive overview of characteristic methods in NR is given in the *Living Reviews* article [788]. Although characteristic codes have been developed with great success in spacetimes with additional symmetry assumptions, evolutions of generic BH spacetimes face the problem of formation of caustics, resulting in a breakdown of the coordinate system; see [59] for a recent investigation. One possibility to avoid the problem of caustic formation is *Cauchy-characteristic matching*, the combination of a (*D* − 1) + 1 or Cauchy-type numerical scheme in the interior strong-field region with a characteristic scheme in the outer parts. In the form of Cauchy-characteristic extraction, i.e., ignoring the injection of information from the characteristic evolution into the inner Cauchy region, this approach has been used to extract GWs with high accuracy from numerical simulations of compact objects [642, 60].

**Figure 3:**
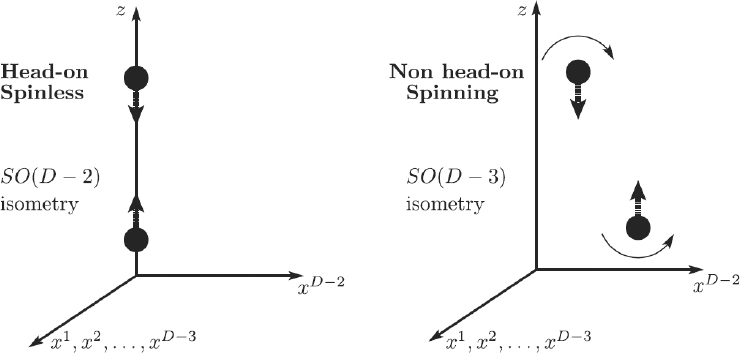
D-dimensional representation of head-on collisons for spinless BHs, with isometry group *SO*(*D*−2) (left), and non-head-on collisons for BHs spinning in the orbital plane, with isometry group *SO*(*D* − 3) (right). Image reproduced with permission from [841], copyright by APS.

**Figure 4:**
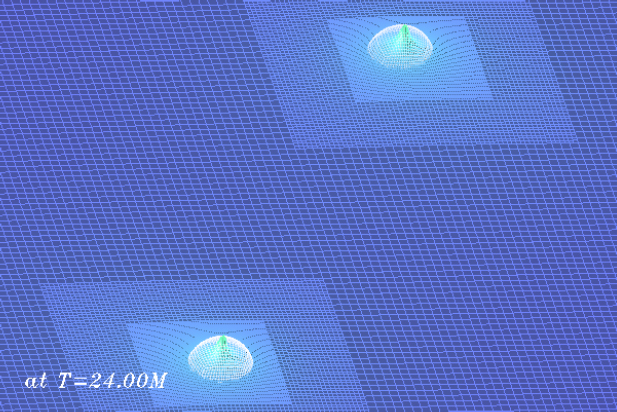
Illustration of mesh refinement for a BH binary with one spatial dimension suppressed. Around each BH (marked by the spherical AH), two nested boxes are visible. These are immersed within one large, common grid or *refinement level*.

All the Cauchy and characteristic or combined approaches we have discussed so far, evolve the physical spacetime, i.e., a manifold with metric (ℳ, *g*_*α*_*β*). An alternative approach for asymptotically flat spacetimes dating back to Hübner [444] instead considers the numerical construction of a conformal spacetime $(\tilde {\mathcal M},{\tilde g_{\alpha \beta}})$ where ${\tilde g_{\alpha \beta}} = {\Omega ^2}{g_{\alpha \beta}}$ subject to the condition that *g*_*α*_*β* satisfies the Einstein equations on ℳ. The conformal factor Ω vanishes at null infinity ℐ = ℐ^+^ ∪ ℐ− of the physical spacetime which is thus conformally related to an interior of the unphysical manifold $\tilde{\mathcal M},{\tilde g_{\alpha \beta}}$ which extends beyond the physical manifold. A version of these *conformal field equations* that overcomes the singular nature of the transformed Einstein equations at *ℐ* has been developed by Friedrich [332, 331]. This formulation is suitable for a 3+1 decomposition into a symmetric hyperbolic system^10^ of evolution equations for an enhanced (relative to the ADM decomposition) set of variables. The additional cost resulting from the larger set of variables, however, is mitigated by the fact that these include projections of the Weyl tensor that directly encode the GW content. Even though the conformal field equations have as yet not resulted in simulations of BH systems analogous to those achieved in BSSN or GHG, their elegance in handling the entire spacetime without truncation merits further investigation. For more details about the formulation and numerical applications, we refer the reader to the above articles, Lehner’s review [509], Frauendiener’s *Living Reviews* article [328] as well as [329, 26] and references therein. A brief historic overview of many formulations of the Einstein equations (including systems not discussed in this work) is given in Ref. [702]; see in particular Figures 3 and 4 therein.

We finally note that for simulations of spacetimes with high degrees of symmetry, it often turns out convenient to directly impose the symmetries on the shape of the line element rather than use one of the general formalisms discussed so far. As an example, we consider the classic study by May and White [544, 545] of the dynamics of spherically symmetric perfect fluid stars. A four-dimensional spherically symmetric spacetime can be described in terms of the simple line element

76 $${\rm{d}}{s^2} = - {a^2}(x,t)\;{\rm{d}}{t^2} + {b^2}(x,t)\;{\rm{d}}{x^2} + {R^2}(x,t)\;{\rm{d}}\Omega _2^2,$$

where ${\rm{d}}\Omega _2^2$ is the line element of the 2-sphere. May and White employ Lagrangian coordinates co-moving with the fluid shells which is imposed through the form of the energy-momentum tensor ${T^0}_0 = - \rho (1 + \epsilon),\;{T^1}_1 = {T^2}_2 = {T^3}_3 = P$. Here, the rest-mass density *ρ*, internal energy *ϵ*, and pressure *P* are functions of the radial and time coordinates. Plugging the line element (76) into the Einstein equations (39) with *D* = 4, Λ = 0 and the equations of conservation of energy-momentum ${\nabla _\mu}{T^\mu}_\alpha = 0$, result in a set of equations for the spatial and time derivatives of the metric and matter functions amenable for a numerical treatment; cf. Section II in Ref. [544] for details.

#### Einstein’s equations extended to include fundamental fields

The addition of matter to the spacetime can, in principle, be done using the formalism just laid down^11^. The simplest extension of the field equations to include matter is described by the Einstein-Hilbert action (in 4-dimensional asymptotically flat spacetimes) minimally coupled to a complex, massive scalar field Φ with mass parameter *μ*_*s*_ = *m*_*s*_/*ħ*,

77 $$S = \int {{{\rm{d}}^4}} x\sqrt {- g} \left({{R \over {16\pi}} - {1 \over 2}{g^{\mu \nu}}{\partial _\mu}{\Phi ^{\ast}}{\partial _\nu}\Phi - {1 \over 2}\mu _S^2{\Phi ^{\ast}}\Phi} \right).$$

if we introduce a time reduction variable defined as

78 $$\Pi = - {1 \over \alpha}({\partial _t} - {{\mathcal L}_\beta})\Phi,$$

we recover the equations of motion and constraints (52)–(55) with *D* = 4, Λ = 0 and with energy density *ρ*, energy-momentum flux *j*_*i*_ and spatial components *S*_*ij*_ of the energy-momentum tensor given by

79 $$\rho = {1 \over 2}\Pi ^{\ast}\Pi + {1 \over 2}\mu _S^2\Phi ^{\ast}\Phi + {1 \over 2}{D^i}\Phi ^{\ast}{D_i}\Phi,$$

80 $${j_i} = {1 \over 2}(\Pi ^{\ast}{D_i}\Phi + \Pi {D_i}\Phi ^{\ast}),$$

81 $${S_{ij}} = {1 \over 2}({D_i}\Phi ^{\ast}{D_j}\Phi + {D_i}\Phi {D_j}\Phi ^{\ast}) + {1 \over 2}{\gamma _{ij}}(\Pi ^{\ast}\Pi - \mu _S^2\Phi ^{\ast}\Phi - {D^k}\Phi ^{\ast}{D_k}\Phi).$$

Vector fields can be handled in similar fashion, we refer the reader to Ref. [794] for linear studies and to Refs. [595, 598, 838, 839] for full nonlinear evolutions.

In summary, a great deal of progress has been made in recent years concerning the well-posedness of the numerical methods used for the construction of spacetimes. We note, however, that the well-posedness of many problems beyond electrovacuum GR remains unknown at present. This includes, in particular, a wide class of alternative theories of gravity where it is not clear whether they admit well-posed IBVPs.

### Higher-dimensional NR in effective “3 + 1” form

Performing numerical simulations in generic higher-dimensional spacetimes represents a major challenge for simple computational reasons. Contemporary simulations of compact objects in four spacetime dimensions require ${\mathcal O(100)}$ cores and ${\mathcal O(100)}$ Gb of memory for storage of the fields on the computational domain. In the absence of spacetime symmetries, any extra spatial dimension needs to be resolved by ${\mathcal O(100)}$ grid points resulting in an increase by about two orders of magnitude in both memory requirement and computation time. In spite of the rapid advance in computer technology, present computational power is pushed to its limits with *D* = 5 or, at best, *D* = 6 spacetime dimensions. For these reasons, as well as the fact that the community already has robust codes available in *D* = 4 dimensions, NR applications to higher-dimensional spacetimes have so far focussed on symmetric spacetimes that allow for a reduction to an effectively four-dimensional formalism. Even though this implies a reduced class of spacetimes available for numerical study, many of the most important questions in higher-dimensional gravity actually fall into this class of spacetimes. In the following two subsections we will describe two different approaches to achieve such a dimensional reduction, for the cases of spacetimes with *SO*(*D* − 2) or *SO*(*D* − 3) isometry, i.e., the rotational symmetry leaving invariant *S*^*D*−3^. or *S*^*D*−4^, respectively (we denote with *S*^*n*^ the n-dimensional sphere). The group *SO*(*D* − 2) is the isometry of, for instance, head-on collisions of non-rotating BHs, while the group *SO*(*D* − 3) is the isometry of non-head-on collisions of non-rotating BHs; *SO*(*D* − 3) is also the isometry of non-head-on collisions of rotating BHs with one nonvanishing angular momentum, generating rotations on the orbital plane (see Figure 3). Furthermore, the *SO*(*D* − 3) group is the isometry of a single rotating BH, with one non-vanishing angular momentum. We remark that, in order to implement the higher-dimensional system in (modified) four-dimensional evolution codes, it is necessary to perform a 4 + (*D* − 4) splitting of the spacetime dimensions. With such splitting, the equations have a manifest *SO*(*D* − 3) symmetry, even when the actual isometry is larger.

We shall use the following conventions for indices. As before, Greek indices *a*, *β*, … cover all spacetime dimensions and late upper case capital Latin indices *I*, *J*, … = 1, … *D* − 1 cover the *D* − 1 spatial dimensions, whereas late lower case Latin indices *i*, *j*, … = 1, 2, 3 cover the three spatial dimensions of the eventual computational domain. In addition, we introduce barred Greek indices $\bar \alpha, \bar \beta, \ldots = 0, \ldots, 3$ which also include time, and early lower case Latin indices *a*, *b*, … = 4, …, *D* − 1 describing the *D* − 4 spatial directions associated with the rotational symmetry. Under the 4 + (*D* − 4) splitting of spacetime dimensions, then, the coordinates *x*^*μ*^ decompose as ${x^\mu} \rightarrow ({x^{\bar \mu}},{x^a})$. When explicitly stated, we shall consider instead a 3 + (*D* − 3) splitting, e.g., with barred Greek indices running from 0 to 2, and early lower case Latin indices running from 3 to *D* − 1.

#### Dimensional reduction by isometry

The idea of dimensional reduction had originally been developed by Geroch [347] for four-dimensional spacetimes possessing one Killing field as for example in the case of axisymmetry; for numerical applications see for example Refs. [535, 704, 722, 214]. The case of arbitrary spacetime dimensions and number of Killing vectors has been discussed in Refs. [210, 211].^12^ More recently, this idea has been used to develop a convenient formalism to perform NR simulations of BH dynamical systems in higher dimensions, with *SO*(*D* − 2) or *SO*(*D* − 3) isometry [841, 797]. Comprehensive summaries of this approach are given in Refs. [835, 791, 792].

The starting point is the general *D*-dimensional spacetime metric written in coordinates adapted to the symmetry

82 $${\rm{d}}{s^2} = {g_{\alpha \beta}}\;{\rm{d}}{x^\alpha}\;{\rm{d}}{x^\beta} = \left({{g_{\bar \mu \bar \nu}} + {e^2}{\kappa ^2}{g_{ab}}{B^a}_{\bar \mu}{B^b}_{\bar \nu}} \right)\;{\rm{d}}{x^{\bar \mu}}\;{\rm{d}}{x^{\bar \nu}} + 2e\kappa {B^a}_{\bar \mu}{g_{ab}}\;{\rm{d}}{x^{\bar \mu}}\;{\rm{d}}{x^b} + {g_{ab}}\;{\rm{d}}{x^a}\;{\rm{d}}{x^b}.$$

Here, *κ* and *e* represent a scale parameter and a coupling constant that will soon drop out and play no role in the eventual spacetime reduction. We note that the metric (82) is fully general in the same sense as the spacetime metric in the ADM split discussed in Section 6.1.1.

The special case of a *SO*(*D −* 2) (*SO*(*D −* 3)) isometry admits (*n*+1)*n*/2 Killing fields ***ξ***(*i*) where *n* ≡ *D* − 3 (*n ≡ D −* 4) stands for the number of extra dimensions. For *n* = 2, for instance, there exist three Killing fields given in spherical coordinates by ***ξ***(1) = **∂**_*ϕ*_, ***ξ***_(2)_ = sin *ϕ* ***∂***_*θ*_ +cot *θ* cos *ϕ****∂***_*ϕ*_, ***ξ***_(3)_ = cos *ϕ****∂***_*θ*_ − cot *θ* sin *ϕ* **∂**_*ϕ*_.

Killing’s equation *ℒ*_***ξ***__(*i*)_*gAB* = 0 implies that

83 $${{\mathcal L}_{{\xi _{(i)}}}}{g_{ab}} = 0,\quad \quad {{\mathcal L}_{{\xi _{(i)}}}}{B^a}_{\bar \mu} = 0,\quad \quad {{\mathcal L}_{{\xi _{(i)}}}}{g_{\bar \mu \bar \nu}} = 0,$$

where, as discussed above, the decomposition ${x^\mu} \rightarrow ({x^{\bar \mu}},{x^a})$ describes a 4 + (*D* − 4) splitting in the case of *SO*(*D* − 3) isometry, and a 3 + (*D* − 3) splitting in the case of *SO*(*D* − 2) isometry.

From these conditions, we draw the following conclusions: (i) ${g_{ab}} = {e^{2\psi ({x^{\bar \mu}})}}{\Omega _{ab}}$, where Ω_*ab*_ is the metric on the *S*^*n*^ sphere with unit radius and *ψ* is a free field; (ii) ${g_{\bar \mu \bar \nu}} = {g_{\bar \mu \bar \nu}}({x^{\bar \sigma}})$ in adapted coordinates; (iii) $[{\xi _{(i)}},{B_{\bar \mu}}] = 0$. We here remark an interesting consequence of the last property. Since, for *n* ≥ 2, there exist no nontrivial vector fields on *S*^*n*^ that commute with all Killing fields, all vector fields ${B^a}_{\bar \mu}$ vanish; when, instead, *n* = 0,1 (i.e., when *D* = 4, or *D* = 5 for *SO*(*D −* 3) isometry), this conclusion can not be made. In this approach, as it has been developed up to now [841, 797, 796], one restricts to the *n* ≥ 2 case, and it is then possible to assume ${B^a}_{\bar \mu} \equiv 0$. Eq. (82) then reduces to the form^13^

84 $${\rm{d}}{s^2} = {g_{\bar \mu \bar \nu}}\;{\rm{d}}{x^{\bar \mu}}\;{\rm{d}}{x^{\bar \nu}} + {e^{2\psi ({x^{\bar \mu}})}}\;{\Omega _{ab}}\;{\rm{d}}{x^a}\;{\rm{d}}{x^b}.$$

for this reason, this approach can only be applied when *D* ≥ 5 in the case of *SO*(*D −* 2) isometry, and *D* ≥ 6 in the case of *SO*(*D −* 3) isometry.

As mentioned above, since the Einstein equations have to be implemented in a four-dimensional NR code, we eventually have to perform a 4 + (*D −* 4) splitting, even when the spacetime isometry is *SO*(*D −* 2). This means that the line element is (84), with $\bar \alpha, \bar \beta, \ldots = 0, \ldots, 3$ and *a, b*, … = 4, …, *D* − 1. In this case, only a subset *SO*(*D* − 3) ⊂ *SO*(*D −* 2) of the isometry is manifest in the line element; the residual symmetry yields an extra relation among the components ${g_{\bar \mu \bar \nu}}$. If the isometry group is *SO*(*D −* 3), the line element is the same, but there is no extra relation.

A tedious but straightforward calculation [835] shows that the components of the *D*-dimensional Ricci tensor can then be written as

85 $$\begin{array}{*{20}c} {{R_{ab}} = \{(D - 5) - {e^{2\psi}}[(D - 4){\partial ^{\bar \mu}}\psi {\partial _{\bar \mu}}\psi + {{\bar \nabla}^{\bar \mu}}{\partial _{\bar \mu}}\psi ]\} {\Omega _{ab}},\quad \quad \quad \;}\\ {{R_{\bar \mu a}} = 0,\quad \quad \quad \quad \quad \quad \quad \quad \quad \quad \quad \quad \quad \quad \quad \quad \quad \quad \quad \quad \quad}\\ {{R_{\bar \mu \bar \nu}} = {{\bar R}_{\bar \mu \bar \nu}} - (D - 4)({{\bar \nabla}_{\bar \nu}}{\partial _{\bar \mu}}\psi + {\partial _{\bar \mu}}\psi {\partial _{\bar \nu}}\psi),\quad \quad \quad \quad \quad \quad \quad \quad \quad}\\ {R = \bar R + (D - 4)[(D - 5){e^{- 2\psi}} - 2{{\bar \nabla}^{\bar \mu}}{\partial _{\bar \mu}}\psi - (D - 3){\partial ^{\bar \mu}}\psi {\partial ^{\bar \mu}}\psi ],}\\ \end{array}$$

where ${\bar R_{\bar \mu \bar \nu}},\;\bar R\;{\rm{and}}\;\bar \nabla$ respectively denote the 3 + 1-dimensional Ricci tensor, Ricci scalar and covariant derivative associated with the 3 + 1 metric ${\bar g_{\bar \mu \bar \nu}} \equiv {g_{\bar \mu \bar \nu}}$. The *D*-dimensional vacuum Einstein equations with cosmological constant Λ can then be formulated in terms of fields on a 3 + 1-dimensional manifold

86 $${\bar R_{\bar \mu \bar \nu}} = (D - 4)({\bar \nabla _{\bar \nu}}{\partial _{\bar \mu}}\psi - {\partial _{\bar \mu}}\psi {\partial _{\bar \nu}}\psi) - \Lambda {\bar g_{\bar \mu \bar \nu}},$$

87 $${e^{2\psi}}[(D - 4){\partial ^{\bar \mu}}\psi {\partial _{\bar \mu}}\psi + {\bar \nabla ^{\bar \mu}}{\partial _{\bar \mu}}\psi - \Lambda ] = (D - 5).$$

One important comment is in order at this stage. If we describe the three spatial dimensions in terms of Cartesian coordinates (*x, y, z*), one of these is now a quasi-radial coordinate. Without loss of generality, we choose *y* and the computational domain is given by *x, z ∈* ℝ, *y* ≥ 0. In consequence of the radial nature of the *y* direction, *e*^2*ψ*^ = 0 at *y* = 0. Numerical problems arising from this coordinate singularity can be avoided by working instead with a rescaled version of the variable *e*^2*ψ*^. More specifically, we also include the BSSN conformal factor *e*−^4*ϕ*^ in the redefinition and write

88 $$\zeta \equiv {{{e^{- 4\phi}}} \over {{y^2}}}{e^{2\psi}}.$$

The BSSN version of the *D*-dimensional vacuum Einstein equations (86), (87) with Λ = 0 in its dimensionally reduced form on a 3 + 1 manifold is then given by Eqs. (60)–(64) with the following modifications, (i) Upper-case capital indices *I*, *J*, … are replaced with their lower case counterparts *i*, *j*, … = 1, 2, 3. (ii) The (*D* − 1) dimensional metric *γ*_*IJ*_, Christoffel symbols $\Gamma _{JK}^I$, covariant derivative *D*, conformal factor *χ* and extrinsic curvature variables *K* and *Ã*_*IJ*_ are replaced by the 3 dimensional metric *γ*_*ij*_, the 3 dimensional Christoffel symbols $\Gamma _{jk}^i$, the covariant derivative *D*, as well as the conformal factor χ, *K* and *A*_*ij*_ defined in analogy to Eq. (58) with *D* = 4, i.e.

89 $$\begin{array}{*{20}c} {\chi = {\gamma ^{- 1/3}},\quad \quad K = {\gamma ^{nm}}{K_{mn}},\quad \quad \quad \quad \quad \quad \quad \quad \quad}\\ {{{\tilde \gamma}_{ij}} = \chi {\gamma _{ij}}\quad \quad \quad \Leftrightarrow {{\tilde \gamma}^{ij}} = {1 \over \chi}{\gamma ^{ij}},\quad \quad \quad \quad \quad \quad}\\ {{{\tilde A}_{ij}} = \chi \left({{K_{ij}} - {1 \over 3}{\gamma _{ij}}K} \right)\quad \Leftrightarrow {K_{ij}} = {1 \over \chi}\left({{{\tilde A}_{ij}} + {1 \over 3}{{\tilde \gamma}_{ij}}K} \right),\quad \quad \quad \quad}\\ {{{\tilde \Gamma}^i} = {{\tilde \gamma}^{mn}}\tilde \Gamma _{mn}^i.\quad \quad \quad \quad \quad \quad \quad \quad \quad \quad \quad \quad \quad \quad \quad}\\ \end{array}$$

(iii) The extra dimensions manifest themselves as quasi-matter terms given by

90 $$\begin{array}{*{20}c} {{{4\pi (\rho + S)} \over {D - 4}} = (D - 5){\chi \over \zeta}{{{{\tilde \gamma}^{yy}}\zeta - 1} \over {{y^2}}} - {{2D - 7} \over {4\zeta}}{{\tilde \gamma}^{mn}}{\partial _m}\eta \;{\partial _n}\chi - \chi {{{{\tilde \Gamma}^y}} \over y} + {{D - 6} \over 4}{\chi \over {{\zeta ^2}}}{{\tilde \gamma}^{mn}}{\partial _m}\zeta \;{\partial _n}\zeta \quad \quad \quad \quad \quad \;\quad}\\ {+ {1 \over {2\zeta}}{{\tilde \gamma}^{mn}}(\chi {{\tilde D}_m}{\partial _n}\zeta - \zeta {{\tilde D}_m}{\partial _n}\chi) + (D - 4){{{{\tilde \gamma}^{ym}}} \over y}\left({{\chi \over \zeta}{\partial _m}\zeta - {\partial _m}\chi} \right) - {{K{K_\zeta}} \over \zeta} - {{{K^2}} \over 3}}\\ {- {1 \over 2}{{{{\tilde \gamma}^{ym}}} \over y}{\partial _m}\chi + {{D - 1} \over 4}{{\tilde \gamma}^{ym}}{{{\partial _m}\chi \;{\partial _n}\chi} \over \chi} - (D - 5){{\left({{{{K_\zeta}} \over \zeta} + {K \over 3}} \right)}^2},\quad \quad \quad \quad \quad \quad \;}\\ \end{array}$$

91 $$\begin{array}{*{20}c} {{{8\pi \chi S_{ij}^{{\rm{TF}}}} \over {D - 4}} = - \left({{{{K_\zeta}} \over \zeta} + {K \over 3}} \right){{\tilde A}_{ij}} + {1 \over 2}\left[ {{{2\chi} \over {y\zeta}}({\delta ^y}_{(j}{\partial _{i)}}\zeta - \zeta \tilde \Gamma _{ij}^y) + {1 \over {2\chi}}{\partial _i}\chi \;{\partial _j}\chi - {{\tilde D}_i}{\partial _j}\chi} \right. + {\chi \over \zeta}{{\tilde D}_i}{\partial _j}\zeta}\\ {{{\left. {+ {1 \over {2\chi}}{{\tilde \gamma}_{ij}}{{\tilde \gamma}^{mn}}{\partial _n}\chi \left({{\partial _m}\chi - {\chi \over \zeta}{\partial _m}\zeta} \right) - {{\tilde \gamma}_{ij}}{{{{\tilde \gamma}^{ym}}} \over y}{\partial _m}\chi - {\chi \over {2{\zeta ^2}}}{\partial _i}\zeta \;{\partial _j}\zeta} \right]}^{{\rm{TF}}}}}\\ \end{array}$$

92 $$\begin{array}{*{20}c} {{{16\pi {j_i}} \over {D - 4}} = {2 \over y}\left({{\delta ^y}_i{{{K_\zeta}} \over \zeta} - {{\tilde \gamma}^{ym}}{{\tilde A}_{mi}}} \right) + {2 \over \zeta}{\partial _i}{K_\zeta} - {{{K_\zeta}} \over \zeta}\left({{1 \over \chi}{\partial _i}\chi + {1 \over \zeta}{\partial _i}\zeta} \right) + {2 \over 3}{\partial _i}K}\\ {- {{\tilde \gamma}^{nm}}{{\tilde A}_{mi}}\left({{1 \over \zeta}{\partial _n}\zeta - {1 \over \chi}{\partial _n}\chi} \right).\quad \quad \quad \quad \quad \quad \quad \quad \quad \quad}\\ \end{array}$$

Here, *K*_*ζ*_ ≡ −(*2αy*^2^)^−1^(∂_*t*_ − *ℒβ*)(*ζy*^2^). The evolution of the field ζ is determined by Eq. (87) which in terms of the BSSN variables becomes

93 $${\partial _t}\zeta = {\beta ^m}{\partial _m}\zeta - 2\alpha {K_\zeta} - {2 \over 3}\zeta {\partial _m}{\beta ^m} + 2\zeta {{{\beta ^y}} \over y},$$

94 $$\begin{array}{*{20}c} {{\partial _t}{K_\zeta} = {\beta ^m}{\partial _m}{K_\zeta} - {2 \over 3}{K_\zeta}{\partial _m}{\beta ^m} + 2{{{\beta ^y}} \over y}{K_\zeta} - {1 \over 3}\zeta ({\partial _t} - {{\mathcal L}_\beta})K - \chi \zeta {{{{\tilde \gamma}^{ym}}} \over y}{\partial _m}\alpha \quad \quad \quad \quad \quad \quad \quad \quad \quad}\\ {- {1 \over 2}{{\tilde \gamma}^{ym}}{\partial _m}\alpha \;(\chi {\partial _n}\zeta - \zeta {\partial _n}\chi) + \alpha \left[ {(5 - D)\chi {{\zeta {{\tilde \gamma}^{yy}} - 1} \over {{y^2}}} + (4 - D)\chi {{{{\tilde \gamma}^{ym}}} \over y}{\partial _m}\zeta \quad \quad \quad \quad} \right.}\\ {+ {{2D - 7} \over 2}\zeta {{{{\tilde \gamma}^{ym}}} \over y}{\partial _m}\chi + {{6 - D} \over 4}{\chi \over \zeta}{{\tilde \gamma}^{mn}}{\partial _m}\zeta \;{\partial _n}\zeta + {{2D - 7} \over 4}{{\tilde \gamma}^{mn}}{\partial _m}\zeta \;{\partial _n}\chi \quad \quad \quad \quad \quad \quad}\\ {+ {{1 - D} \over 4}{\zeta \over \chi}{{\tilde \gamma}^{mn}}{\partial _m}\chi \;{\partial _n}\chi + (D - 6){{K_\zeta ^2} \over \zeta} + {{2D - 5} \over 3}K{K_\zeta} + {{D - 1} \over 9}\zeta {K^2}\quad \quad \quad \quad \quad \quad}\\ {\left. {+ {1 \over 2}{{\tilde \gamma}^{mn}}(\zeta {{\tilde D}_m}{\partial _n}\chi - \chi {{\tilde D}_m}{\partial _n}\zeta) + \chi \zeta {{{{\tilde \Gamma}^y}} \over y}} \right].\quad \quad \quad \quad \quad \quad \quad \quad \quad \quad \quad \quad \quad \quad \quad}\\ \end{array}$$

It has been demonstrated in Ref. [841] how all terms containing factors of *y* in the denominator can be regularized using the symmetry properties of tensors and their derivatives across *y* = 0 and assuming that the spacetime does not contain a conical singularity.

#### The cartoon method

The *cartoon method* has originally been developed in Ref. [25] for evolving axisymmetric four-dimensional spacetimes using an effectively two-dimensional spatial grid which employs ghostzones, i.e., a small number of extra gridpoints off the computational plane required for evaluating finite differences in the third spatial direction. Integration in time, however, is performed exclusively on the two-dimensional plane whereas the ghostzones are filled in after each timestep by appropriate interpolation of the fields in the plane and subsequent rotation of the solution using the axial spacetime symmetry. A version of this method has been applied to 5-dimensional spacetimes in Ref. [820]. For arbitrary spacetime dimensions, however, even the relatively small number of ghostzones required in every extra dimension leads to a substantial increase in the computational resources; for fourth-order finite differencing, for example, four ghostzones are required in each extra dimension resulting in an increase of the computational domain by an overall factor 5D−^4^. An elegant scheme to avoid this difficulty while preserving all advantages of the cartoon method has been developed in Ref. [630] and is sometimes referred to as the *modified cartoon method*. This method has been applied to *D* > 5 dimensions in Refs. [700, 512, 821] and we will discuss it now in more detail.

Let us consider for illustrating this method a *D*-dimensional spacetime with *SO*(*D* − 3) symmetry and Cartesian coordinates *x*^*μ*^ = (*t, x, y, z, w*^*a*^), where *a* = 4, …, *D* − 1. Without loss of generality, the coordinates are chosen such that the *SO*(*D* − 3) symmetry implies rotational symmetry in the planes spanned by each choice of two coordinates from^14^ (*y, w*^*a*^). The goal is to obtain a formulation of the D-dimensional Einstein equations (60)–(69) with *SO*(*D* − 3) symmetry that can be evolved exclusively on the *xyz* hyperplane. The tool employed for this purpose is to use the spacetime symmetries in order to trade derivatives off the hyperplane, i.e., in the *w*^*a*^ directions, for derivatives inside the hyperplane. Furthermore, the symmetry implies relations between the D-dimensional components of the BSSN variables.

These relations are obtained by applying a coordinate transformation from Cartesian to polar coordinates in any of the two-dimensional planes spanned by *y* and *w*, where *w* ≡ *w*^*a*^ for any particular choice of *a* ∈ {4, …, *D* − 1}

95 $$\begin{array}{*{20}c} {\rho = \sqrt {{y^2} + {w^2}}, y = \rho \cos \varphi,}\\ {\varphi = \arctan \;{w \over y},\;w = \rho \sin \varphi.}\\ \end{array}$$

Spherical symmetry in *n* ≡ *D* − 4 dimensions implies the existence of *n*(*n* + 1)/2 Killing vectors, one for each plane with rotational symmetry. For each Killing vector ***ξ***, the Lie derivative of the spacetime metric vanishes. For the *yw* plane, in particular, the Killing vector field is ***ξ*** = **∂**_ϕ_ and the Killing condition is given by the simple relation

96 $${\partial _\varphi}{g_{\mu \nu}} = 0.$$

All ADM and BSSN variables are constructed from the spacetime metric and a straightforward calculation demonstrates that the Lie derivatives along **∂**_ϕ_ of all these variables vanish. For *D ≥* 6, we can always choose the coordinates such that for *µ*, ≠ ϕ, *g*_*µϕ*_ = 0 which implies the vanishing of the BSSN variables ${\beta ^\varphi} = {\tilde \gamma ^{\mu \varphi}} = {\tilde \Gamma ^\varphi} = 0$ The case of *SO*(*D* − 3) symmetry in *D* = 5 dimensions is special in the same sense as already discussed in Section 6.2.1 and the vanishing of ${\tilde \Gamma ^\varphi}$ does not in general hold. As before, we therefore consider in *D* = 5 the more restricted class of *SO*(*D −* 2) isometry which implies ${\tilde \Gamma ^\varphi} = 0$. Finally, the Cartesian coordinates *w*^*a*^ can always be chosen such that the diagonal metric components are equal,

97 $${\gamma _{{w^1}{w^1}}} = {\gamma _{{w^2}{w^2}}} = \ldots \equiv {\gamma _{ww}}.$$

We can now exploit these properties in order to trade derivatives in the desired manner. We shall illustrate this for the second *w* derivative of the *ww* component of a symmetric $(_2^0)$ tensor density ***S*** of weight ${\mathcal W}$ which transforms under change of coordinates ${x^\mu} \leftrightarrow {x^{\hat \alpha}}$ according to

98 $${S_{\hat \alpha \hat \beta}} = {{\mathcal J}^{\mathcal W}}{{\partial {x^\mu}} \over {\partial {x^{\hat \alpha}}}}{{\partial {x^\nu}} \over {\partial {x^{\hat \beta}}}}{S_{\mu \nu}},\quad {\mathcal J} \equiv \det \left({{{\partial {x^\mu}} \over {\partial {x^{\hat \alpha}}}}} \right).$$

Specifically, we consider the coordinate transformation (95) where *ℑ = ρ*. In particular, this transformation implies

99 $${\partial _w}{S_{ww}} = {{\partial \rho} \over {\partial w}}{\partial _\rho}{S_{ww}} + {{\partial \varphi} \over {\partial w}}{\partial _\varphi}{S_{ww}},$$

and we can substitute

100 $${S_{ww}} = {{\mathcal J}^{- {\mathcal W}}}\left({{{\partial \rho} \over {\partial w}}{{\partial \rho} \over {\partial w}}{S_{\rho \rho}} + 2{{\partial \rho} \over {\partial w}}{{\partial \varphi} \over {\partial w}}{S_{\rho \varphi}} + {{\partial \varphi} \over {\partial w}}{{\partial \varphi} \over {\partial w}}{S_{\varphi \varphi}}} \right).$$

Inserting (100) into (99) and setting *S*_*ρϕ*_ = 0 yields a lengthy expression involving derivatives of *S*_*ρρ*_ and *S*_*ϕϕ*_ with respect to *ρ* and *ϕ*. The latter vanish due to symmetry and we substitute for the *ρ* derivatives using

101 $$\begin{array}{*{20}c} {{\partial _\rho}{S_{\rho \rho}} = \left({{{\partial y} \over {\partial \rho}}{\partial _y} + {{\partial w} \over {\partial \rho}}{\partial _w}} \right)\left[ {{{\mathcal J}^{\mathcal W}}\left({{{\partial y} \over {\partial \rho}}{{\partial y} \over {\partial \rho}}{S_{yy}} + 2{{\partial y} \over {\partial \rho}}{{\partial w} \over {\partial \rho}}{S_{yw}} + {{\partial w} \over {\partial \rho}}{{\partial w} \over {\partial \rho}}{S_{ww}}} \right)} \right],}\\ {{\partial _\rho}{S_{\varphi \varphi}} = \left({{{\partial y} \over {\partial \rho}}{\partial _y} + {{\partial w} \over {\partial \rho}}{\partial _w}} \right)\left[ {{{\mathcal J}^{\mathcal W}}\left({{{\partial y} \over {\partial \varphi}}{{\partial y} \over {\partial \varphi}}{S_{yy}} + 2{{\partial y} \over {\partial \varphi}}{{\partial w} \over {\partial \varphi}}{S_{yw}} + {{\partial w} \over {\partial \varphi}}{{\partial w} \over {\partial \varphi}}{S_{ww}}} \right)} \right].}\\ \end{array}$$

This gives a lengthy expression relating the *y* and *w* derivatives of *S*_*ww*_. Finally, we recall that we need these derivatives in the *xyz* hyperplane and therefore set *w* = 0. In order to obtain an expression for the second *w* derivative of *S*_*ww*_, we first differentiate the expression with respect to *w* and then set *w* = 0. The final result is given by

102 $${\partial _w}{S_{ww}} = 0,\quad \;{\partial _w}{\partial _w}{S_{ww}} = {{{\partial _y}{S_{ww}}} \over y} + 2{{{S_{yy}} - {S_{ww}}} \over {{y^2}}}.$$

Note that the density weight dropped out of this calculation, so that Eq. (102) is valid for the BSSN variables *Ã*_*μν*_ and ${\tilde \gamma _{\mu \nu}}$ as well.

Applying a similar procedure to all components of scalar, vector and symmetric tensor densities gives all expressions necessary to trade derivatives off the *xyz* hyperplane for those inside it. We summarize the expressions recalling our notation: a late Latin index, *i* = 1,…, 3 stands for either *x, y* or *z* whereas an early Latin index, *a* = 4,…, *D −* 1 represents any of the *w*^*a*^ directions. For scalar, vector and tensor fields Ψ, *V* and *T* we obtain

103 $$\begin{array}{*{20}c} {0 = {\partial _a}\Psi = {\partial _i}{\partial _a}\Psi = {V^a} = {\partial _i}{V^a} = {\partial _a}{\partial _b}{V^c} = {\partial _a}{V^i} = {\partial _a}{S_{bc}} = {\partial _i}{\partial _a}{S_{bc}} = {S_{ia}}} \\ {= {\partial _a}{\partial _b}{S_{ic}} = {\partial _a}{S_{ij}} = {\partial _i}{\partial _a}{S_{jk}},\quad \quad \quad \quad \quad \quad \quad \quad \quad \quad \quad \quad \quad \quad \quad \quad \;} \\ {{\partial _a}{\partial _b}\Psi = {\delta _{ab}}{{{\partial _y}\Psi} \over y},\;\;\,\quad \quad \quad \quad \quad \quad \quad \quad \quad \quad \quad \quad \quad \quad \quad \quad \quad \quad \quad \quad \quad \quad \quad \quad \quad \;} \\ {{\partial _a}{V^b} = {\delta ^b}_a{{{V^y}} \over y},\quad \;\;\,\quad \quad \quad \quad \quad \quad \quad \quad \quad \quad \quad \quad \quad \quad \quad \quad \quad \quad \quad \quad \quad \quad \quad \quad \;} \\ {{\partial _i}{\partial _a}{V^b} = {\delta ^b}_a\left({{{{\partial _i}{V^y}} \over y} - {\delta _{iy}}{{{V^y}} \over {{y^2}}}} \right)\;\;,\quad \quad \quad \quad \quad \quad \quad \quad \quad \quad \quad \quad \quad \quad \quad \quad \quad \quad \quad \quad \quad \;} \\ {{\partial _a}{\partial _b}{V^i} = {\delta _{ab}}\;\left({{{{\partial _y}{V^i}} \over y} - \delta _y^i{{{V^y}} \over {{y^2}}}} \right)\;\;,\quad \;\,\quad \quad \quad \quad \quad \quad \quad \quad \quad \quad \quad \quad \quad \quad \quad \quad \quad \quad \quad \quad} \\ {{S_{ab}} = {\delta _{ab}}{S_{ww}},\quad \;\quad \,\;\quad \quad \quad \quad \quad \quad \quad \quad \quad \quad \quad \quad \quad \quad \quad \quad \quad \quad \quad \quad \quad \quad \quad} \\ {{\partial _a}{\partial _b}{S_{cd}} = \left({{\delta _{ac}}{\delta _{bd}} + {\delta _{ad}}{\delta _{bc}}} \right){{{S_{yy}} - {S_{ww}}} \over {{y^2}}} + {\delta _{ab}}{\delta _{cd}}{{{\partial _y}{S_{ww}}} \over y},\;\;\;\;\quad \quad \quad \quad \quad \quad \quad \quad \quad \quad \quad \quad \,} \\ {{\partial _a}{S_{ib}} = {\delta _{ab}}{{{S_{iy}} - {\delta _{iy}}{S_{ww}}} \over y},\quad \quad \quad \quad \quad \quad \quad \quad \quad \quad \quad \quad \quad \quad \quad \quad \quad \quad \quad \quad \quad \quad \quad} \\ {{\partial _i}{\partial _a}{S_{jb}} = {\delta _{ab}}\left({{{{\partial _i}{S_{jy}} - {\delta _{jy}}{\partial _i}{S_{ww}}} \over y} - {\delta _{iy}}{{{S_{jy}} - {\delta _{jy}}{S_{ww}}} \over {{y^2}}}} \right)\;\;,\quad \quad \quad \quad \quad \quad \quad \quad \quad \quad \quad \;\quad \quad \;} \\ {{\partial _a}{\partial _b}{S_{ij}} = {\delta _{ab}}\left({{{{\partial _y}{S_{ij}}} \over y} - {{{\delta _{iy}}{S_{jy}} + {\delta _{iy}}{S_{iy}} - 2{\delta _{iy}}{\delta _{jy}}{S_{ww}}} \over {{y^2}}}} \right)\;\;.\quad \quad \quad \quad \quad \quad \quad \quad \quad \quad \quad \quad \;\quad} \\ \end{array}$$

By trading or eliminating derivatives using these relations, a numerical code can be written to evolve D-dimensional spacetimes with *SO*(*D −* 3) symmetry on a strictly three-dimensional computational grid. We finally note that y is a quasi-radial variable so that *y ≥* 0.

### Initial data

In Section 6.1 we have discussed different ways of casting the Einstein equations into a form suitable for numerical simulations. At the start of Section 6, we have listed a number of additional ingredients that need to be included for a complete numerical study and physical analysis of BH spacetimes. We will now discuss the main choices used in practical computations to address these remaining items, starting with the initial conditions.

As we have seen in Section 6.1, initial data to be used in time evolutions of the Einstein equations need to satisfy the Hamiltonian and momentum constraints (54), (55). A comprehensive overview of the approach to generate BH initial data is given by Cook’s *Living Reviews* article [224]. Here we merely summarize the key concepts used in the construction of vacuum initial data, but discuss in some more detail how solutions to the constraint equations can be generated in the presence of specific matter fields that play an important role in the applications discussed in Section 7.

One obvious way to obtain constraint-satisfying initial data is to directly use analytical solutions to the Einstein equations as for example the Schwarzschild solution in *D* = 4 in isotropic coordinates

104 $${\rm{d}}{s^2} = - {\left({{{M - 2r} \over {M + 2r}}} \right)^2}\;{\rm{d}}{t^2} + {\left({1 + {M \over {2r}}} \right)^4}[{\rm{d}}{r^2} + {r^2}({\rm{d}}{\theta ^2} + {\sin ^2}\theta \;{\rm{d}}{\phi ^2})].$$

Naturally, the numerical evolution of an analytically known spacetime solution does not generate new physical insight. It still serves as an important way to test numerical codes and, more importantly, analytically known solutions often form the starting point to construct generalized classes of initial data whose time evolution is not known without numerical study. Classic examples of such analytic initial data are the Misner [550] and Brill-Lindquist [133] solutions describing *n* non-spinning BHs at the moment of time symmetry. In Cartesian coordinates, the Brill-Lindquist data generalized to arbitrary *D* are given by

105 $${K_{IJ}} = 0,\quad {\gamma _{IJ}} = {\psi ^{4/(D - 3)}}{\delta _{IJ}},\quad \psi = 1 + \sum\limits_A {{{{\mu _A}} \over {4{{\left[ {\sum\nolimits_{K = 1}^{D - 1} {{{({x^K} - x_0^K)}^2}}} \right]}^{(D - 3)/2}}}},}$$

where the summations over *A* and *κ* run over the number of BHs and the spatial coordinates, respectively, and *μ*_*A*_ are parameters related to the mass of the *A-th* BH through the surface area Ω_*D*−2_ of the (*D −* 2)-dimensional sphere by *μ*_*A*_ = 16π*M*/[(*D* − 2)Ω_*D*−2_]. We remark that in the case of a single BH, the Brill-Lindquist initial data (105) reduce to the Schwarzschild spacetime in Cartesian, isotropic coordinates (see Eq. (137) in Section 6.7.1).

A systematic way to generate solutions to the constraints describing BHs in *D* = 4 dimensions is based on the York-Lichnerowicz split [515, 806, 807]. This split employs a conformal spatial metric defined by ${\gamma _i}_j = {\psi ^{4 -}}{\gamma _{ij}}$; note that in contrast to the BSSN variable ${\tilde \gamma _{ij}}$, in general det ${\bar \gamma _{ij}} \neq 1$. Applying a *conformal traceless split* to the extrinsic curvature according to

106 $${K_{ij}} = {A_{ij}} + {1 \over 3}{\gamma _{ij}}K,\quad {A^{ij}} = {\psi ^{- 10}}{\bar A^{ij}}\quad \Leftrightarrow \quad {A_{ij}} = {\psi ^{- 2}}{\bar A_{ij}},$$

and further decomposing *Ā*_*ij*_ into a longitudinal and a transverse traceless part, the momentum constraints simplify significantly; see [224] for details as well as a discussion of the alternative *physical transverse-traceless split* and *conformal thin-sandwich decomposition* [813]. The conformal thin-sandwich approach, in particular, provides a method to generate initial data for the lapse and shift which minimize the initial rate of change of the spatial metric, i.e., data in a quasi-equilibrium configuration [225, 190].

Under the further assumption of vanishing trace of the extrinsic curvature *K* = 0, a flat conformal metric ${\bar \gamma _{ij}} = {f_{ij}}$, where *f*_*ij*_ describes a flat Euclidean space, and asymptotic flatness lim_r→∞_ *ψ* = 1, the momentum constraint admits an analytic solution known as Bowen-York data [121]

107 $${\bar A_{ij}} = {3 \over {2{r^2}}}\left[ {{P_i}{n_j} + {P_j}{n_i} - ({f_{ij}} - {n_i}{n_j}){P^k}{n_k}} \right] + {3 \over {{r^3}}}\left({{\epsilon_{kil}}{S^l}{n^k}{n_j} + {\epsilon_{kjl}}{S^l}{n^k}{n_i}} \right),$$

with $r = \sqrt {{x^2} + {y^2} + {z^2}}, \;{n^i} = {x^i}/r$ the unit radial vector and user-specified parameters *P*^*i*^, *S*^*i*^. By calculating the momentum associated with the asymptotic translational and rotational Killing vectors $\xi _{(k)}^i$ [811], one can show that *P*^*i*^ and *S*^*i*^ represent the components of the total linear and angular momentum of the initial hypersurface. The linearity of the momentum constraint further allows us to superpose solutions $\bar A_{ij}^{(a)}$ of the type (107) and the total linear momentum is merely obtained by summing the individual $P_{(a)}^i$. The total angular momentum is given by the sum of the individual spins $S_{(a)}^i$ plus an additional contribution representing the orbital angular momentum. For the generalization of Misner data, it is necessary to construct inversion-symmetric solutions of the type (107) using the method of images [121, 224]. Such a procedure is not required for generalizing Brill-Lindquist data where a superposition of solutions $\bar A_{ij}^{(a)}$ of the type (107) can be used directly to calculate the extrinsic curvature from Eq. (106) and insert the resulting expressions into the vacuum Hamiltonian constraint given with the above listed simplifications by

108 $${\bar \nabla ^2}\psi + {1 \over 8}{K^{mn}}{K_{mn}}{\psi ^{- 7}} = 0,$$

where ${\bar \nabla ^2}$ is the Laplace operator associated with the flat metric *f*_*ij*_. This elliptic equation is commonly solved by decomposing *ψ* into a Brill-Lindquist piece ${\psi _{{\rm{BL}}}} = \sum\nolimits_{a = 1}^N {{m_a}/\vert \vec r - {{\vec r}_a}\vert}$ plus a regular piece *u = ψ − ψ*_BL_, where ${\vec r_a}$ denotes the location of the a-th BH and *m*_*a*_ a parameter that determines the BH mass and is sometimes referred to as the *bare mass*. Brandt & Brügmann [126] have proven existence and uniqueness of *C*^2^ regular solutions *u* to Eq. (108) and the resulting *puncture* data are the starting point of the majority of numerical BH evolutions using the BSSN moving puncture technique. The simplest example of this type of initial data is given by Schwarzschild’s solution in isotropic coordinates where

109 $${K_{mn}} = 0\,,\qquad \psi = 1 + {m \over {2r}}.$$

In particular, this solution admits the isometry *r* →*m*^2^ */*(4*r*) which leaves the coordinate sphere *r* = *m*/2 invariant, but maps the entire asymptotically flat spacetime *r* > m/2 into the interior and vice versa. The solution, therefore, consists of 2 asymptotically flat regions connected by a “throat” and spatial infinity of the far region is compactified into the single point *r* = 0 which is commonly referred to as the *puncture*. Originally, time evolutions of puncture initial data split the conformal factor, in analogy to the initial-data construction, into a singular Brill-Lindquist contribution given by the *ψ* in Eq. (109) plus a deviation *u* that is regular everywhere; cf. Section IV B in [24]. In this approach, the puncture locations remain fixed on the computational domain. The simulations through inspiral and merger by [159, 65], in contrast, evolve the entire conformal factor using gauge conditions that allow for the puncture to move across the domain and are, therefore, often referred to as “moving puncture evolutions”.

In spite of its popularity, there remain a few caveats with puncture data that have inspired explorations of alternative initial data. In particular, it has been shown that there exist no maximal, conformally flat spatial slices of the Kerr spacetime [341, 756]. Constructing puncture data of a single BH with non-zero Bowen-York parameter *S*^*i*^ will, therefore, inevitably result in a hyper-surface containing a BH plus some additional content which typically manifests itself in numerical evolutions as spurious GWs, colloquially referred to as “junk radiation”. For rotation parameters close to the limit of extremal Kerr BHs, the amount of spurious radiation rapidly increases leading to an upper limit of the dimensionless spin parameter *J/M*^2^ ≈ 0.93 for conformally flat Bowen-York-type data [226, 237, 238, 527]; BH initial data of Bowen-York type with a spin parameter above this value rapidly relax to rotating BHs with spin *χ* ≈ 0.93, probably through absorption of some fraction of the spurious radiation. This limit has been overcome [527, 528] by instead constructing initial data with an extended version of the conformal thin-sandwich method using superposed Kerr-Schild BHs [467]. In an alternative approach, most of the above outlined puncture method is applied but using a non-flat conformal metric; see for instance [493, 391].

In practice, puncture data are the method-of-choice for most evolutions performed with the BSSN-moving-puncture technique^15^ whereas GHG evolution schemes commonly start from conformal thin-sandwich data using either conformally flat or Kerr-Schild background data. Alternatively to both these approaches, initial data containing scalar fields which rapidly collapse to one or more BHs has also been employed [629].

The constraint equations in the presence of matter become more complex. A simple procedure can however be used to yield analytic solutions to the initial data problem in the presence of minimally coupled scalar fields [588, 586]. Although in general the constraints (54)–(55) have to be solved numerically, there is a large class of analytic or semi-analytic initial data for the Einstein equations extended to include scalar fields. The construction of constraint-satisfying initial data starts from a conformal transformation of the ADM variables [224]

110 $${\gamma _{ij}} = {\psi ^4}{\bar \gamma _{ij}},\quad \bar \gamma = \det {\bar \gamma _{ij}} = 1,$$

111 $${K_{ij}} = {A_{ij}} + {1 \over 3}{\gamma _{ij}}K,\quad {A_{ij}} = {\psi ^{- 2}}{\bar A_{ij}},$$

which can be used to re-write the constraints as

112 $${\mathcal H} = \bar \Delta \psi - {1 \over 8}\bar R\psi - {1 \over {12}}{K^2}{\psi ^5} + {1 \over 8}{\bar A^{ij}}{\bar A_{ij}}{\psi ^{- 7}} + \pi \psi [{\bar D^i}{\Phi ^{\ast}}{\bar D_i}\Phi + {\psi ^4}({\Pi ^{\ast}}\Pi + \mu _S^2{\Phi ^{\ast}}\Phi)],$$

113 $${{\mathcal M}_i} = {\bar D_j}\bar A_i^j - {2 \over 3}{\psi ^6}{\bar D_i}K - 4\pi {\psi ^6}({\Pi ^{\ast}}{\bar D_i}\Phi + \Pi {\bar D_i}{\Phi ^{\ast}}).$$

Here, $\bar \Delta = {\bar \gamma ^{ij}}{\bar D_i}{\bar D_j},\;\bar D$ and $\bar R$ denote the conformal covariant derivative and Ricci scalar and Π is a time reduction variable defined in (78).

Take for simplicity a single, non-rotating BH surrounded by a scalar field (more general cases are studied in Ref. [588, 586]). If we adopt the maximal slicing condition *K* = 0 and set *Ā*_*ij*_ = 0, Φ = 0, then the momentum constraint is immediately satisfied, and one is left with the the Hamiltonian constraint, which for conformal flatness, i.e., ${\bar \gamma _{ij}} = {f_{ij}}$ reads

114 $${\Delta _{{\rm{flat}}}}\psi = \left[ {{1 \over {{r^2}}}{\partial \over {\partial r}}{r^2}{\partial \over {\partial r}} + {1 \over {{r^2}\sin \theta}}{\partial \over {\partial \theta}}\sin \theta {\partial \over {\partial \theta}} + {1 \over {{r^2}{{\sin}^2}\theta}}{{{\partial ^2}} \over {\partial {\Phi ^2}}}} \right]\psi = - \pi {\psi ^5}\Pi {\Pi ^{\ast}}.$$

The ansatz

115 $$\Pi = {{{\psi ^{- 5/2}}} \over {\sqrt {r\pi}}}F(r)Z(\theta, \phi),$$

116 $$\psi = 1 + {M \over {2r}} + \sum\limits_{lm} {{{{u_{lm}}(r)} \over r}{Y_{lm}}(\theta, \phi),}$$

reduces the Hamiltonian constraint to

117 $$\sum\limits_{lm} {\left({u_{lm}^{\prime\prime} - {{l(l + 1)} \over {{r^2}}}{u_{lm}}} \right)\;} {Y_{lm}} = - F{(r)^2}Z{(\theta, \phi)^2}.$$

By a judicious choice of the angular function *Z*(*θ*, *ϕ*), or in other words, by projecting *Z*(*θ*, *ϕ*) onto spherical harmonics *Y*_*lm*_, the above equation reduces to a single second-order, ordinary differential equation. Thus, the complex problem of finding appropriate initial data for massive scalar fields was reduced to an almost trivial problem, which admits some interesting analytical solutions [588, 586]. Let us focus for defmiteness on spherically symmetric solutions (we refer the reader to Ref. [588, 586] for the general case), by taking a Gaussian-type solution ansatz,

118 $$Z(\theta, \phi) = {1 \over {\sqrt {4\pi}}}\,,\quad F(r) = {A_{00}} \times \sqrt r {e^{- {{{{{(r - {r_0})}^2}} \over {{w^2}}}}}},$$

where *A*_00_ is the scalar field amplitude and *r*_0_ and *w* are the location of the center of the Gaussian and its width. By solving Eq. (117), we obtain the only non-vanishing component of *u*_*lm*_(*r*)

119 $${u_{00}} = A_{00}^2{{w[{w^2} - 4{r_0}(r - {r_0})]} \over {16\sqrt 2}}\left[ {{\rm{erf}}\left({{{\sqrt 2 (r - {r_0})} \over w}} \right) - 1} \right] - A_{00}^2{{{r_0}{w^2}} \over {8\sqrt \pi}}{e^{- 2{{(r - {r_0})}^2}/{w^2}}}\,,$$

where we have imposed that *u*_*lm*_ → 0 at infinity. Other solutions can be obtained by adding a constant to (119).

### Gauge conditions

We have seen in Section 6.1, that the Einstein equations do not make any predictions about the gauge functions; the ADM equations leave lapse *α* and shift *β*^*i*^ unspecified and the GHG equations make no predictions about the source functions *H*^*α*^. Instead, these functions can be freely specified by the user and represent the coordinate or gauge-invariance of the theory of GR. Whereas the physical properties of a spacetime remain unchanged under gauge transformations, the performance of numerical evolution schemes depends sensitively on the gauge choice. It is well-known, for example, that evolutions of the Schwarzschild spacetime employing geodesic slicing *α* = 1 and vanishing shift *β*^*i*^ = 0 inevitably reach a hypersurface containing the BH singularity after a coordinate time interval *t = πM* [709]; computers respond to singular functions with non-assigned numbers which rapidly swamp the entire computational domain and render further evolution in time practically useless. This problem can be avoided by controlling the lapse function such that the evolution in proper time slows down in the vicinity of singular points in the spacetime [312]. Such slicing conditions are called *singularity avoiding* and have been studied systematically in the form of the Bona-Massó family of slicing conditions [116]; see also [343, 20]. A potential problem arising from the use of singularity avoiding slicing is the different progress in proper time in different regions of the computational domain resulting in a phenomenon often referred to as “grid stretching” or “slice stretching” which can be compensated with suitable non-zero choices for the shift vector [24].

The particular coordinate conditions used with great success in the BSSN-based moving puncture approach [159, 65] in *D* = 4 dimensions are variants of the “1+log” slicing and “Γ-driver” shift condition [24]

120 $${\partial _t}\alpha = {\beta ^m}{\partial _m}\alpha - 2\alpha K,$$

121 $${\partial _t}{\beta ^i} = {\beta ^m}{\partial _m}{\beta ^i} + {3 \over 4}{B^i},$$

122 $${\partial _t}{B^i} = {\beta ^m}{\partial _m}{B^i} + {\partial _t}{\tilde \Gamma ^i} - \eta {B^i}\,.$$

We note that the variable *B*^*i*^ introduced here is an auxiliary variable to write the second-order-in-time equation for the shift vector as a first-order system and has no relation with the variable of the same name introduced in Eq. (82). The “damping” factor *η* in Eq. (122) is specified either as a constant, a function depending on the coordinates *x*^*i*^ and BH parameters [683], a function of the BSSN variables [559, 560], or evolved as an independent variable [29]. A first-order-in-time evolution equation for *β*^*i*^ has been suggested in [758] which results from integration of Eqs. (121), (122)

123 $${\partial _t}{\beta ^i} = {\beta ^m}{\partial _m}{\beta ^i} + {3 \over 4}{\tilde \Gamma ^i} - \eta {\beta ^i}.$$

Some NR codes omit the advection derivatives of the form *β*^*m*^*∂*_*m*_ in Eqs. (120)–(123). Long-term stable numerical simulations of BHs in higher dimensions require modifications in the coefficients in Eqs. (120)–(123) [700] and/or the addition of extra terms [841]. Reference [313] recently suggested a modification of Eq. (120) for the lapse function *α* that significantly reduces noise generated by a sharp initial gauge wave pulse as it crosses mesh refinement boundaries.

BH simulations with the GHG formulation employ a wider range of coordinate conditions. For example, Pretorius’ breakthrough evolutions [629] set *Hi* = 0 and

124 $${\square H_t} = - {\xi _1}{{\alpha - 1} \over {{\alpha ^\eta}}} + {\xi _2}{n^\mu}{\partial _\mu}{H_t},$$

with parameters ξ1 = 19/m, ξ_2_ = 2.5/m, *η* = 5 where *m* denotes the mass of a single BH. An alternative choice used with great success in long binary BH inspiral simulations [735] sets *H*_*α*_ such that the dynamics are minimized at early stages of the evolution, gradually changes to harmonic gauge *H*_*α*_ = 0 during the binary inspiral and uses a damped harmonic gauge near merger

125 $${H_\alpha} = {\mu _0}{\left[ {\ln \left({{{\sqrt \gamma} \over \alpha}} \right)} \right]^2}\left[ {\ln \left({{{\sqrt \gamma} \over \alpha}} \right){n_\alpha} - {\alpha ^{- 1}}{g_{\alpha m}}{\beta ^m}} \right],$$

where *μ*_0_ is a free parameter. We note in this context that for *D* = 4, the GHG source functions *H*^*α*^ are related to the ADM lapse and shift functions through [630]

126 $${n^\mu}{H_\mu} = - K - {n^\mu}{\partial _\mu}\ln \alpha,$$

127 $${\gamma ^{\mu i}}{H_\mu} = - {\gamma ^{mn}}\Gamma _{mn}^i + {\gamma ^{im}}{\partial _m}\ln \alpha + {1 \over \alpha}{n^\mu}{\partial _\mu}{\beta ^i}.$$

### Discretization of the equations

In the previous sections, we have derived formulations of the Einstein equations in the form of an IBVP. Given an initial snapshot of the physical system under consideration, the evolution equations, as for example in the form of the BSSN equations (60)–(64), then predict the evolution of the system in time. These evolution equations take the form of a set of nonlinear partial differential equations which relate a number of grid variables and their time and spatial derivatives. Computers, on the other hand, exclusively operate with (large sets of) numbers and for a numerical simulation we need to translate the differential equations into expressions relating arrays of numbers.

The common methods to implement this *discretization* of the equations are *finite differencing*, the *finite element, finite volume* and *spectral* methods. Finite element and volume methods are popular choices in various computational applications, but have as yet not been applied to time evolutions of BH spacetimes. Spectral methods provide a particularly efficient and accurate approach for numerical modelling provided the functions do not develop discontinuities. Even though BH spacetimes contain singularities, the use of singularity excision provides a tool to remove these from the computational domain. This approach has been used with great success in the SpEC code to evolve inspiralling and merging BH binaries with very high accuracy; see, e.g., [122, 220, 526]. Spectral methods have also been used successfully for the modelling of spacetimes with high degrees of symmetry [205, 206, 207] and play an important role in the construction of initial data [39, 38, 836]. An indepth discussion of spectral methods is given in the *Living Reviews* article [365]. The main advantage of finite differencing methods is their comparative simplicity. Furthermore, they have proved very robust in the modelling of rather extreme BH configurations as for example BHs colliding near the speed of light [719, 587, 716] or binaries with mass ratios up to 1:100 [525, 523, 718].

**Mesh refinement and domain decomposition**: BH spacetimes often involve lengthscales that differ by orders of magnitude. The BH horizon extends over lengths of the order ${\mathcal O(1)}$ *M* where *M* is the mass of the BH. Inspiralling BH binaries, on the other hand, emit GWs with wavelengths of ${\mathcal O}({10^2})\;M$. Furthermore, GWs are rigorously defined only at infinity. In practice, wave extraction is often performed at finite radii but these need to be large enough to ensure that systematic errors are small. In order to accomodate accurate wave extraction, computational domains used for the modelling of asymptotically flat BH spacetimes typically have a size of ${\mathcal O}({10^3})\;M$. With present computational infrastructure it is not possible to evolve such large domains with a uniform, high resolution that is sufficient to accurately model the steep profiles arising near the BH horizon. The solution to this difficulty is the use of mesh refinement, i.e., a grid resolution that depends on the location in space and may also vary in time. The use of mesh refinement in BH modelling is simplified by the remarkably rigid nature of BHs which rarely exhibit complicated structure beyond some mild deformation of a sphere. The requirements of increased resolution are, therefore, simpler to implement than, say, in the modelling of airplanes or helicopters. In BH spacetimes the grid resolution must be highest near the BH horizon and it decreases gradually at larger and larger distances from the BH. In terms of the internal book-keeping, this allows for a particularly efficient manner to arrange regions of refinement which is often referred to as *moving boxes*. A set of nested boxes with outwardly decreasing resolution is centered on each BH of the spacetime and follows the BH motion. These sets of boxes are immersed in one or more common boxes which are large enough to accomodate those centered on the BHs. As the BHs approach each other, boxes originally centered on the BHs merge into one and become part of the common-box hierarchy. A snapshot of such moving boxes is displayed in Figure 4.

Mesh refinement in NR has been pioneered by Choptuik in his seminal study on critical phenomena in the collapse of scalar fields [212]. The first application of mesh refinement to the evolution of BH binaries was performed by Brügmann [140]. There exists a variety of mesh refinement packages available for use in NR including Bam [140], Had [384], Pamr/Amrd [754], Paramesh [534], Samrai [672] and the Carpet [684, 184] package integrated into the Cactus Computational Toolkit [155]. For additional information on Cactus see also the Einstein Toolkit webpage [300] and the lecture notes [840]. A particular mesh-refinement algorithm used for many BH applications is the Berger-Oliger [90] scheme where coarse and fine levels communicate through interpolation in the form of the *prolongation* and *restriction* operation; see [684] for details. Alternatively, the different lengthscales can be handled efficiently through the use of multiple domains of different shapes. Communication between the individual subdomains is performed either through overlaps or directly at the boundary for touching domains. Details of this domain decomposition can be found in [618, 146] and references therein.

### Boundary conditions

In NR, we typically encounter two types of physical boundaries, (i) inner boundaries due to the treatment of spacetime singularities in BH solutions and (ii) the outer boundary either at infinite distance from the strong-field sources or, in the form of an approximation to this scenario, at the outer edge of the computational domain at large but finite distances.

**Singularity excision**: BH spacetimes generically contain singularities, either physical singularities with a divergent Ricci scalar or coordinate singularities where the spacetime curvature is well behaved but some tensor components approach zero or inifinite values. In the case of the Schwarzschild solution in Schwarzschild coordinates, for example, *r* = 0 corresponds to a physical singularity whereas the singular behaviour of the metric components *g*_*tt*_ and *g*_*rr*_ at *r* = 2 *M* merely reflects the unsuitable nature of the coordinates as *r* →2 *M* and can be cured, for example, by transforming to Kruskal-Szekeres coordinates; cf. for example Chapter 7 in Ref. [186]. Both types of singularities give rise to trouble in the numerical modelling of spacetimes because computers only handle finite numbers. Some control is available in the form of gauge conditions as discussed in Section 6.4; the evolution of proper time is slowed down when the evolution gets close to a singularity. In general, however, BH singularities require some special numerical treatment.

**Figure 5:**
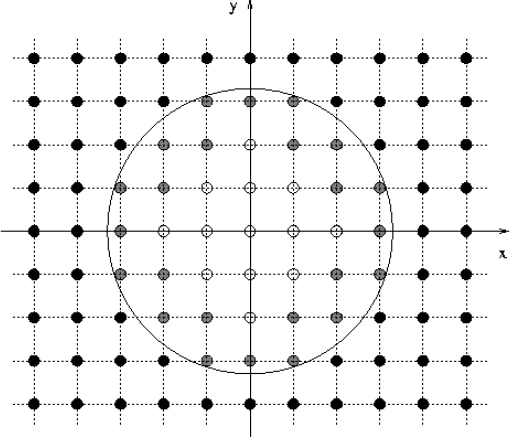
Illustration of singularity excision. The small circles represent vertices of a numerical grid on a two-dimensional cross section of the computational domain containing the spacetime singularity, in this case at the origin. A finite region around the singularity but within the event horizon (large circle) is excluded from the numerical evolution (white circles). Gray circles represent the excision boundary where function values can be obtained through regular evolution in time using sideways derivative operators as appropriate (e.g., [630]) or regular update with spectral methods (e.g., [677, 678]), or through extrapolation (e.g., [703, 723]). The regular evolution of exterior grid points (black circles) is performed with standard techniques using information also on the excision boundary.

Such a treatment is most commonly achieved in the form of *singularity* or *BH excision* originally suggested by Unruh as quoted in [746]. According to Penrose’s cosmic censorship conjecture, a spacetime singularity should be cloaked inside an event horizon and the spacetime region outside the event horizon is causally disconnected from the dynamics inside (see Section 3.2.1). The excision technique is based on the corresponding assumption that the numerical treatment of the spacetime inside the horizon has no causal effect on the exterior. In particular, excising a finite region around the singularity but within the horizon should leave the exterior spacetime unaffected. This is illustrated in Figure 5 where the excision region is represented by small white circles which are excluded from the numerical evolution. Regular grid points, represented in the figure by black circles, on the other hand are evolved normally. As we have seen in the previous section, the numerical evolution in time of functions at a particular grid point typically requires information from neighbouring grid points. The updating of variables at regular points, therefore, requires data on the excision boundary represented in Figure 5 by gray circles. Inside the BH horizon, represented by the large circle in the figure, however, information can only propagate inwards, so that the variables on the excision boundary can be obtained through use of sideways derivative operators (e.g., [630]), extrapolation (e.g., [703, 723]) or regular update with spectral methods (e.g., [677, 678]). Singularity excision has been used with great success in many numerical BH evolutions [23, 723, 721, 629, 678, 70, 418].

Quite remarkably, the moving puncture method for evolving BHs does not employ any such specific numerical treatment near BH singularities, but instead applies the same evolution procedure for points arbitrarily close to singularities as for points far away and appears “to get away with it”. In view of the remarkable success of the moving puncture method, various authors have explored the behaviour of the puncture singularity in the case of a single Schwarzschild BH [392, 136, 137, 390, 138, 264]. Initially, the puncture represents spatial infinity on the *other* side of the wormhole geometry compactified into a single point. Under numerical evolution using moving puncture gauge conditions, however, the region immediately around this singularity rapidly evolves into a so-called trumpet geometry which is partially covered by the numerical grid to an extent that depends on the numerical resolution; cf. Figure 1 in [138]. In practice, the singularity falls through the inevitably finite resolution of the computational grid which thus facilitates a *natural excision* of the spacetime singularity without the need of any special numerical treatment.

**Outer boundary**: Most physical scenarios of interest for NR involve spatial domains of infinite extent and there arises the question how these may be accomodated inside the finite memory of a computer system. Probably the most elegant and rigorous method is to apply a spatial compactification, i.e., a coordinate transformation that maps the entire domain including spatial infinity to a finite coordinate range. Such compactification is best achieved in characteristic formulations of the Einstein equations where the spacetime foliation in terms of ingoing and/or outgoing light cones may ensure adequate resolution of in- or outgoing radiation throughout the entire domain. In principle, such a compactification can also be implemented in Cauchy-type formulations, but here it typically leads to an increasing blueshift of radiative signals as they propagate towards spatial infinity. As a consequence, any discretization method applied will eventually fail to resolve the propagating features. This approach has been used in Pretorius’ breakthrough [629] and the effective damping of radiative signals at large distances through underresolving them approximates a no-ingoing-radiation boundary condition. An intriguing alternative consists in using instead a space-time slicing of *asymptotically* null hypersurfaces which play a key role in the conformal field equations [328]. To our knowledge, this method has not yet been applied successfully to BH simulations in either astrophysical problems or simulations of the type reviewed here, but may well merit more study in the future.

The vast majority of Cauchy-based NR applications instead resort to an approximative treatment where the infinite spatial domain is truncated and modeled as a compact domain with “suitable” outer boundary conditions. Ideally, the boundary conditions would satisfy the following requirements [651]: They (i) ensure well posedness of the IBVP, (ii) are compatible with the constraint equations, and (iii) correctly represent the physical conditions, which in almost all practical applications means that they control or minimize the ingoing gravitational radiation.

Boundary conditions meeting these requirements at least partially have been studied most extensively for the harmonic or generalized harmonic formulation of the Einstein equations [492, 651, 58, 652, 665].

For the BSSN system, such boundary conditions have as yet not been identified and practical applications commonly apply *outgoing radiation* or *Sommerfeld* boundary conditions, which are an approximation in this context, where they are applied at large but finite distances from the strong-field region. Let us assume, for this purpose, that a given grid variable *f* asymptotes to a constant background value *f*_0_ in the limit of large *r* and contains a leading order deviation *u*(*t* − *r*)/*r*^*n*^ from this value, where *u* remains finite as r →*∞*, and *n* is a constant positive integer number. For r →*∞*, we therefore have

128 $$f(t,r) = {f_0} + {{u(t - r)} \over {{r^n}}},$$

where the dependence on retarded time represents the outgoing nature of the radiative deviations. In consequence, *∂*_*t*_*u* + *∂*_*r*_*u* = 0, which translates into the following conditon for the grid variable *f*

129 $${\partial _t}f + n{{f - {f_0}} \over r} + {{{x^I}} \over r}{\partial _I}f = 0,$$

where *x*^*I*^ denote Cartesian coordinates. Because information only propagates outwards, the spatial derivative *∂*_*I*_*f* is evaluated using a one-sided stencil. This method is straightforwardly generalized to asymptotically expanding cosmological spacetimes of dS type containing BHs; cf. Eq. (9) in Ref. [837]. Even though this approximation appears to work rather (one might be tempted to say surprisingly) well in practice [652], it is important to bear in mind the following caveats. (i) The number of conditions imposed in this way exceeds the number of ingoing characteristics calling into question the well-posedness of the resulting system. (ii) Sommerfeld conditions are not constraint satisfying which leads to systematic errors that do not converge away as resolution is increased. (iii) Some spurious reflections of gravitational waves may occur, especially when applied at too small radii. These potential difficulties of BSSN evolutions have motivated studies of generalizing the BSSN system, in particular the conformal Z4 formulations discussed in Section 6.1.5 which accomodate constraint preserving boundary conditions which facilitate control of the ingoing gravitational radiation [428].

In asymptotically AdS spacetimes, the outer boundary represents a more challenging problem and the difficulties just discussed are likely to impact numerical simulations more severely if not handled appropriately. This is largely a consequence of the singular behaviour of the AdS metric even in the absence of a BH or any matter sources. The AdS metric (see Section 3.3.1) is the maximally symmetric solution to the Einstein equations (39) with *T*_*αβ*_ = 0 and Λ < 0. This solution can be represented by the hyperboloid $X_0^2 + X_D^2 - \sum\nolimits_{i = 1}^{D - 1} {X_i^2}$ embedded in a flat *D* + 1-dimensional spacetime with metric ${\rm{d}}{s^2} = - \;{\rm{d}}X_0^2 - {\rm{d}}X_D^2 + \sum\nolimits_{i = 1}^{D - 1} {{\rm{d}}X_i^2}$. It can be represented in global coordinates, as

130 $${\rm{d}}{s^2} = {{{L^2}} \over {{{\cos}^2}\rho}}(- {\rm{d}}{\tau ^2} + {\rm{d}}{\rho ^2} + {\sin ^2}\rho \,{\rm{d}}\Omega _{D - 2}^2)\,,$$

where 0 ≤ *ρ* < π/2, − π < *τ* ≤ π and Λ = *−*(*D* − 1)(D − 2)/(2*L*^2^) (by unwrapping the cylindrical time direction, the range of the time coordinate is often extended to *τ* ∈ ℝ), or in the Poincarè coordinates:

131 $${\rm{d}}{s^2} = {{{L^2}} \over {{z^2}}}\left[ {- {\rm{d}}{t^2} + {\rm{d}}{z^2} + \sum\limits_{i = 1}^{D - 2} {{{({\rm{d}}{x^i})}^2}}} \right],$$

with *z* > 0, *t*∈ ℝ. It can be shown that Poincaré coordinates only cover half the hyperboloid and that the other half corresponds to *z* < 0 [81].

Clearly, both the global (130) and the Poincaré (131) versions of the AdS metric become singular at their respective outer boundaries *ρ →π*/2 or *z →*0. The induced metric at infinity is therefore only defined up to a conformal rescaling. This remaining freedom manifests itself in the boundary topology of the global and Poincaré metrics which, respecitvely, become in the limit *ρ →π*/2 and *z*→0

132 $${\rm{d}}s_{{\rm{gl}}}^2\sim - {\rm{d}}{\tau ^2} + {\rm{d}}\Omega _{D - 2}^2,\qquad {\rm{d}}s_{\rm{P}}^2\sim - {\rm{d}}{t^2} + \sum\limits_{i = 1}^{D - 2} {{{({\rm{d}}{x^i})}^2}}.$$

In the context of the gauge/gravity duality, gravity in global or Poincaré AdS is related to CFTs on spacetimes of different topology: ℝ × *S*_*D−*2_ in the former and ℝ^*D*−1^ in the latter case.

The boundary treatment inside a numerical modelling of asymptotically AdS spacetimes needs to take care of the singular nature of the metric. In practice, this is achieved through some form of regularization which makes use of the fact that the singular piece of an asymptotically AdS spacetime is known in analytic form, e.g., through Eqs. (130) or (131). In Ref. [70] the spacetime metric is decomposed into an analytically known AdS part plus a deviation which is regular at infinity. In this approach, particular care needs to be taken of the gauge conditions to ensure that the coordinates remain compatible with this decomposition throughout the simulation. An alternative approach consists in factoring out appropriate factors involving the bulk coordinate as for example the term cos *ρ* in the denominator on the right-hand side of Eq. (130). This method is employed in several recent works [207, 415, 108].

We finally note that the boundary plays an active role in AdS spacetimes. The visualization of the AdS spacetime in the form of a Penrose diagram demonstrates that it is not globally hyperbolic, i.e., there exists no Cauchy surface on which initial data can be specified in such a way that the entire future of the spacetime is uniquely determined. This is in marked contrast to the Minkowski spacetime. Put in other words, the outer boundary of asymptotically flat spacetimes is represented in a Penrose diagram by a null surface such that information cannot propagate from infinity into the interior spacetime. In contrast, the outer boundary of asymptotically AdS spacetimes is timelike and, hence, the outer boundary actively influences the evolution of the interior. The specification of boundary conditions in NR applications to the gauge/gravity duality or AdS/CFT correspondence therefore reflects part of the description of the physical system under study; cf. Section 7.8.

### Diagnostics

Once we have numerically generated a spacetime, there still remains the question of how to extract physical information from the large chunk of numbers the computer has written to the hard drive. This analysis of the data faces two main problems in NR applications, (i) the gauge or coordinate dependence of the results and (ii) the fact that many quantities we are familiar with from Newtonian physics are hard or not even possible to define in a rigorous fashion in GR. In spite of these difficulties, a number of valuable diagnostic tools have been developed and the purpose of this section is to review how these are extracted.

The physical information is often most conveniently calculated from the ADM variables and we assume for this discussion that a numerical solution is available in the form of the ADM variables *γ*_*IJ*_, *K*_*IJ*_, *α* and *β*^*I*^. Even if the time evolution has been performed using other variables as for example the BSSN or GHG variables the conversion between these and their ADM counterparts according to Eq. (58) or (43) is straightforward.

One evident diagnostic directly arises from the structure of the Einstein equations where the number of equations exceeds the number of free variables; cf. the discussion following Eq. (55). Most numerical applications employ “free evolutions” where the evolution equations are used for updating the grid variables. The constraints are thus not directly used in the numerical evolution but need to be satisfied by any solution to the Einstein equations. A convergence analysis of the constraints (see for example Figure 3 in Ref. [714]) then provides an important consistency check of the simulations.

Before reviewing the extraction of physical information from a numerical simulation, we note a potential subtlety arising from the convention used for Newton’s constant in higher-dimensional spacetimes. We wrote the Einstein equations in the form (39) and chose units where *G* = 1 and *c* = 1. This implies that the Einstein equations have the form *R*_*αβ*_ − 1/2*Rg*_*αβ*_+Λ*g*_*αβ*_ = 8*πGT*_*αβ*_ for all spacetime dimensionalities (here and in Section 6.7.1 we explicitly keep *G* in the equations). As we shall see below, with this convention the Schwarzschild radius of a static BH in *D* dimensions is given by

133 $$R_{\rm{S}}^{D - 3} = {{16\pi GM} \over {(D - 2){\Omega _{D - 2}}}}\,,\quad {\Omega _{D - 2}} = {{2{\pi ^{(D - 1)/2}}} \over {\Gamma \left({{{D - 1} \over 2}} \right)}},$$

where Ω_*d−*2_ denotes the area of the unit *S*^*D*−2^ sphere.

#### Global quantities and horizons

For spacetimes described by a metric that is asymptotically flat and time independent, the total mass-energy and linear momentum are given by the ADM mass and ADM momentum, respectively. These quantities arise from boundary terms in the Hamiltonian of GR and were derived by Arnowitt, Deser & Misner [47] in their canonical analysis of the theory. They are given in terms of the ADM variables by

134 $${M_{{\rm{ADM}}}} = {1 \over {16\pi G}}\underset {r \rightarrow \infty}{\lim} \oint\nolimits_{{S_r}} {{\delta ^{MN}}({\partial _N}{\gamma _{MK}} - {\partial _K}{\gamma _{MN}}){{\hat r}^K}\;{\rm{d}}S},$$

135 $${P_I} = {1 \over {8\pi G}}\underset {r \rightarrow \infty}{\lim} \oint\nolimits_{{\Omega _r}} {({K_{MI}} - {\delta _{MI}}K){{\hat r}^M}\;{\rm{d}}S}.$$

Here, the spatial tensor components *γ*_*IJ*_ and *K*_*IJ*_ are assumed to be given in Cartesian coordinates, ${\hat r^M} = {x^M}/r$ is the outgoing unit vector normal to the area element *dS* of the *S*^*D−*2^ sphere and *dS* = *r*^*D−*2^ dΩ_*D*−2_. The above integral is defined only for a restricted class of coordinate systems, known as asymptotic Euclidian coordinates for which the metric components are required to be of the form ${g_{\mu \nu}} = {\eta _{\mu \nu}} + \mathcal O(1/r)$. Under a more restrictive set of assumptions about the fall-off behaviour of the metric and extrinsic curvature components (see Sections 7.5.1 and 7.5.2 in [364] and references therein for a detailed discussion), one can also derive an expression for the global angular momentum

136 $${J_I} = {1 \over {8\pi}}\underset {r \rightarrow \infty}{\lim} \oint {({K_{JK}} - K{\gamma _{JK}})} \xi _{(I)}^J{\hat r^K}{\rm{d}}S,$$

where ξ_(*I*)_ are the Killing vector fields associated with the asymptotic rotational symmetry given, in *D* = 4, by ***ξ***_(*x*)_ = *−z****∂***_*y*_ + *y****∂***_*z*_, **ξ**_(*y*)_ = *−x****∂***_*z*_ + *z****∂***_*x*_ and **ξ**(*z*) = −*y***∂**_x_ + *x****∂***_*y*_. For a more-in-depth discussion of the ADM mass and momentum as well as the conditions required for the definition of the angular momentum the reader is referred to Section 7 of [364]. Expressions for *M*_ADM_, *P*_*i*_ and *J*_*I*_ can also be derived in more general (curvilinear) coordinate systems as long as the metric approaches the flat-space form in those curvilinear coordinates at an appropriate rate; see, e.g., Section 7 in [364] for a detailed review.

As an example, we calculate the ADM mass of the *D*-dimensional Schwarzschild BH in Cartesian, isotropic coordinates (*t, x*^*I*^) described by the spatial metric

137 $${\gamma _{IJ}} = {\psi ^{{4 \over {D - 3}}}}{\delta _{IJ}}\,,\quad \psi = 1 + {\mu \over {4{r^{D - 3}}}},$$

and vanishing extrinsic curvature *K*_*IJ*_ = 0. A straightforward calculation shows that

138 $${\partial _K}{\gamma _{IJ}} = - {\psi ^{{4 \over {D - 3}} - 1}}{\mu \over {{r^{D - 1}}}}{x_K}{\delta _{IJ}},$$

so that (since ${x^K} = r{\hat r^K}$)

139 $${\delta ^{MN}}({\partial _N}{\gamma _{MK}} - {\partial _K}{\gamma _{MN}}){{{x^K}} \over r} = (D - 2){\psi ^{{4 \over {D - 3}} - 1}}{{\mu {x_K}} \over {{r^{D - 1}}}}{{{x^K}} \over r} = (D - 2){\mu \over {{r^{D - 2}}}},$$

where we have used the fact that in the limit *r* →*∞* we can raise and lower indices with the Euclidean metric *δ*_*IJ*_ and *ψ* →1. From Eq. (134) we thus obtain

140 $$\begin{array}{*{20}c} {{M_{{\rm{ADM}}}} = {1 \over {16\pi G}}\underset {r \rightarrow \infty}{\lim} \oint\nolimits_{{S_r}} {(D - 2)} {\mu \over {{r^{D - 2}}}}{r^{D - 2}}{\rm{d}}{\Omega _{D - 2}} = {{D - 2} \over {16\pi G}}\mu \;\oint {{\rm{d}}{\Omega _{D - 2}}}}\\ {= {{D - 2} \over {16\pi G}}\mu {\Omega _{D - 2}} = {{D - 2} \over {16\pi G}}\mu {{2{\pi ^{{{D - 1} \over 2}}}} \over {\Gamma \left({{{D - 1} \over 2}} \right)}}.\quad \quad \quad \quad \quad}\\ \end{array}$$

The Schwarzschild radius in areal coordinates is given by $R_{\rm{S}}^{D - 3} = \mu$ and we have recovered Eq. (133).

The event horizon is defined as the boundary between points in the spacetime from which null geodesics can escape to infinity and points from which they cannot. The event horizon is therefore by definition a concept that depends on the entire spacetime. In the context of numerical simulations, this implies that an event horizon can only be computed if information about the entire spacetime is stored which results in large data sets even by contemporary standards. Nevertheless, event horizon finders have been developed in Refs. [278, 223]. For many purposes, however, it is more convenient to determine the existence of a horizon using data from a spatial hypersurface Σ_*t*_ only. Such a tool is available in the form of an AH. AHs are one of the most important diagnostic tools in NR and are reviewed in detail in the *Living Reviews* article [749]. It can be shown under the assumption of cosmic censorship and reasonable energy conditions, that the existence of an AH implies an event horizon whose cross section with Σ_*t*_ either lies outside the AH or coincides with it; see [406, 766] for details and proofs.

The key concept underlying the AH is that of a trapped surface defined as a surface where the expansion Θ = ∇_*μ*_*k*^*μ*^ of a congruence of outgoing null geodesics with tangent vector *k*^*μ*^ satisfies Θ ≤ 0. A marginally trapped surface is defined as a surface where Θ = 0 and an AH is defined as the outermost marginally trapped surface on a spatial hypersurface Σ_*t*_. Translated into the ADM variables, the condition Θ = 0 can be shown to lead to an elliptic equation for the unit normal direction *s*^*I*^ to the *D* − 2-dimensional horizon surface

141 $${q^{MN}}{D_M}{s_N} - K + {K_{MN}}{s^M}{s^N} = 0.$$

Here, *q*_*MN*_ denotes the (*D* − 2)-dimensional metric induced on the horizon surface. Numerical algorithms to solve this equation have been developed by several authors [374, 22, 747, 682, 748].

In the case of a static, spherically symmetric BH, it is possible to use the formula ${A_{{\rm{hor}}}} = {\Omega _{D - 2}}R_{\rm{S}}^{D - 2}$ for the area of a *D* − 2 sphere to eliminate *R*_S_ in Eq. (133). We thus obtain an expression that relates the horizon area to a mass commonly referred to as the *irreducible mass*

142 $${M_{{\rm{irr}}}} = {{(D - 2){\Omega _{D - 2}}} \over {16\pi G}}{\left({{{{A_{{\rm{hor}}}}} \over {{\Omega _{D - 2}}}}} \right)^{{{D - 3} \over {D - 2}}}}.$$

It is possible to derive the same expression in the more general case of a stationary BH, such as the Kerr BH in *D* = 4, or the Myers-Perry BH in *D* > 4.

The irreducible mass, as defined by Eq. 142, is identical to the ADM mass for a static BH. This equation can be used to define the irreducible mass for stationary BHs as well [217]. In *D* = 4 dimensions this becomes $16\pi \;GM_{{\rm{irr}}}^2 = {A_{{\rm{hor}}}}$. Furthermore, a rotating BH in *D* = 4 is described by a single spin parameter *S* and the BH mass consisting of rest mass and rotational energy has been shown by Christodoulou [217] to be given by

143 $${M^2} = M_{{\rm{irr}}}^2 + {{{S^2}} \over {4\,{G^2}M_{{\rm{irr}}}^2}}.$$

By adding the square of the linear momentum *P*^2^ to the right-hand side of this equation we obtain the total energy of a spacetime containing a single BH with spin *S* and linear momentum *P*. In *D* = 4, Christodoulou’s formula (143) can be used to calculate the spin from the equatorial circumference *C*_*e*_ and the horizon area according to [720]

144 $${{2\pi {A_{{\rm{hor}}}}} \over {C_e^2}} = 1 + \sqrt {1 - {j^2}},$$

where *j = S/*(*GM*^2^) is the dimensionless spin parameter of the BH. Even though this relation is strictly valid only for the case of single stationary BHs, it provides a useful approximation in binary spacetimes as long as the BHs are sufficiently far apart.

It is a remarkable feature of BHs that their local properties such as mass and angular momentum can be determined in the way summarized here. In general it is not possible to assign in such a well-defined manner a local energy or momentum content to compact subsets of spacetimes due to the nonlinear nature of GR. For BHs, however, it is possible to derive expressions analogous to the ADM integrals discussed above, but now applied to the apparent horizon. Ultimately, this feature rests on the *dynamic* and *isolated horizon* framework; for more details see [281, 52] and the *Living Reviews* article by Ashtekar & Krishnan [53].

#### Gravitational-wave extraction

Probably the most important physical quantity to be extracted from dynamical BH spacetimes is the gravitational radiation. It is commonly extracted from numerical simulations in the form of either the Newman-Penrose scalar or a master function obtained through BH perturbation theory (see Section 5.2.1). Simulations using a characteristic formulation also facilitate wave extraction in the form of the Bondi mass loss formula. The Landau-Lifshitz pseudo-tensor [500], which has been generalized to *D* > 4 in [820], has been used for gravitational radiation extraction in Ref. [700] for studies of BH stability in higher dimensions; for applications in *D* = 4 see, e.g., [529]. Here, we will focus on the former two methods; wave extraction using the Bondi formalism is discussed in detail in Ref. [788].

**Newman-Penrose scalar**: The formalism to extract GWs in the form of the Newman-Penrose scalar is currently fully understood only in *D* = 4 dimensions. Extension of this method is likely to require an improved understanding of the Goldberg-Sachs theorem in *D* > 4 which is subject to ongoing research [591]. The following discussion is therefore limited to *D* = 4 and we shall further focus on the case of asymptotically flat spacetimes. The Newman-Penrose formalism [575] (see Section 5.2.1) is based on a tetrad of null vectors, two of them real and referred to as ***ℓ***, ***k*** in this work, and two complex conjugate vectors referred to as ***m*** and $\bar m$; cf. Eq. (20) and the surrounding discussion. Under certain conditions the projections of the Weyl tensor onto these tetrad directions may allow for a particularly convenient way to identify the physical properties of the spacetime. More specifically, the 10 independent components of the Weyl tensor are rearranged in the form of 5 complex scalars defined as (see, e.g., [573])

145 $$\begin{array}{*{20}c} {{\Psi _0} = - {C_{\alpha \beta \gamma \delta}}{k^\alpha}{m^\beta}{k^\gamma}{m^\delta},}\\ {{\Psi _1} = - {C_{\alpha \beta \gamma \delta}}{k^\alpha}{\ell ^\beta}{k^\gamma}{m^\delta},}\\ {{\Psi _2} = - {C_{\alpha \beta \gamma \delta}}{k^\alpha}{m^\beta}{{\bar m}^\gamma}{\ell ^\delta},}\\ {{\Psi _3} = - {C_{\alpha \beta \gamma \delta}}{k^\alpha}{\ell ^\beta}{{\bar m}^\gamma}{\ell ^\delta},}\\ {{\Psi _4} = - {C_{\alpha \beta \gamma \delta}}{\ell ^\alpha}{{\bar m}^\beta}{\ell ^\gamma}{{\bar m}^\delta}.}\\ \end{array}$$

The identification of these projections with gravitational radiation is based on the work of Bondi et al. and Sachs [118, 667] and the geometrical construction of Penrose [607] but crucially relies on a correct choice of the null tetrad in Eq. (145) which needs to correspond to a Bondi frame. One example of this type, frequently considered in numerical applications, is the Kinner-sley tetrad [469, 744]. More specifically, one employs a tetrad that converges to the Kinnersley tetrad as the spacetime approaches Petrov type D.^16^ Tetrads with this property are often referred to as *quasi-Kinnersley tetrads* and belong to a class of tetrads which are related to each other by spin/boost transformations; see [82, 572, 832] and references therein. A particularly convenient choice consists in the transverse frame where Ψ_1_ = Ψ_3_ = 0 and the remaining scalars encode the ingoing gravitational radiation (Ψ_0_), the outgoing radiation (Ψ_4_) and the static or *Coulomb* part of the gravitational field (Ψ_2_). The construction of suitable tetrads in dynamic, numerically generated spacetimes represents a non-trivial task and is the subject of ongoing research (see, for example, [158, 510, 571, 832]).

For reasons already discussed in Section 6.6, extraction of gravitational waves is often performed at finite distance from the sources; but see Refs. [642, 60] for Cauchy-characteristic extraction that facilitates GW calculation at future null infinity. GW extraction at finite distances requires further ingredients which are discussed in more detail in [510]. These include a specific asymptotic behaviour of the Riemann tensor, the so-called *peeling property* [666, 667, 575], that outgoing null hypersurfaces define sequences of *S*^2^ spheres which are conformal to unit spheres and a choice of coordinates that ensures appropriate fall-off of the metric components in the extraction frame.

Extraction of GWs at finite extraction radii *r*_ex_ is therefore affected by various potential errors. An attempt to estimate the uncertainty arising from the use of finite *r*_ex_ consists in measuring the GW signal at different values of the radius and analyzing its behaviour as the distance is increased. Convergence of the signal as 1/*r*_ex_ →0 may then provide some estimate for the error incurred and improved results may be obtained through extrapolation to infinite *r*_ex_; see, e.g., [124, 429]. While such methods appear to work relatively well in practice (applying balance arguments together with measurements of BH horizon masses and the ADM mass or comparison with alternative extraction methods provide useful checks), it is important to bear in mind the possibility of systematic errors arising in the extraction of GWs using this method.

In the following discussion we will assume that the above requirements are met and describe a frequently used recipe that leads from the metric components of a numerical simulation to estimates of the energy and momenta contained in the gravitational radiation. The first step in the calculation of Ψ_4_ from the ADM metric is to construct the null tetrad. An approximation to a quasi-Kinnersley tetrad is given in terms of the unit timelike normal vector *n*^*α*^ introduced in Section 6.1, and a triad *u*^*i*^, *υ*^*i*^, *w*^*i*^ of spatial vectors on each surface Σ_*t*_ constructed through Gram-Schmidt orthonormalization starting with

146 $${u^i} = [x,\,y,\,z]\,,\qquad {v^i} = [xz,\,yz,\, - {x^2} - {y^2}],\qquad {w^i} = {\epsilon^i}_{mn}{u^m}{v^n}.$$

Here, *ϵ*^*imm*^ represents the three-dimensional Levi-Civita tensor on Σ_*t*_ and *x, y, z* are standard Cartesian coordinates. An orthonormal tetrad is then obtained from

147 $${k^\alpha} = {1 \over {\sqrt 2}}({n^\alpha} + {u^\alpha}),\qquad {\ell ^\alpha} = {1 \over {\sqrt 2}}({n^\alpha} - {u^\alpha}),\qquad {m^\alpha} = {1 \over {\sqrt 2}}({v^\alpha} + i{w^\alpha}),$$

where time components of the spatial triad vectors vanish by construction.

Then, the calculation of Ψ_4_ from the ADM variables can be achieved either by constructing the spacetime metric from the spatial metric, lapse and shift vector and computing the spacetime Riemann or Weyl tensor through their definitions (see the preamble on “notation and conventions”). Alternatively, we can use the electric and magnetic parts of the Weyl tensor given by [334]

148 $${E_{\alpha \beta}} = {\bot ^\mu}_\alpha {\bot ^\nu}_\beta {C_{\mu \rho \nu \sigma}}{n^\rho}{n^\sigma}\,,\qquad {B_{\alpha \beta}} = {\bot ^\mu}_\alpha {\bot ^\nu}_\beta {\,^{\ast}}{C_{\mu \rho \nu \sigma}}{n^\rho}{n^\sigma},$$

where the * denotes the Hodge dual. By using the Gauss-Codazzi equations (47), one can express the electric and magnetic parts in vacuum in terms of the ADM variables according to^17^

149 $${E_{ij}} = {{\mathcal R}_{ij}} - {\gamma ^{mn}}({K_{ij}}{K_{mn}} - {K_{im}}{K_{jn}})\,,\quad {B_{ij}} = {\gamma _{ik}}{\epsilon^{kmn}}{D_m}{K_{nj}}.$$

In vacuum, the Weyl tensor is then given in terms of electric and magnetic parts by Eq. (3.10) in Ref. [334]. Inserting this relation together with (147) and (149) into the definition (145) gives us the final expression for Ψ_4_ in terms of spatial variables

150 $$\begin{array}{*{20}c} {{\Psi _4} = - {1 \over 2}[{E_{mn}}({v^m}{v^n} - {w^m}{w^n}) - {B_{mn}}({v^m}{v^n} + {w^m}{w^n})]\quad \quad \;}\\ {+ {i \over 2}[{E_{mn}}({v^m}{w^n} - {w^m}{v^n}) + {B_{mn}}({w^m}{w^n} + {v^m}{v^n})].}\\ \end{array}$$

The GW signal is often presented in the form of multipolar components *ψ*_*ℓm*_ defined by projection of Ψ_4_ onto spherical harmonics of spin weight −2 [356]

151 $${\Psi _4}(t,\theta, \phi) = \sum\limits_{lm} {{\psi _{lm}}} (t)Y_{lm}^{(- 2)}(\theta, \phi) \Leftrightarrow {\psi _{lm}}(t) = \int {{\Psi _4}} (t, \theta, \phi)\overline {Y_{lm}^{(- 2)}} (\theta, \phi){\rm{d}}{\Omega _2},$$

where the bar denotes the complex conjugate. The *ψ*_*lm*_ are often written in terms of amplitude and phase

152 $${\psi _{lm}} = {A_{lm}}{e^{i{\phi _{lm}}}}.$$

The amount of energy, linear and angular momentum carried by the GWs can be calculated from Ψ_4_ according to [663]

153 $${{{\rm{d}}E} \over {{\rm{d}}t}} = \underset{r \rightarrow \infty}{\lim} \left[ {{{{r^2}} \over {16\pi}}\int\nolimits_{{\Omega _2}} {{{\left\vert {\int\nolimits_{- \infty}^t {{\Psi _4}\;{\rm{d}}\tilde t}} \right\vert}^2}\;{\rm{d}}\Omega}} \right],$$

154 $${{{\rm{d}}{P_i}} \over {{\rm{d}}t}} = - \underset{r \rightarrow \infty}{\lim} \left[ {{{{r^2}} \over {16\pi}}\int\nolimits_{{\Omega _2}} {{\ell _i}{{\left\vert {\int\nolimits_{- \infty}^t {{\Psi _4}\;{\rm{d}}\tilde t}} \right\vert}^2}\;{\rm{d}}\Omega}} \right],$$

155 $${{{\rm{d}}{J_i}} \over {{\rm{d}}t}} = - \underset{r \rightarrow \infty}{\lim} \left\{{{{{r^2}} \over {16\pi}}{{\rm Re}} \left[ {\int\nolimits_{{\Omega _2}} {\left({{{\hat J}_i}\int\nolimits_{- \infty}^t {{\Psi _4}\;{\rm{d}}\tilde t}} \right)\left({\int\nolimits_{- \infty}^t {\int\nolimits_{- \infty}^{\hat t} {{{\bar \Psi}_4}\;{\rm{d}}\tilde t\;{\rm{d}}\hat t}}} \right)\;{\rm{d}}\Omega}} \right]} \right\},$$

where

156 $${\ell _i} = [ - \sin \theta \cos \phi, \; - \sin \theta \sin \phi, \; - \cos \theta ],$$

157 $${\hat J_x} = - \sin \phi \;{\partial _\theta} - \cos \phi \left({\cot \theta \;{\partial _\phi} - {{2i} \over {\sin \theta}}} \right),$$

158 $${\hat J_y} = \cos \phi \;{\partial _\theta} - \sin \phi \left({\cot \theta \;{\partial _\phi} - {{2i} \over {\sin \theta}}} \right),$$

159 $${\hat J_z} = {\partial _\phi}.$$

In practice, one often starts the integration at the start of the numerical simulation (or shortly thereafter to avoid contamination from spurious GWs contained in the initial data) rather than at −∞.

We finally note that the GW strain commonly used in GW data analysis is obtained from Ψ_4_ by integrating twice in time

160 $$h \equiv {h_ +} - i{h_ \times} = \int\nolimits_{- \infty}^t {\left({\int\nolimits_{- \infty}^{\tilde t} {{\Psi _4}} {\rm{d}}\hat t} \right)} {\rm{d}}\tilde t.$$

*h* is often decomposed into multipoles in analogy to Eq. (151). As before, the practical integration is often started at finite value rather than at −∞. It has been noted that this process of integrating Ψ_4_ twice in time is susceptible to large nonlinear drifts. These are due to fundamental difficulties that arise in the integration of finite-length, discretly sampled, noisy data streams which can be cured or at least mitigated by performing the integration in the Fourier instead of the time domain [644, 429].

**Perturbative wave extraction**: The basis of this approach to extract GWs from numerical simulations in *D* = 4 is the Regge-Wheeler-Zerilli-Moncrief formalism developed for the study of perturbations of spherically symmetric BHs. The assumption for applying this formalism to numerically generated spacetimes is that at sufficiently large distances from the GW sources, the spacetime is well approximated by a spherically symmetric background (typically Schwarzschild or Minkowski spacetime) plus non-spherical perturbations. These perturbations naturally divide into odd and even multipoles which obey the Regge-Wheeler [641] (odd) and the Zerilli [830] (even) equations respectively (see Section 5.2.1). Moncrief [555] developed a gauge-invariant formulation for these perturbations in terms of a master function which obeys a wave-type equation with a background dependent scattering potential; for a review and applications of this formalism see for example [566, 721, 643].

An extension of this formalism to higher-dimensional spacetimes has been developed by Kodama & Ishibashi [479], and is discussed in Section 5.2.3. This approach has been used to develop wave extraction from NR simulations in *D* > 4 with *SO*(*D* − 2) symmetry [797]. In particular, it has been applied to the extraction of GWs from head-on collisions of BHs. As in our discussion of formulations of the Einstein equations in higher dimensions in Section 6.2, it turns out useful to introduce coordinates that are adapted to the rotational symmetry on a *S*^*D−*2^ sphere. Here, we choose spherical coordinates for this purpose which we denote by (*t*, *r*, *ϑ*, *θ*, *ϕ*^a^) where *a* = 4, …, *D* − 1; we use the same convention for indices as in Section 6.2.

We then assume that in the far-field region, the spacetime is perturbatively close to a spherically symmetric BH background given in *D* dimensions by the Tangherlini [740] metric

161 $${\rm{d}}s_{(0)}^2 = - A{(r)^2}{\rm{d}}{t^2} + A{(r)^{- 1}}{\rm{d}}{r^2} + {r^2}\left[ {{\rm{d}}{\vartheta ^2} + {{\sin}^2}\vartheta \,({\rm{d}}{\theta ^2} + {{\sin}^2}\theta \,{\rm{d}}{\Omega _{D - 4}})} \right],$$

where

162 $$A(r) = 1 - {{R_{\rm{S}}^{D - 3}} \over {{r^{D - 3}}}},$$

and the Schwarzschild radius RS is related to the BH mass through Eq. (133). For a spacetime with *SO*(*D* − 3) isometry the perturbations away from the background (161) are given by

163 $${\rm{d}}s_{(1)}^2 = {h_{AB}}\;{\rm{d}}{x^A}\;{\rm{d}}{x^B} + {h_{A\vartheta}}\;{\rm{d}}{x^A}\;{\rm{d}}\vartheta + {h_{\vartheta \vartheta}}\;{\rm{d}}{\vartheta ^2} + {h_{\theta \theta}}\;{\rm{d}}{\Omega _{D - 3}},$$

where we introduce early upper case Latin indices *A*, *B*, … = 0, 1 and *x*^*A*^ = (*t*, *r*). The class of axisymmetric spacetimes considered in [797] obeys *SO*(*D* − 2) isometry which can be shown to imply that *h*_*Aθ*_ = *h*_*ϑθ*_ = 0 and that the remaining components of *h* in Eq. (163) only depend on the coordinates (*t*, *r*, *ϑ*). As a consequence, only the perturbations which we have called in Section 5.2.3 “scalar” are non-vanishing, and are expanded in tensor spherical harmonics; cf. Section II C in Ref. [797].

As discussed in Section 5.2.3, the metric perturbations, decomposed in tensor harmonics, can be combined in a gauge-invariant master function Φ_*ℓm*_. From the master function, we can calculate the GW energy flux and the total radiated energy as discussed in Section 5.2.3.

#### Diagnostics in asymptotically AdS spacetimes

The gauge/gravity duality, or AdS/CFT correspondence (see Section 3.3.1), relates gravity in asymptotically AdS spacetimes to conformal field theories on the boundary of this spacetime. A key ingredient of the correspondence is the relation between fields interacting gravitationally in the bulk spacetime and expectation values of the field theory on the boundary. Here we restrict our attention to the extraction of the expectation values of the energy-momentum tensor 〈*T*_*IJ*_〉 of the field theory from the fall-off behaviour of the AdS metric.

Through the AdS/CFT correspondence, the expectation values 〈*T*_*IJ*_〉 of the field theory are given by the quasi-local Brown-York [139] stress-energy tensor and thus are directly related to the bulk metric. Following [253], it is convenient to consider the (asymptotically AdS) bulk metric in Fefferman-Graham [314] coordinates

164 $${\rm{d}}{s^2} = {g_{\mu \nu}}{\rm{d}}{x^\mu}{\rm{d}}{x^\nu} = {{{L^2}} \over {{r^2}}}\left[ {{\rm{d}}{r^2} + {\gamma _{IJ}}{\rm{d}}{x^I}{\rm{d}}{x^J}} \right],$$

where

165 $${\gamma _{IJ}} = {\gamma _{IJ}}(r,{x^I}) = {\gamma _{(0)IJ}} + {r^2}{\gamma _{(2)IJ}} + \ldots + {r^d}{\gamma _{(d)IJ}} + {h_{(d)IJ}}{r^d}\log {r^2} + {\mathcal O}({r^{d + 1}}).$$

Here *d* ≡ *D* − 1, the *γ*_(*a*)*IJ*_ and *h*_(*d*)*IJ*_ are functions of the boundary coordinates *x*^*i*^, the logarithmic term only appears for even *d* and powers of r are exclusively even up to order *d − 1*. As shown in Ref. [253], the vacuum expectation value of the CFT momentum tensor for *d* = 4 is then obtained from

166 $$\begin{array}{*{20}c} {\langle {T_{I\,J}}\rangle = {{4{L^3}} \over {16\pi}}\left\{{{\gamma _{(4)I\,J}} - {1 \over 8}{\gamma _{(0)\,I\,J}}\;\left[ {\gamma _{(2)}^2 - \gamma _{(0)}^{K\,M}\gamma _{(0)}^{L\,N}{\gamma _{(2)K\,L}}{\gamma _{(2)M\,N}}} \right]} \right.} \\ {\left. {- {1 \over 2}{\gamma _{(2)I}}^M{\gamma _{(2)J\,M}} + {1 \over 4}{\gamma _{(2)I\,J}}{\gamma _{(2)}}} \right\}\;\,,\quad \quad \quad \quad} \\ \end{array}$$

and γ_(2)*IJ*_ is determined in terms of γ_(0)*IJ*_. The dynamical freedom of the CFT is thus encapsulated in the fourth-order term γ_(4)*IJ*_. If *γ*_(0)*IJ*_ = *η*_*IJ*_ for *r* →0 the metric (164) asymptotes to the AdS metric in Poincaré coordinates (131).

The Brown-York stress tensor is also the starting point for an alternative method to extract the 〈*T*_*IJ*_〉 that does not rely on Fefferman-Graham coordinates. It is given by

167 $${T^{\mu \nu}} = {2 \over {\sqrt {- \gamma}}}{{\delta {S_{{\rm{grav}}}}} \over {\delta {\gamma _{\mu \nu}}}},$$

where we have foliated the *D*-dimensional spacetime into *timelike* hypersurfaces Σ_*r*_ in analogy to the foliation in terms of *spacelike* hypersurfaces Σ_*t*_ in Section 6.1.1. The spacetime metric is given by

168 $${\rm{d}}{s^2} = {\alpha ^2}{\rm{d}}{r^2} + {\gamma _{IJ}}({\rm{d}}{x^I} + {\beta ^I}{\rm{d}}r)({\rm{d}}{x^J} + {\beta ^J}{\rm{d}}r){.}$$

In analogy to the second fundamental form *K*_*αβ*_ in Section 6.1.1, we define the extrinsic curvature on Σ_*r*_ by

169 $${\Theta ^{\mu \nu}} \equiv - {1 \over 2}({\nabla ^\mu}{n^\nu} + {\nabla ^\nu}{n^\mu}),$$

where *n*^*μ*^ denotes the outward pointing normal vector to Σ_*r*_. Reference [67] provides a method to cure divergencies that appear in the Brown-York tensor when the boundary is pushed to infinity by adding counterterms to the action *S*_grav_. This work discusses asymptotically AdS spacetimes of different dimensions. For AdS_5_, the procedure results in

170 $${T^{\mu \nu}} = {1 \over {8\pi}}\left[ {{\Theta ^{\mu \nu}} - \Theta {\gamma ^{\mu \nu}} - {3 \over L}{\gamma ^{\mu \nu}} - {L \over 2}{{\mathcal G}^{\mu \nu}}} \right],$$

where ${\mathcal G_{\mu \nu}} = {\mathcal R_{\mu \nu}} - \mathcal R{\gamma _{\mu \nu}}/2$ is the Einstein tensor associated with the induced metric γ_*μν*_. Applied to the AdS_5_ metric in global coordinates, this expression gives a non-zero energy-momentum tensor *T*^*μν*^ which, translated into the expectation values 〈*T*^*μν*^〉, can be interpreted as the Casimir energy of a quantum field theory on the spacetime with topology ℝ×*S*^3^ [67]. This Casimir energy is non-dynamical and in numerical applications to the AdS/CFT correspondence may simply be subtracted from *T*^*μν*^; see, e.g., [70].

The role of additional (e.g., scalar) fields in the AdS/CFT dictionary is discussed, for example, in Refs. [253, 705].

## Applications of Numerical Relativity

Numerical relativity was born out of efforts to solve the two-body problem in GR, and aimed mainly at understanding stellar collapse and GW emission from BH and NS binaries. There is therefore a vast amount of important results and literature on NR in astrophysical contexts. Because these results fall outside the scope of this review, we refer the interested reader to Refs. [631, 592, 191, 715, 18, 617, 429] and to the relevant sections of *Living Reviews*^18^ for (much) more on this subject. Instead, we now focus on applications of NR outside its traditional realm, most of which are relatively recent new directions in the field.

### Critical collapse

The nonlinear stability of Minkowski spacetime was established by Christodoulou and Klainerman, who showed that arbitrarily “small” initial fluctations eventually disperse to infinity [219]. On the other hand, large enough concentrations of matter are expected to collapse to BHs, therefore raising the question of how the threshold for BH formation is approached.

Choptuik performed a thorough investigation of this issue, by evolving initial data for a minimally coupled massless scalar field [212]. Let the initial data be described by a parameter *p* which characterizes the initial scalar field wavepacket. For example, in Choptuik’s analysis, the following family of initial data for the scalar field Φ was considered,

171 $$\phi = {\phi _0}{r^3}\exp \left({- {{[(r - {r_0})/\delta ]}^q}} \right),$$

where Φ = *ϕ*′; therefore any of the quantities *ϕ*_0_, *r0, δ, q* is a suitable parameter *p*.

**Figure 6:**
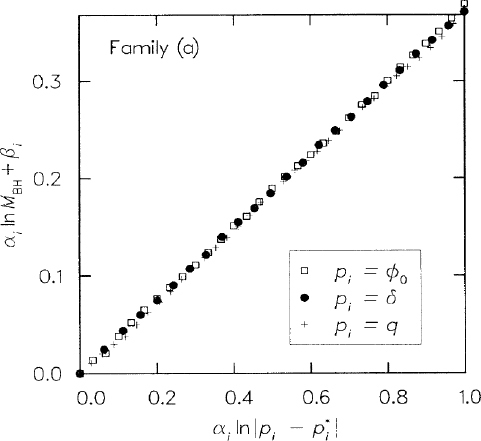
Illustration of the conjectured mass-scaling relation (172). The data refer to three separate one-parameter variations of the pulse shape (171). The constants *α*_*i*_ and *β*_*i*_ are chosen to normalize the ranges of the abscissa and place the data point corresponding to the smallest BH in each family at the origin. Image reproduced with permission from [212], copyright by APS.

The evolution of such initial data close to the threshold of BH formation is summarized in Figure 6. Fix all but one parameter, say the scalar field amplitude *p = ϕ*_0_. For large amplitudes *ϕ*_0_, a large BH is formed. As the amplitude of the initial data decreases, the mass of the formed BH decreases, until a critical threshold amplitude *ϕ*_0_* is reached below which no BH forms and the initial data disperses away (consistently with the nonlinear stability of Minkowski). Near the threshold, BHs with arbitrarily small masses can be created, and the BH mass scales as

172 $$M \propto {(p - {p_\ast})^\gamma},$$

It was found that γ ≈ 0.37 is a universal (critical) exponent which does not depend on the initial data, or in other words it does not depend on which of the parameters *ϕ*_0_, *r*_0_, *δ,q* is varied (but it may depend on the type of collapsing material).

The BH threshold in the space of initial data for GR shows both surprising structure and surprising simplicity. In particular, critical behavior was found at the threshold of BH formation associated with universality, power-law scaling of the BH mass, and discrete self-similarity, which bear resemblance to more familiar statistical physics systems. Critical phenomena also provide a route to develop arbitrarily large curvatures visible from infinity (starting from smooth initial data) and are therefore likely to be relevant for cosmic censorship (see Section 7.2), quantum gravity, astrophysics, and our general understanding of the dynamics of GR.

Choptuik’s original result was extended in many different directions, to encompass massive scalar fields [125, 586], collapse in higher dimensions [344] or different gravitational theories [265]. Given the difficulty of the problem, most of these studies have focused on 1 + 1 simulations; the first non spherically (but axially) symmetric simulations were performed in Ref. [9], whereas recently the first 3 + 1 simulations of the collapse of minimally coupled scalar fields were reported [411]. The attempt to extend these results to asymptotically AdS spacetimes would uncover a new surprising result, which we discuss below in Section 7.4. A full account of critical collapse along with the relevant references can be found in a *Living Reviews* article on the subject [381].

### Cosmic censorship

As discussed in Section 3.2.1, an idea behind cosmic censorship is that classical GR is self-consistent for physical processes. That is, despite the fact that GR predicts the formation of singularities, at which geodesic incompleteness occurs and therefore failure of predictablity, such singularities should be — for physical processes^19^ — causally disconnected from distant observers by virtue of horizon cloaking. In a nutshell: a GR evolution does not lead, generically, to a system GR cannot tackle. To test this idea, one must analyze strong gravity dynamics, which has been done both using numerical evolutions and analytical arguments. Here we shall focus on recent results based on NR methods. The interested reader is referred to some historically relevant numerical [692, 361] and analytical [218, 653] results, as well as to reviews on the subject [768, 88, 649, 461] for further information.

The simplest (and most physically viable) way to violate cosmic censorship would be through the gravitational collapse of very rapidly rotating matter, possibly leading to a Kerr naked singularity with *a > M*. However, NR simulations of the collapse of a rotating NS to a BH [568, 63, 348] have shown that when the angular momentum of the collapsing matter is too large, part of the matter bounces back, forming an unstable disk that dissipates the excess angular momentum, and eventually collapses to a Kerr BH. Simulations of the coalescence of rapidly rotating BHs [769, 412] and NSs [465] have shown that the *a* > *M* bound is preserved by these processes as well. These simulations provide strong evidence supporting the cosmic censorship conjecture. Let us remark that analytical computations and NR simulations show that naked singularities can arise in the collapse of ideal fluids [461] but these processes seem to require fine-tuned initial conditions, such as in spherically symmetric collapse or in the critical collapse [375] discussed in Section 7.1.

**Figure 7:**
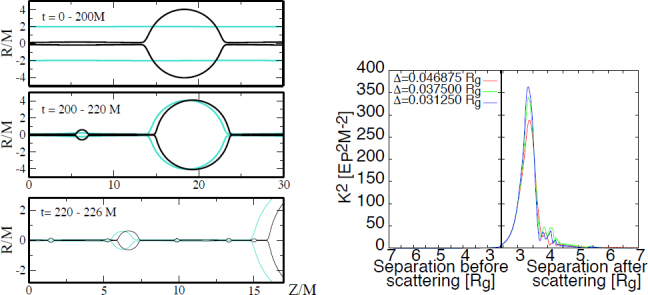
*Left panel*: Embedding diagram of the AH of the perturbed black string at different stages of the evolution. The light (dark) lines denote the first (last) time from the evolution segment shown in the corresponding panel. *Right panel*: Dimensionless Kretschmann scalar ${\mathcal K^2}$ at the centre of mass of a binary BH system as a function of the (areal) coordinate separation between the two BHs in a *D* = 5 scattering, in units of *R*_*g*_ = *R*_*s*_. Images reproduced with permission from (left) [511] and from (right) [587], copyright by APS.

A claim of cosmic censorship violation in *D* > 4 spacetime dimensions was made in the context of the nonlinear evolution of the Gregory-Laflamme (see Section 3.2.4) instability for black strings. In Ref. [511] long-term numerical simulations were reported showing that the development of the instability leads to a cascade of ever smaller spherical BHs connected by ever thinner black string segments — see Figure 7, left (top, middle and bottom) panel for a visualization of the (first, second and third) generations of spherical BHs and string segments. Observe, from the time scales presented in Figure 7, that as viewed by an asymptotic observer, each new generation develops more rapidly than the previous one. The simulations therefore suggest that arbitrarily thin strings, and thus arbitrarily large curvature at the horizon, will be reached in *finite* asymptotic time. If true, this system is an example where a classical GR evolution is driving the system to a configuration that GR cannot describe, a state of affairs that will presumably occur when Planck scale curvatures are attained at the horizon. The relevance of this example for cosmic censorship, may, however, be questioned, based on its higher dimensionality and the lack of asymptotic flatness: cosmic strings with horizons require the spacetime dimension to be greater or equal to five and the string is infinitely extended in one dimension. In addition, the simulations of [511] assume cylindrical symmetry, and cylindrically symmetric matter configurations are unstable [174]; therefore, fine-tuning of initial conditions may be required for the formation of a naked singularity.

Another suggestion that Planckian scale curvature becomes visible in a classical evolution in *D* = 5 GR arises in the high-energy scattering of BHs. In Ref. [587], NR simulations of the scattering of two non-spinning boosted BHs with an impact parameter *b* were reported. For sufficiently small initial velocities (*υ ≲* 0.6*c*) it is possible to find the threshold impact parameter *b*_scat_ = *b*_scat_(*υ*) such that the BHs merge into a (spinning) BH for *b* < *b*_scat_ or scatter off to infinity for *b* > *b*_scat_. For high velocities, however, only a lower bound on the impact parameter for scattering *b*_*C*_ = *b*_*C*_(*υ*) and an upper bound on the impact parameter for merger *b*_*B*_ = *b*_*B*_(*υ*) could be found, since simulations with *b*_*B*_ < *b* < *b*_*C*_ crashed before the final outcome could be determined (cf. Figure 16 below). Moreover, an analysis of a scattering configuration with *υ* = 0.7 and *b* = *b*_*C*_, shows that very high curvature develops outside the individual BHs’ AHs, shortly after they have reached their minimum separation — see Figure 7 (right panel). The timing for the creation of the high curvature region, i.e., that it occurs *after* the scattering, is in agreement with other simulations of high energy collisions. For instance, in Refs. [216, 288] BH formation is seen to occur in the wake of the collision of non-BH objects, which was interpreted as due to focusing effects [288]. In the case of Ref. [587], however, there seems to be no (additional) BH formation. Both the existence and significance of such a high curvature region, seemingly uncovered by any horizon, remains mysterious and deserves further investigation.

In contrast with the two higher-dimensional examples above, NR simulations that have tested the cosmic censorship conjecture in different *D* = 4 setups, found support for the conjecture. We have already mentioned simulations of the gravitational collapse of rotating matter, and of the coalescence of rotating BH and NS binaries. As we discuss below in Section 7.6, the high-energy head-on collisions of BHs [719], boson stars [216] or fluid particles [288, 647] in *D* = 4 result in BH formation but no naked singularities. A different check of the conjecture involves asymptotically dS spacetimes [837]. Here, the cosmological horizon imposes an upper limit on the size of BHs. Thus one may ask what is the outcome of the collision of two BHs with almost the maximum allowed size. In Ref. [837] the authors were able to perform the evolution of two BHs, initially at rest with the cosmological expansion. They observe that for all the (small) initial separations attempted, a cosmological AH, as viewed by an observer at the center of mass of the binary BH system, eventually forms in the evolution, and both BH AHs are outside the cosmological one. In other words, the observer in the center of mass loses causal contact with the two BHs which fly apart rather than merge. This suggests that the background cosmological acceleration dominates over the gravitational attraction between’ large’ BHs. It would be interesting to check if a violation of the conjecture can be produced by introducing opposite charges to the BHs (to increase their mutual attraction) or give them mutually directed initial boosts.

### Hoop conjecture

The hoop conjecture, first proposed by K. Thorne in 1972 [750], states that when the mass *M* of a system (in *D* = 4 dimensions) gets compacted into a region whose circumference in every direction has radius *R* ≲ *R*_*s*_ = 2*M*, a horizon — and thus a BH — forms (for a generalization in *D* > 4, see [449]). This conjecture is important in many contexts. In high-energy particle collisions, it implies that a classical BH forms if the center-of-mass energy significantly exceeds the Planck energy. This is the key assumption behind the hypothesis — in the TeV gravity scenario — of BH production in particle accelerators (see Section 3.3.2). In the trans-Planckian regime, the particles can be treated as classical objects. If two such “classical” particles with equal rest mass *m*_0_ and radius (corresponding to the de Broglie wavelength of the process) *R* collide with a boost parameter γ, the mass-energy in the centre-of-mass frame is *M* = 2*γm*_0_. The threshold radius of Thorne’s hoop is then *R*_s_ = 4*γm*_0_ and the condition *R* ≲ *R*_*s*_ = 2*M* = 4*γm*_0_ translates into a bound on the boost factor, γ ≳ γ_h_ ≡ *R*/(4*m*_0_).

Even though the hoop conjecture seems plausible, finding a rigorous proof is not an easy task. In the last decades, the conjecture has mainly been supported by studies of the collision of two infinitely boosted point particles [256, 286, 817, 818], but it is questionable that they give an accurate description of an actual particle collision (see, e.g., the discussion in Ref. [216]). In recent years, however, advances in NR have made it possible to model trans-Planckian collisions of massive bodies and provided more solid evidence in favor of the validity of the hoop conjecture.

The hoop conjecture has been first addressed in NR by Choptuik & Pretorius [216], who studied head-on collisions of boson stars in four dimensions (see Section 4.2). The simulations show that the threshold boost factor for BH formation is ∼ 1/3*γ*_h_ (where the “hoop” critical boost factor *γ*_h_ is defined above), well in agreement with the hoop conjecture. These results have been confirmed by NR simulations of fluid star collisions [288], showing that a BH forms when the boost factor is larger than ∼ 0.42ϒ_h_. Here, the fluid balls are modeled as two superposed Tolman-Oppenheimer-Volkoff “stars” with a Γ = 2 polytropic equation of state.

**Figure 8:**
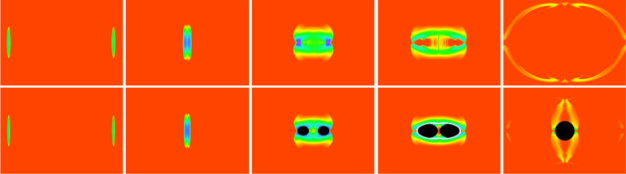
Snapshots of the rest-mass density in the collision of fluid balls with boost factor ϒ = 8 (upper panels) and ϒ = 10 (lower panels) at the initial time, shortly after collision, at the time corresponding to the formation of separate horizons in the ϒ = 10 case, and formation of a common horizon (for ϒ = 10) and at late time in the dispersion (ϒ = 8) or ringdown **(**ϒ = 10) phase. Image reproduced with permission from [288], copyright by APS.

The simulations furthermore show that for boosts slightly above the threshold of BH formation, there exists a brief period where two individual AHs are present, possibly due to a strong focusing of the fluid elements of each individual star caused by the other’s gravitational field. These results are illustrated in Figure 8 which displays snapshots of a collision for ϒ = 8 that does not result in horizon formation (upper panels) and one at ϒ = 10 that results in a BH (lower panels). We remark that the similarity of the behaviour of boson stars and fluid stars provides evidence supporting the “matter does not matter” hypothesis discussed below in Section 7.6. A similar study of colliding fluid balls [647] has shown similar results. Therein it has been found that a BH forms when the compactness of the star is ${m_0}/R\underset{\sim}{>}\;0.08{\gamma ^{- 1.13}}$, i.e., for ϒ^1.13^ ≳ *R*/(12*m*_0_) = 1/3_ϒ*h*_. Type-I critical behaviour has also been identified, with BH formation for initial masses *m*_0_ above a critical value scaling as ∼ ϒ^−1.0^.

### Spacetime stability

Understanding the stability of stationary solutions to the Einstein field equations, or generalisations thereof, is central to gauge their physical relevance. If the corresponding spacetime configuration is to play a role in a given dynamical process, it should be stable or, at the very least, its instabilities should have longer time scales than those of that dynamical process. Following the evolution of unstable solutions, on the other hand, may unveil smoking guns for establishing their transient existence. NR provides a unique tool both for testing nonlinear stability and for following the nonlinear development of unstable solutions. We shall now review the latest developments in both these directions, but before doing so let us make a remark. At the linear level, typical studies of space-time stability are in fact studies of *mode stability*. A standard example is Whiting’s study of the mode stability for Kerr BHs [780]. For BH spacetimes, however, mode stability does not guarantee *linear stability*, cf. the discussion in [236]. We refer the reader to this reference for further information on methods to analyse linear stability.

Even if a spacetime does not exhibit unstable modes in a linear analysis it may be unstable when fully nonlinear dynamics are taken into account. A remarkable illustration of this possibility is the turbulent instability of the AdS spacetime reported in Ref. [108]. These authors consider Einstein gravity with a negative cosmological constant Λ and minimally coupled to a massless real scalar field ϕ in *D* = 4 spacetime dimensions. The AdS metric is obviously a solution of the system together with a constant scalar field. Linear scalar-field perturbations around this solution generate a spectrum of normal modes with real frequencies [150]: *ω*_*N*_*L* = 2*N* + 3+*ℓ*, where *N ∈* ℕ_0_ and *L*, *ℓ* are the AdS length scale and total angular momentum harmonic index, respectively. The existence of this discrete spectrum is quite intuitive from the global structure of AdS: a time-like conformal boundary implies that AdS behaves like a cavity. Moreover, the fact that the frequencies are real shows that the system is stable against scalar-field perturbations at linear level.

**Figure 9:**
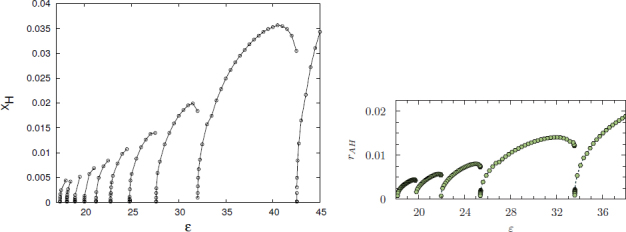
Instability against BH formation in AdS (left panel) and Minkowski enclosed in a cavity (right panel). In both panels, the horizontal axis represents the amplitude of the initial (spherically symmetric) scalar field perturbation. The vertical axis represents the size of the BH formed. Perturbations with the largest plotted amplitude collapse to form a BH. As the amplitude of the perturbation is decreased so does the size of the BH, which tends to zero at a first threshold amplitude. Below this energy, no BH is formed in the first generation collapse and the scalar perturbation scatters towards the boundary. But since the spacetime behaves like a cavity, the scalar perturbation is reflected off the boundary and re-collapses, forming now a BH during the second generation collapse. At smaller amplitudes a second, third, etc, threshold amplitudes are found. The left (right) panel shows ten (five) generations of collapse. Near the threshold amplitudes, critical behavior is observed. Images reproduced with permission from (left) [108] and from (right) [537], copyright by APS.

The latter conclusion dramatically changes when going beyond linear analysis. Setting up spherically symmetric Gaussian-type initial data of amplitude *ϵ*, Bizoń and Rostworowski [108] made the following observations. For large *ϵ* the wave packet collapses to form a BH, signalled by an AH at some radial coordinate. As *ϵ* is made smaller, the AH radius also decreases, reaching zero size at some (first) threshold amplitude. This behaviour is completely analogous to that observed in asymptotically flat spacetime by Choptuik [212] and discussed in Section 7.1; in fact, the solutions obtained with this threshold amplitude asymptote — far from the AdS boundary — to the self-similar solution obtained in the Λ = 0 case. For amplitudes slightly below the first threshold value, the wave packet travels to the AdS boundary, where it is reflected, and collapses to form a BH upon the second approach to the centre. By further decreasing the amplitude, one finds a second threshold amplitude at which the size of the AH formed in this second generation interaction decreases to zero. This pattern seems to repeat itself indefinitely. In [108] ten generations of collapse were reported, as shown in Figure 9 (left panel). These results were confirmed and extended in subsequent work [142]. If indeed the pattern described in the previous paragraph repeats itself indefinitely, a remarkable conclusion is that, no matter how small the initial amplitude is, a BH will form in AdS after a time scale ${\mathcal O({\epsilon ^{- 2}})}$. A corollary is then that linear analysis misses the essential physics of this problem; in other words, the evolution always drives the system away from the linear regime.

The central property of AdS to obtain this instability is its global structure, rather than its local geometry. This can be established by noting that a qualitatively similar behaviour is obtained by considering precisely the same dynamical system in Minkowski space enclosed in a cavity [537], see Figure 9 (right panel). Moreover, the mechanism behind the instability seems to rely on nonlinear interactions of the field that tend to shift its energy to higher frequencies and hence smaller wavelenghts. This process stops in GR since the theory has a natural cutoff: BH formation.

It has since been pointed out that collapse to BHs may not be the generic outcome of evolutions in AdS [145, 66, 274, 538, 539]. For example, in Ref. [145] “islands of stability” were discovered for which the initial data, chosen as a small perturbation of a boson star, remain in a nonlinearly stable configuration. In Ref. [586], the authors raised the possibility that some of the features of the AdS instability could also show up in asymptotically flat spacetimes in the presence of some confinement mechanism. They observed that the evolution of minimally coupled, massive scalar wavepackets in asymptotically flat spacetimes can also lead to collapse after a very large number of “bounces” off the massive effective potential barrier in a manner akin to that discovered in AdS. Similarly, in some region of the parameter space the evolution drives the system towards nonlinearly stable, asymptotically flat “oscillatons” [688, 326]. Nevertheless, for sufficiently small initial amplitudes they observe a *t*^−3/2^ decay of the initial data, characteristic of massive fields, and showing that Minkowski is nonlinearly stable. The “weakly turbulent” instability discovered in AdS is stimulating research on the topic of turbulence in GR. A full understanding of the mechanism(s) will require further studies, including collapse in non-spherically symmetric backgrounds, other forms of matter and boundary conditions, etc.

We now turn to solutions that display an instability at linear level, seen by a mode analysis, and to the use of NR techniques to follow the development of such instabilities into the nonlinear regime. One outstanding example is the Gregory-Laflamme instability of black strings already described in Section 7.2. Such black strings exist in higher dimensions, *D ≥* 5. It is expected that the same instability mechanism afflicts other higher-dimensional BHs, even with a topologically spherical horizon. A notable example are Myers-Perry BHs. In *D* ≥ 6, the subset of these solutions with a single angular momentum parameter have no analogue to the Kerr bound, i.e., a maximum angular momentum for a given mass. As the angular momentum increases, they become *ultra-spinning* BHs and their horizon becomes increasingly flattened and hence resembling the horizon of a black *p*-brane, which is subject to the Gregory-Laflamme instability [305]. It was indeed shown in Ref. [272, 271, 270], by using linear perturbation theory, that rapidly rotating Myers-Perry BHs for 7 ≤ *D ≤* 9 are unstable against axisymmetric perturbations. The nonlinear growth of this instability is unknown but an educated guess is that it may lead to a deformation of the pancake like horizon towards multiple concentric rings.

A different argument — of entropic nature — for the instability of ultra-spinning BHs against *non-axisymmetric* perturbations was given by Emparan and Myers [305]. Such type of instability has been tested in 6 ≤ *D ≤* 8 [700], but also in *D* = 5 [701, 700] — for which a slightly different argument for instability was given in [305] — by evolving a Myers-Perry BH with a non-axisymmetric bar-mode deformation, using a NR code adapted to higher dimensions. In each case sufficiently rapidly rotating BHs are found to be unstable against the bar-mode deformation. In terms of a dimensionless spin parameter *q* ≡ *a/μ*^1/(*D−*3)^, where *μ*, *a* are the standard mass and angular momentum parameters of the Myers-Perry solution, the onset values for the instability were found to be: *D* = 5, *q* = 0.87; *D* = 6, *q* = 0.74; *D = 7, q* = 0.73; *D* = 8, *q* = 0.77. We remark that the corresponding values found in [272] for the Gregory-Laflamme instability in 7 ≤ *D ≤* 9 are always larger than unity. Thus, the instability triggered by non-axisymmetric perturbations sets in for lower angular momenta than the axisymmetric Gregory-Laflamme instability. Moreover, in [700], long-term numerical evolutions have been performed to follow the nonlinear development of the instability. The central conclusion is that the unstable BHs relax to stable configurations by radiating away the excess angular momentum. These results have been confirmed for *D* = 6, 7 by a linear analysis in Ref. [273]; such linear analysis suggests that for *D* = 5, however, the single spinning Myers-Perry BH is linearly stable.^20^

**Figure 10:**
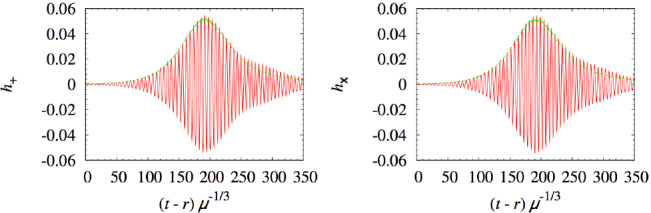
(a) and (b): + and × modes of gravitational waveform (solid curve) from an unstable six-dimensional BH with *q* = 0.801 as a function of a retarded time defined by *t* − *r*, where *r* is the coordinate distance from the center. Image reproduced with permission from [700], copyright by APS.

Results for the *D* = 6 “gravitational waveforms” *h*_+_, _×_ (see Eqs. (43) — (44) in Ref. [700] for a precise definition of these quantities) for an initial dimensionless *q* = 0.801 are shown in Figure 10. The early stage shows an exponential increase of the amplitude, after which a saturation phase is reached, and where angular momentum is being shed through GW emission. After this stage, exponential decay of the oscillations ensues. This bar-mode instability discovered — before linear perturbation analysis — in Refs. [701, 700] has recently been seen at linear level for BHs having all angular momentum parameters equal in Refs. [395, 310].

Another spacetime instability seen at linear level is the superradiant instability of rotating or charged BHs in the presence of massive fields or certain boundary conditions. This instability will be discussed in detail in the next section.

Concerning the nonlinear stability of BH solutions, the only generic statement one can produce at the moment is that hundreds of NR evolutions of binary Kerr or Schwarzschild BHs in vacuum, over the last decade, lend empirical support to the nonlinear stability of these solutions. One must remark, however, on the limitations of testing instabilities with NR simulations. For instance, fully nonlinear dynamical simulations cannot probe — at least at present — extremal Kerr BHs; they are also unable to find instabilities associated with very high harmonic indices *ℓ, m* (associated with very small scales), as well as instabilities that may grow very slowly. Concerning the first caveat, it was actually recently found by Aretakis that extremal RN and Kerr BHs are linearly unstable against scalar perturbations [42, 41, 43], an observation subsequently generalised to more general linear fields [530] and to a nonlinear analysis [563, 44]. This growth of generic initial data on extremal horizons seems to be a very specific property of extremal BHs, in particular related to the absence of a redshift effect [563], and there is no evidence a similar instability occurs for non-extremal solutions.

To conclude this section let us briefly address the stability of BH *interiors* already discussed in Section 3.2.2. The picture suggested by Israel and Poisson [621, 622] of mass inflation has been generically confirmed in a variety of toy models — i.e., not Kerr — by numerical evolutions [151, 152, 153, 393, 56, 448, 57] and also analytical arguments [233]. Other numerical/analytical studies also suggest the same holds for the realistic Kerr case [388, 386, 387, 531]. As such, the current picture is that mass inflation will drive the curvature to Planckian values, near *or* at the Cauchy horizon. The precise nature of the consequent singularity, that is, if it is space-like or light-like, is however still under debate (see, e.g., [235]).

### Superradiance and fundamental massive fields

There are several reasons to consider extensions of GR with minimally, or non-minimally coupled massive scalar fields with mass parameter *μ*_*s*_. As mentioned in Section 3.1.4, ultra-light degrees of freedom appear in the *axiverse* scenario [49, 50] and they play an important role in cosmological models and also in dark matter models. Equally important is the fact that massive scalar fields are a very simple proxy for more complex, realistic matter fields, the understanding of which in full NR might take many years to achieve.

At linearized level, the behavior of fundamental fields in the vicinities of non-rotating BHs has been studied for decades, and the main features can be summarized as follows:

(i) A prompt response at early times, whose features depend on the initial conditions. This is the counterpart to light-cone propagation in flat space.
(ii) An exponentially decaying “ringdown” phase at intermediate times, where the BH is ringing in its characteristic QNMs. Bosonic fields of mass *μ*_*s*_*ħ* introduce both an extra scale in the problem and a potential barrier at distances ∼ 1/*μ*_*s*_, thus effectively trapping fluctuations. In this case, extra modes appear which are quasi-bound states, i.e., extremely long-lived states effectively turning the BH into a quasi-hairy BH [76, 794, 280, 656, 604, 135].
(iii) At late times, the signal is dominated by a power-law fall-off, known as “late-time tail” [633, 506, 209, 491]. Tails are caused by backscattering off spacetime curvature (and a potential barrier induced by massive terms) and more generically by a failure of Huygens’ principle. In other words, radiation in curved spacetimes travels not only *on*, but *inside* the entire light cone.

**Figure 11:**
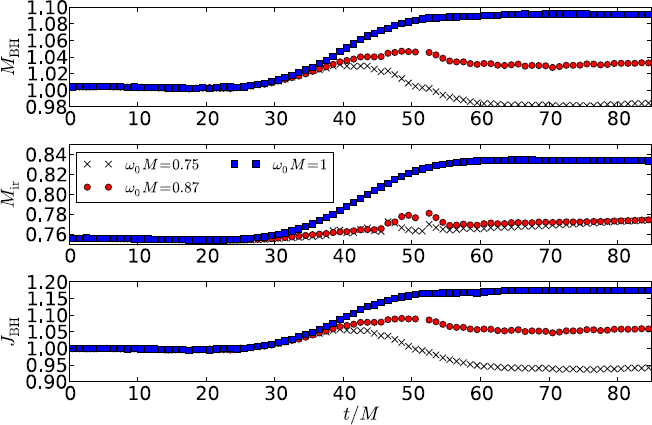
Evolution of a highly spinning BH (*a/M* = 0.99) during interaction with different frequency GW packets, each with initial mass ≈ 0.1 *M*. Shown (in units where *M* = 1) are the mass, irreducible mass, and angular momentum of the BH as inferred from AH properties. Image reproduced with permission from [289], copyright by APS.

When the BH is rotating, a novel effect can be triggered: *superradiance* [827, 828, 83, 164]. Superradiance consists of energy extraction from rotating BHs, and a transfer of this energy to the interacting field [164]. For a monochromatic wave of frequency *ω*, the condition for superradiance is [827, 828, 83, 164]

173 $$\omega < m{\Omega _H},$$

where *m* is the azimuthal harmonic index and Ω_*H*_ is the angular velocity of the BH horizon. If, in addition, the field is massive, a “BH bomb-type” mechanism can ensue [241, 268, 843, 171] leading to an instability of the spacetime and the growth of a scalar condensate outside the BH horizon [605, 815, 794, 280, 588, 164]. The rich phenomenology of scenarios where fundamental fields couple to gravity motivated recent work on the subject, where full nonlinear evolutions are performed [588, 289, 410, 92]. East et al. [289] have performed nonlinear scattering experiments, solving the field equations in the generalized harmonic formulation, and constructing initial data representing a BH with dimensionless spin *a/M* = 0.99, and an incoming quadrupolar GW packet. Their results are summarized in Figure 11, for three different wavepacket frequencies, *Mω* = 0.75, 0.87, 1 (note that only the first is superradiant according to condition (173)). The wavepackets carry roughly 10% of the spacetime’s total mass. These results confirm that low frequency radiation does extract mass and spin from the BH (both the mass *M*_BH_ and spin Jbh of the BH decrease for the superradiant wavepacket with *Mω* = 0.75), and that nonlinear results agree quantitatively with linear predictions for small wavepacket amplitudes [745]. To summarize, superradiance is confirmed at full nonlinear level, providing a rigorous framework for the complex dynamics that are thought to arise for massive fields around rotating BHs.

Self-interacting scalars can give rise to stable or very long-lived configurations. For example, self-interacting complex scalar fields can form *boson stars* for which the scalar field has an oscillatory nature, but the metric is stationary [458, 685, 516, 533]. Real-valued scalars can form oscillating solitons or “oscillatons”, long-lived configurations where both the scalar field and the metric are time-dependent [688, 689, 594, 586]. Dynamical boson star configurations were studied by several authors [516], with focus on boson star collisions with different velocities and impact parameters [597, 599, 216]. These are important for tests of the hoop and cosmic censorship conjectures, and were reviewed briefly in Sections 7.2 and 7.3. For a thorough discussion and overview of results on dynamical boson stars we refer the reader to the *Living Reviews* article by Liebling and Palenzuela [516].

**Figure 12:**
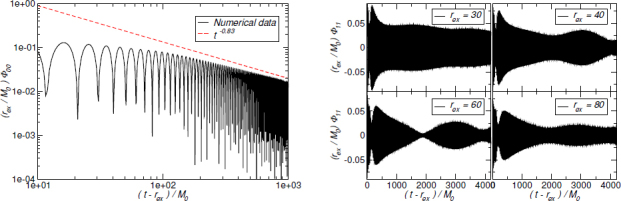
Massive scalar field (nonlinear) evolution of the spacetime of an initially non-rotating BH, with *Mμ* = 0.29. *Left panel*: Evolution of a spherically symmetric *l = m = 0* scalar waveform, measured at *r*_ex_ = 40 *M*, with *M* the initial BH mass. In addition to the numerical data (black solid curve) we show a fit to the late-time tail (red dashed curve) with *t*^−0.83^, in excellent agreement with linearized analysis. *Right panel*: The dipole signal resulting from the evolution of an *l* = *m* = 1 massive scalar field around a non-rotating BH. The waveforms, extracted at different radii *r*_ex_ exhibit pronounced beating patterns caused by interference of different overtones. The critical feature is however, that these are extremely long-lived configurations. Image reproduced with permission from [588], copyright by APS.

The first steps towards understanding the nonlinear interaction between massive fields and BHs were taken by Okawa et al. [588, 585], who found new ways to prescribe, and evolve, constraint-satisfying initial data, analytically or semi-analytically, for minimally coupled self-interacting scalar fields [588, 586]. This construction was reviewed in Section 6.3. In Ref. [588], the authors used this procedure to generate initial data and to evolve wavepackets of arbitrary angular shape in the vicinity of rotating BHs. Their results are summarized in Figure 12. Spherically symmetric initial data for massless fields reproduce previous results in the literature [382], and lead to power-law tails of integer index. The mass term adds an extra scale and a barrier at large distances, resulting in characteristic late-time tails of massive fields.

Full nonlinear results from Ref. [588] are reproduced in Figure 12, and agree with linearized predictions. Higher multipoles “feel” the centrifugal barrier close to the light ring which, together with the mass barrier at large distances, provides a confining mechanism and gives rise to almost stationary configurations, shown in the right panel of Figure 12. The beating patterns are a consequence of the excitation of different overtones with similar ringing frequency [794, 588]. These “scalar condensates” are extremely long-lived and can, under some circumstances, be considered as adding hair to the BH. They are not however really stationary: the changing quadrupole moment of the “scalar cloud” triggers the simultaneous release of gravitational radiation [588, 816, 585]. In fact, gravitational radiation is one of the most important effects not captured by linearized calculations. These nontrivial results extend to higher multipoles, which display an even more complex behavior [588, 585].

Although only a first step towards understanding the physics of fundamental fields in strong-field gravity, these results are encouraging. We expect that with more robust codes and longer simulations one will be able to fully explore the field, in particular, the following features.

- Superradiant instability and its saturation. The timescales probed in current nonlinear simulations are still not sufficient to unequivocally observe superradiance with test scalar fields. The main reason for this is the feebleness of such instabilities: for scalar fields they have, at best, an instability timescale of order 10^7^ *M* for carefully tuned scalar field mass. However, current long-term simulations are able to extract GWs induced by the scalar cloud [588].
  The biggest challenge ahead is to perform simulations which are accurate enough and last long enough to observe the scalar-instability growth *and* its subsequent saturation by GW emission. This will allow GW templates for this mechanism to finally be released.
  Due to their simplicity, scalar fields are a natural candidate to carry on this program, but they are not the only one. Massive vector fields, which are known to have amplification factors one order of magnitude larger, give rise to stronger superradiant instabilities, and might also be a good candidate to finally observe superradiant instabilities at the nonlinear level. We note that the development of the superradiant instability may, in some special cases, lead to a truly asymptotically flat, hairy BH solution of the type recently discussed in [422].
- Turbulence of massive fields in strong gravity. Linearized results indicate that the development of superradiant instabilities leaves behind a scalar cloud with scalar particles of frequency *ω* ∼ *m*Ω, in a nearly stationary state. This system may therefore be prone to turbulent effects, where nonlinear terms may play an important role. One intriguing aspect of these setups is the possibility of having gravitational turbulence or collapse on sufficiently large timescales. Such effects were recently observed in “closed” systems where scalar fields are forced to interact gravitationally for long times [108, 537, 145]. It is plausible that quasibound states are also prone to such effects, but in asymptotically flat spacetimes.
- Floating orbits. Our discussion until now has focused on minimally coupled fundamental fields. If couplings to matter exist, new effects are possible: a small object (for example, a star), orbiting a rotating, supermassive BH might be able to extract energy and angular momentum from the BH and convert it to gravitational radiation. For this to happen, the object would effectively stall at a superradiant orbit, with a Newtonian frequency Ω = 2*μ*_*s*_ for the dominant quadrupolar emission. These are called *floating orbits*, and were verified at linearized level [165, 164].^21^ Nonlinear evolutions of systems on floating orbits are extremely challenging on account of all the different extreme scales involved.
- Superradiant instabilities in AdS. The mechanism behind superradiant instabilities relies on amplification close to the horizon and reflection by a barrier at large distances. Asymptotically AdS spacetimes provide an infinite-height barrier, ideal for the instability to develop [171, 166, 172, 755, 169].
  Because in these backgrounds there is no dissipation at infinity, it is both possible and likely that new, non-symmetric final states arise as a consequence of the superradiant instability [172, 168, 514, 275]. Following the instability growth and its final state remains a challenge for NR in asymptotically AdS spacetimes.
- Superradiant instabilities of charged BHs. Superradiant amplification of *charged* bosonic fields can occur in the background of charged BHs, in quite a similar fashion to the rotating case above, as long as the frequency of the impinging wave *ω* obeys
  174 $$\omega < q{\Phi _H},$$
  where *q* is the charge of the field and Φ_*H*_ is the electric potential on the BH horizon. In this case both charge and Coulomb energy are extracted from the BH in a way compatible with the first and second law of BH thermodynamics [83]. In order to have a recurrent scattering, and hence, an instability, it is not enough, however, to add a mass term to the field [339, 432, 430, 261, 671]; but an instability occurs either by imposing a mirror like boundary condition at some distance from the BH (i.e., a boxed BH) or by considering an asymptotically AdS spacetime. In Refs. [421, 262] it has been established, through both a frequency and a time domain analysis, that the time scales for the development of the instability for boxed BHs can be made much smaller than for rotating BHs (in fact, arbitrarily small [431]). Together with the fact that even *s*-waves, i.e., *ℓ* = 0 modes, can trigger the instability in charged BHs, makes the numerical study of the nonlinear development of this type of superradiant instabilities particularly promising. One should be aware, however, that there may be qualitative differences in both the development and end-point of superradiant instabilities in different setups. For instance, for AdS and boxed BHs, the end-point is likely a hairy BH, such as those constructed in Ref. [275] (for rotating BHs), since the scalar field cannot be dissipated anywhere. This applies to both charged and rotating backgrounds. By contrast, this does not apply to asymptotically flat spacetimes, wherein rotating (but not charged) superradiance instability may occur. It is then an open question if the system approaches a hairy BH — of the type constructed in [422] — or if the field is completely radiated/absorbed by the BH. Concerning the development of the instability, an important difference between the charged and the rotating cases may arise from the fact that a similar role, in Eqs. (173) and (174), is played by the field’s azimuthal quantum number *m* and the field charge *q*; but whereas the former may change in a nonlinear evolution, the latter is conserved [169].

### High-energy collisions

Applications of NR to collisions of BHs or compact matter sources near the speed of light are largely motivated by probing GR in its most violent regime and by the modelling of BH formation in TeV gravity scenarios. The most important questions that arise in these contexts can be summarized as follows.

- Does cosmic censorship still apply under the extreme conditions of collisions near the speed of light? As has already been discussed in Section 7.2, numerical simulations of these collisions in four dimensions have so far identified horizon formation in agreement with the censorship conjecture. The results of higher-dimensional simulations are still not fully understood, cf. Section 7.2.
- Do NR simulations of high-energy particle collisions provide evidence supporting the validity of the hoop conjecture? As discussed in Section 7.3, NR results have so far confirmed the hoop conjecture.
- In collisions near the speed of light, the energy mostly consists of the kinetic energy of the colliding particles such that their internal structure should be negligible for the collision dynamics. Furthermore, the gravitational field of a particle moving at the speed of light is non-vanishing only near the particle’s worldline [16], suggesting that the gravitational interaction in high-energy collisions should be dominant at the instant of collision and engulfed inside the horizon that forms. This conjecture has sometimes been summarized by the statement that “matter does not matter” [216], and is related to the hoop conjecture discussed above. Do NR simulations of generic high-energy collisions of compact objects support this argument in the classical regime, i.e., does the modelling of the colliding objects as point particles (and, in particular, as BHs) provide an accurate description of the dynamics?
- Assuming that the previous question is answered in the affirmative, what is the scattering threshold for BH formation? This corresponds to determining the threshold impact parameter *b*_scat_ that separates collisions resulting in the formation of a single BH (*b* < *b*_scat_) from scattering encounters (*b* > *b*_scat_), as a function of the number of spacetime dimensions *D* and the collision velocity *v* in the center-of-mass frame or boost parameter $\gamma = 1/\sqrt {1 - {v^2}}$.
- How much energy and momentum is lost in the form of GWs during the collision? By conversion of energy and momentum, the GW emission determines the mass and spin of the BH (if formed) as a function of the spacetime dimension *D*, scattering parameter b, and boost factor *γ* of the collision. Collisions near the speed of light are also intriguing events to probe the extremes of GR; in particular what is the maximum radiation that can be extracted from any collision and does the luminosity approach Dyson’s limit d*E/* d*t* ≲ 1 [157]? (See discussion in Section 3.2.3 about this limit.)

These issues are presently rather well understood through NR simulations in *D* = 4 spacetime dimensions but remain largely unanswered for the important cases *D* ≥ 5.

The relevance of the internal structure of the colliding bodies has been studied in Ref. [716], comparing the GW emission and scattering threshold in high-energy collisions of rotating and non-rotating BHs in *D* = 4. The BH spins of the rotating configurations are either aligned or anti-aligned with the orbital angular momentum corresponding to the so-called *hang-up* and *anti-hang-up* cases which were found to have particularly strong effects on the dynamics in quasi-circular BH binary inspirals [160]. In high-energy collisions, however, this (anti-)hang-up effect disappears; the GW emission as well as the scattering threshold are essentially independent of the BH spin at large collision velocities (cf. Figure 15 which will be discussed in more detail further below). These findings suggest that ultra-relativistic collisions are indeed well modelled by colliding point-particles or BHs in GR. In the center-of-mass frame, and assuming that the two particles have equal mass, the collisions are characterized by three parameters. (i) The number *D* of spacetime dimensions, (ii) the Lorentz factor *γ* or, equivalently, the collision velocity *υ*, and (iii) the impact parameter *b = L/P*, where *L* and *P* are the initial orbital angular momentum and the linear momentum of either BH in the center-of-mass frame.

The simplest set of configurations consists of head-on collisions with *b* = 0 in *D* = 4 dimensions and was analysed in Ref. [719] varying the boost parameter in the range 1.07 ≤ γ ≤ 3. In agreement with the cosmic censorship conjecture, these collisions always result in the formation of a single BH that settles into a stationary configuration through quasi-normal ringdown. The total energy radiated in the form of GWs is well modelled by the following functional form predicted by Smarr’s [707] zero-frequency limit (see Section 5.3)

175 $${E \over M} = {E_\infty}\left({{{1 + 2{\gamma ^2}} \over {2{\gamma ^2}}} + {{(1 - 4{\gamma ^2})\,\log (\gamma + \sqrt {{\gamma ^2} - 1})} \over {2{\gamma ^3}\sqrt {{\gamma ^2} - 1}}}} \right),$$

where *E*_*∞*_ is a free parameter that corresponds to the fraction of energy radiated in the limit γ→∞. Fitting the numerical results with Eq. (175) yields *E*_*∞*_ = 14 ± 3 % which is about half of Penrose’s upper limit [611, 286]. Observe the good agreement with the second order result in Eq. (38) as discussed in Section 5.4.

**Figure 13:**
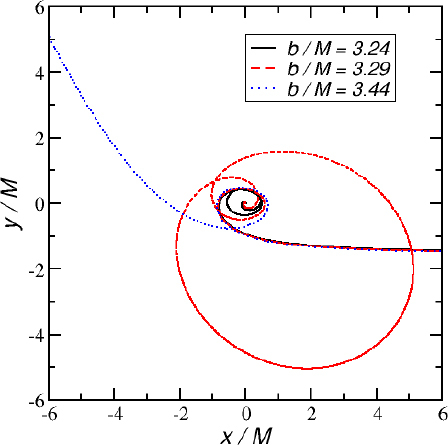
BH trajectories in grazing collisions for γ = 1.520 and three values of the impact parameter corresponding to the regime of prompt merger (solid, black curve), of delayed merger (dashed, red curve), and scattering (dotted, blue curve). Note that for each case, the trajectory of one BH is shown only; the other BH’s location is given by symmetry across the origin.

**Figure 14:**
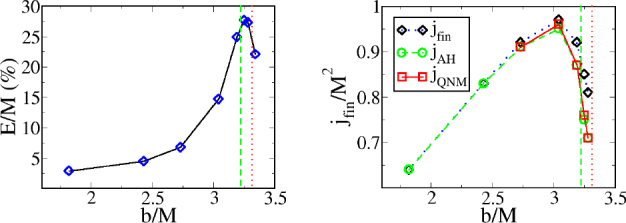
Total energy radiated in GWs (left panel) and final dimensionless spin of the merged BH (right panel) as a function of impact parameter *b* for the same grazing collisions with γ = 1.520. The vertical dashed (green) and dash-dotted (red) lines mark b* and *b*_scat_, respectively. Image reproduced with permission from [720], copyright by APS.

Grazing collisions in four dimensions represent two-parameter studies, where the boost factor 7 and the impact parameter *b* are varied, and have been investigated in Refs. [697, 720]. At fixed Lorentz boost, such grazing collisions exhibit three distinct regimes as the impact parameter is increased from the head-on limit *b* = 0: (i) prompt mergers, (ii) delayed mergers, and (iii) the scattering regime where no common horizon forms. These regimes are marked by two special values of the impact parameter b, the scattering threshold *b*_scat_ that we have already mentioned above and the threshold of immediate merger b*. This threshold has been identified in numerical BH simulations by Pretorius & Khurana [632] as marking the onset of a regime where the two BHs *whirl* around each other prior to merging or scattering off for a number of orbits proportional to log | *b* − b*|; see also [413, 355]. This *zoom-whirl-like* behaviour has also been identified in high-energy grazing collisions in [720]. The three different regimes are illustrated in Figure 13 which shows the BH trajectories for γ = 1.520 and *b/M* = 3.24, 3.29 and 3.45. For this boost factor, the thresholds are given by b*/M ≈ 3.25 and *b*_scat_/*M* ≈ 3.35. For impact parameters close to the threshold values b* and *b*_scat_, grazing collisions can generate enormous amounts of GWs. This is shown in the left panel of Figure 14. Starting from *E/M ≈* 2.2% in the head-on limit b = 0, the radiated energy increases by more than an order of magnitude to ≳ 25% for b* < *b* < *b*_scat_. These simulations can also result in BHs spinning close to the extremal Kerr limit as is shown in the right panel of the figure which plots the dimensionless final spin as a function of b. By fitting their numerical results, Shibata et al. [697] have found an empirical relation for the scattering threshold given by

176 $${{{b_{{\rm{scat}}}}} \over M} = {{2.50 \pm 0.05} \over v}\,.$$

for such a value of the impact parameter, they observe that the dimensionless final spin of the merged BH is given by *J*_fin_ = (0.6 ± 0.1) *J*, where *J/M*^2^ = 1.25 ± 0.03 is the initial angular momentum of the system.

**Figure 15:**
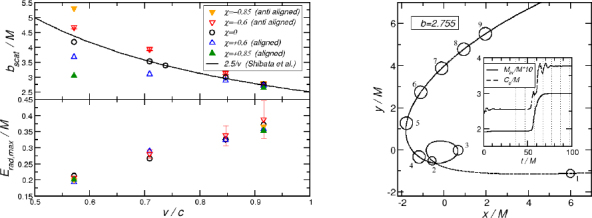
*Left panels*: Scattering threshold (upper panel) and maximum radiated energy (lower panel) as a function of *υ*. Colored “triangle” symbols pointing up and down refer to the aligned and antialigned cases, respectively. Black “circle” symbols represent the thresholds for the nonspinning configurations. *Right panel*: Trajectory of one BH for a delayed merger configuration with anti-aligned spins *j* = 0.65. The circles represent the BH location at equidistant intervals Δ*t* = 10 *M* corresponding to the vertical lines in the inset that shows the equatorial circumference of the BH’s AH as a function of time.

Grazing collisions of spinning and non-spinning BHs have been compared in Ref. [716]. The initial configurations for these simulations have been chosen with γ factors up to 2.49 and equal spin for both BHs of dimensionless magnitude *χ* = 0.85 and 0.65 aligned or anti-aligned with the orbital angular momentum *L*. This set has been complemented with collisions of non-spinning BHs covering the same range in γ. The scattering threshold and the energy radiated in GWs in these simulations are shown in the left panel of Figure 15. As expected from the hang-up effect, aligned (anti-aligned) spins result in a smaller (larger) value of the scattering threshold *b*_scat_ at low collision speeds. At velocities above ∼ 80% of the speed of light, however, this effect is washed out and, in agreement with the matter-does-not-matter conjecture mentioned above, the collision dynamics are barely affected by the BH spins. Furthermore, the scattering threshold determined for non-spinning BHs agrees very well with the formula (176). As demonstrated in the bottom panel of the figure, the radiated energy is barely affected by the BH spin even in the mildly relativistic regime. The simulations also suggest an upper limit of the fraction of kinetic energy that can be converted into GWs. Extrapolation of the data points in Figure 15 to *υ* = 1 predicts that at most about half of the total energy can be dissipated in GWs in any four dimensional collision. The other half, instead, ends up as rest mass inside the common horizon formed in merging configurations or is absorbed by the individual BHs during the close encounter in scattering processes (the result of this extrapolation is consistent with the calculation in Ref. [376]). This is illustrated in the right panel of Figure 15, where the trajectory of one BH in a delayed merger configuration with anti-aligned spins is shown. The circles, with radius proportional to the horizon mass, represent the BH location at intervals Δ*t* = 10 *M*. During the close encounter, (i) the BH grows in size due to absorption of gravitational energy and (ii) slows down considerably.

Collisions of BHs with electric charge have been simulated by Zilhão et al. [838, 839]. For the special case of BHs with equal charge-to-mass ratio *Q/M* and initially at rest, constraint-satisfying initial data are available in closed analytic form. The electromagnetic wave signal generated in these head-on collisions reveals three regimes similar to the pattern known for the GW signal, (i) an infall phase prior to formation of a common horizon, (ii) the nonlinear merger phase where the wave emission reaches its maximum and (iii) the quasi-normal ringdown. As the charge-to-mass ratio is increased towards *Q/M* ≲ 1, the emitted GW energy decreases by about 3 orders of magnitude while the electromagnetic wave energy reaches a maximum at *Q/M* ≈ 0.6, and drops towards 0 in both the uncharged and the extreme limit. This behaviour of the radiated energies is expected because of the decelerating effect of the repulsive electric force between equally charged BHs. For opposite electric charges, on the other hand, the larger collision velocity results in an increased amount of GWs and electromagnetic radiation [839].

An extended study of BH collisions using various analytic approximation techniques including geodesic calculations and the ZFL has been presented in Berti et al. [93]; see also [94] for a first exploration in higher dimensions. Weak scattering of BHs in *D* = 4, which means large scattering parameters *b*/*M* ∼ 10, and for velocities υ ≈ 0.2, has been studied by Damour et al. [245] using NR as well as PN and EOB calculations. Whereas PN calculations start deviating significantly from the NR results for *b*/*M* ≲ 10, the NR calibrated EOB model yields good agreement in the scattering angle throughout the weak scattering regime.

BH collisions in *D* ≥ 5 spacetime dimensions are not as well understood as their four-dimensional counterparts. This is largely a consequence of the fact that NR in higher dimensions is not yet that robust and suffers more strongly from numerical instabilities. Such complications in the higher-dimensional numerics do not appear to cause similar problems in the construction of constraint satisfying initial data. The spectral elliptic solver originally developed by Ansorg et al. [39] for *D* = 4 has been successfully generalized to higher *D* in Ref. [836] and provides solutions with comparable accuracy as in *D* = 4.

**Figure 16:**
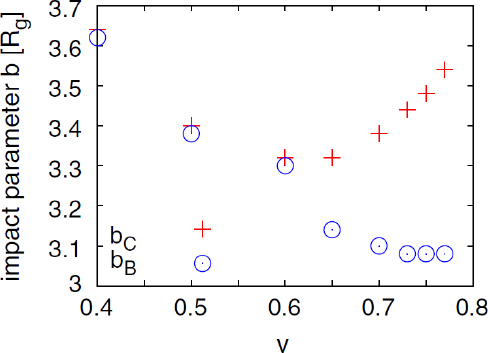
The (red) plus and (blue) circle symbols mark scattering and merging BH configurations, respectively, in the *b −* υ plane of impact parameter and collision speed, for *D* = 5 spacetime dimensions.

A systematic exploration of the scattering threshold in *D* = 5 dimensions has been performed in Ref. [587]. By superposing non-rotating, boosted single BH initial data, they have evolved grazing collisions up to υ ≲ 0.8. Their results are summarized in Figure 16, where scattering (merging) BH collisions are marked by “plus” and “circle” symbols in the plane spanned by the collision velocity *υ* and the impact parameter *b*. The simulations show a decrease of the scattering threshold at increasing velocity up to *υ ≈* 0.6, similar to the *D* = 4 case in the upper left panel of Figure 15. At larger *υ*, the threshold cannot yet be determined because simulations with near critical impact parameter become numerically unstable. By monitoring the Kretschmann scalar at the point of symmetry between the two BHs, a large curvature regime was furthermore identified in [587], as discussed in Section 7.2.

**Figure 17:**
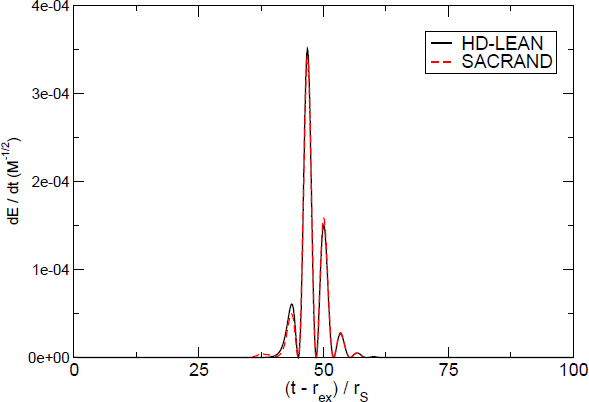
Energy fluxes for head-on collisions of two BHs in *D* = 5 spacetime dimensions, obtained with two different codes, HD-Lean [841, 797] (solid black line) and SaoraND [820, 587] (red dashed line). The BHs start off at an initial coordinate separation *d/R*_*s*_ = 6.47. Image adapted from [796].

The GW emission in BH mergers in *D* > 4 has so far only been studied for collisions starting from rest. In Refs. [797, 793, 796] the wave signal was extracted using the Kodama-Ishibashi formalism discussed in Section 6.7.2; the GW emission contains about 0.09%, 0.08% of the center-of-mass energy for equal-mass binaries in *D* = 5, 6 respectively (note that in *D* = 4 only 0.055% of the center-of-mass-eanergy goes into GWs), and decreases with the mass ratio. The dependency of the radiated energy and momentum on the mass ratio is well modelled by point particle calculations [94]. A comparison of the predicted GW emission in higher-dimensional collisions using two different numerical codes with different formulations of the Einstein equations, namely those discussed in Sections 6.2.1 and 6.2.2, has been presented in Ref. [796]. The predictions from the two codes using respectively the Kodama-Ishibashi formalism (cf. Section 6.7.2) and a direct extraction through the metric components (cf. Section IV B 1 in [700]) agree within numerical uncertainties [796]. This result, illustrated in Figure 17, represents an important validation of both the numerical evolution techniques and the diagnostics of the simulations, along with the first estimate of the emitted energy for head-on collisions in *D* = 6.

The main challenges for future numerical work in the field of high-energy collisions are rather evident. For applications in the analysis of experimental data in the context of TeV gravity scenarios (cf. Section 3.3.2), it will be vital to generalize the results obtained in four dimensions to *D* ≥ 5. Furthermore, it is currently not known whether the impact of electric charge on the collision dynamics becomes negligible at high velocities, as suggested by the matter-does-not-matter conjecture, and as is the case for the BH spin.

### Alternative theories

As discussed in Section 6.1.7, one of the most straightforward *extensions* of Einstein’s theory is obtained by the addition of minimally coupled scalar fields. When the scalar couples to the Ricci scalar however, one gets a modification of Einstein gravity, called scalar-tensor theory. In vacuum, scalar-tensor theories are described by the generic action in Eq. (5), where *R* is the Ricci scalar associated to the metric *g*_*μν*_, and *F*(*ϕ*), *Z*(*ϕ*) and *U*(*ϕ*) are arbitrary functions (see e.g., [92, 824] and references therein). The matter fields minimally coupled to *g*_*μν*_ are collectively denoted by Ψ_*m*_. This form of the action corresponds to the choice of the so-called “Jordan frame”, where the matter fields Ψ_*m*_ obey the equivalence principle. For *F = ϕ, Z = ω*_BD_/*ϕ*, *U = 0*, the action (5) reduces to the standard Brans-Dicke theory.

The equations of motion derived from the action (5) are second-order and the theory admits a well-posed initial-value problem [670]. These facts turn scalar-theories into an attractive alternative to Einstein’s equations, embodying at least some of the physics one expects from an ultimate theory of gravity, and have been a major driving force behind the efforts to understand scalar-tensor theories from a NR point of view [696, 679, 680, 582, 410, 92, 73, 670]. In fact, scalar-tensor theories remain the only alternative theory to date where full nonlinear dynamical evolutions of BH spacetimes have been performed.

Scalar-tensor theories can be recast in such a way as to be formally equivalent, in vacuum, to GR with a *minimally coupled* scalar field, i.e., to the theory described previously in Section 6.1.7. This greatly reduces the amount of work necessary to extend NR to these setups. The explicit transformations that recast the previous action in the “Einstein frame” are [244]

177 $$g_{\mu \nu}^E = F(\phi){g_{\mu \nu}},\quad V = {U \over {16\pi F{{(\phi)}^2}}},$$

178 $$\varphi (\phi) = {1 \over {\sqrt {4\pi G}}}\int {{\rm{d}}\phi} {\left[ {{3 \over 4}{{{F\prime}{{(\phi)}^2}} \over {F{{(\phi)}^2}}} + {{4\pi GZ(\phi)} \over {F(\phi)}}} \right]^{1/2}}.$$

The Einstein-frame action is then

179 $$S = \int {{{\rm{d}}^4}} x\sqrt {- g} \left({{{{R^E}} \over {16\pi G}} - {1 \over 2}{g^{E,\,\mu \nu}}{\partial _\mu}{\varphi ^ \ast}{\partial _\nu}\varphi - V(\varphi)} \right) + {S_{{\rm{matter}}}}({\Psi _m};\,{F^{- 1}}g_{\mu \nu}^E)\,,$$

which is the action for a minimally coupled field (77) enlarged to allow for a generic self-interaction potential (which could include the mass term). The label *E* denotes quantities constructed from the Einstein-frame metric $g_{\mu \nu}^E$. In the Einstein frame the scalar field is minimally coupled to gravity, but any matter field Ψ_*m*_ is coupled to the metric ${F^{- 1}}g_{\mu \nu}^E$.

In vacuum, this action leads to the following equations of motion:

180 $$R_{\mu \nu}^E - {1 \over 2}g_{\mu \nu}^E{R^E} - 8\pi {T_{\mu \nu}} = 0,$$

181 $${\nabla ^\mu}{\nabla _\mu}\varphi - V{\prime}(\varphi) = 0,$$

where the energy-momentum tensor of the scalar field is determined by

182 $${T_{\mu \nu}} = - {1 \over 2}g_{\mu \nu}^E({\partial _\lambda}{\varphi ^{\ast}}{\partial ^\lambda}\varphi) - g_{\mu \nu}^EV(\varphi) + {1 \over 2}({\partial _\mu}{\varphi ^{\ast}}{\partial _\nu}\varphi + {\partial _\mu}\varphi {\partial _\nu}{\varphi ^{\ast}}).$$

In summary, the study of scalar-tensor theories of gravity can be directly translated, in vacuum, to the study of minimally coupled scalar fields. For a trivial potential *V* = const, the equations of motion in the Einstein frame (180) — (181) admit GR (with _*ϕ*_ = const) as a solution. Because stationary BH spacetimes in GR are stable, i.e., any scalar fluctuations die away rather quickly, the dynamical evolution of vacuum BHs is expected to be the same as in GR. This conclusion relies on hand-waving stability arguments, but was verified to be true to first PN order by Will and Zaglauer [787], at 2.5 PN order by Mirshekari and Will [549] and to all orders in the point particle limit in Ref. [824].

**Figure 18:**
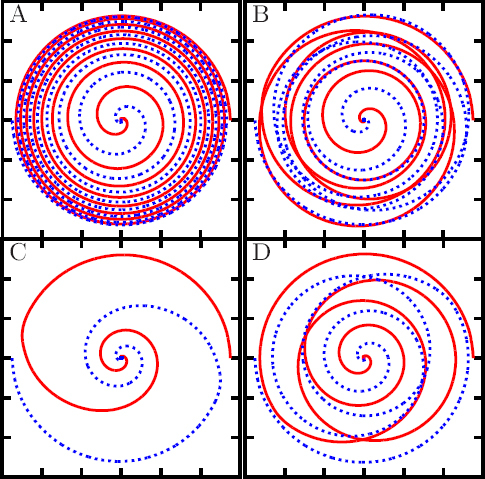
Trajectories of BHs immersed in a scalar field bubble of different amplitudes. The BH binary consists of initially non-spinning, equal-mass BHs in quasi-circular orbit, initially separated by 11 *M*, where *M* is the mass of the binary system. The scalar field bubble surrounding the binary has a radius *r*_o_ = 120 *M* and thickness *σ* = 8 *M*. Panels *A,B,C* correspond to *φ*_0_ = 0(GR), 1/80, 1/40 and a zero potential amplitude λ. Panel *D* corresponds to *φ*_0_ = 1/80,4πλ = 10^3^ *M*^2^. Image reproduced with permission from [410], copyright by IOP. All rights reserved.

**Figure 19:**
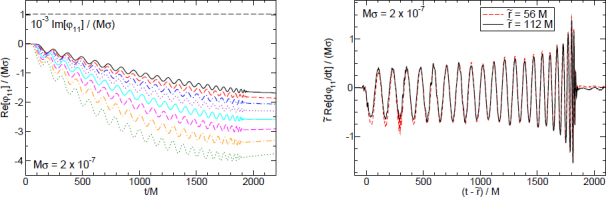
Numerical results for a BH binary inspiralling in a scalar-field gradient *Mσ* = 10^−*7*^*. Left panel*: dependence of the various components of the scalar radiation Re(_ϕ11_)/(*Mσ*) on the extraction radius (top to bottom: 112 *M* to 56 *M* in equidistant steps). The dashed line corresponds instead to 10^−3^ Im(_ϕ11_)/(*M*σ) at the largest extraction radius. This is the dominant mode and corresponds to the fixed-gradient boundary condition, along the *z*-direction, at large distances. *Right panel*: time-derivative of the scalar field at the largest and smallest extraction radii, rescaled by radius and shifted in time. Notice how the waveforms show a clean and typical merger pattern, and that they overlap showing that the field scales to good approximation as $1/\tilde r$. Image reproduced with permission from [92], copyright by APS.

**Figure 20:**
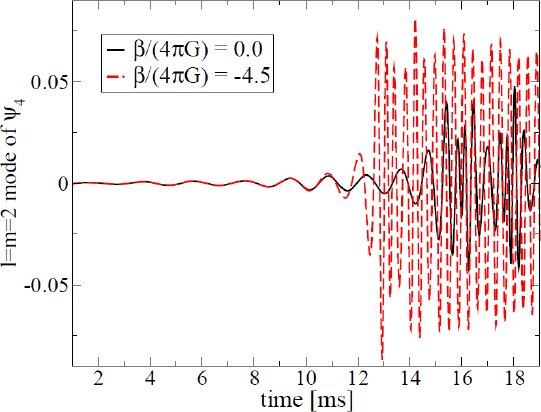
The dominant quadrupolar component of the gravitational *ψ*_4_ scalar for an equal-mass, non-spinning NS binary with individual baryon masses of 1.625 *M*_⊙_. The solid (black) curve refers to GR, and the dashed (red) curve to a scalar-tensor theory with *β*/(4*π*) = −4.5, *ϕo* = 10^−5^. Image reproduced with permission from [73], copyright by APS.

Thus, at least one of the following three ingredients are necessary to generate interesting dynamics in scalar-tensor theories:

- Nontrivial potential *V* and initial conditions. Healy et al. studied an equal-mass BH binary in an inflation-inspired potential $V = \lambda {({\varphi ^2} - \varphi _0^2)^2}/8$ with nontrivial initial conditions on the scalar given by φ = φ_0_ tanh (*r* − *r*_0_)/*σ* [410]. This setup is expected to cause deviations in the dynamics of the inspiralling binary, because the binary is now accreting scalar field energy. The larger the initial amplitude of the field, the larger those deviations are expected to be. This is summarized in Figure 18, where the BH positions are shown as a function of time for varying initial scalar amplitude.
- Nontrivial boundary conditions. As discussed, GR is recovered for constant scalar fields. For nontrivial time-dependent boundary conditions or background scalar fields, however, nontrivial results show up. These boundary conditions could mimic cosmological scenarios or dark matter profiles in galaxies [434, 92]. Reference [92] modelled a BH binary evolving nonlinearly in a constant-gradient scalar field. The scalar-field gradient induces scalar charge on the BHs, and the accelerated motion of each BH in the binary generates scalar radiation at large distances, as summarized in Figure 19.
  The scalar-signal at large distances, shown in the right panel of Figure 19, mimics the inspiral, merger and ringdown stages in the GW signal of an inspiralling BH binary.
- Matter. When matter is present, new effects (due to the coupling of matter to the effective metric ${F^{- 1}}g_{\mu \nu}^E$ can dominate the dynamics and wave emission. For example, it has been shown that, for $\beta \equiv \partial _\varphi ^2(\ln F(\varphi))\underset{\sim}{<} - 4$, NSs can “spontaneously scalarize,” i.e., for sufficiently large compactnesses the GR solution is unstable. The stable branch has a nonzero expectation value for the scalar field [242].
  Scalarized matter offers a rich new phenomenology. For example, the dynamics and GW emission of scalarized NSs can be appreciably different (for given coupling function *F*^−1^(*ϕ*)) from the corresponding GR quantities, as shown by Palenzuela et al. [73, 596] and summarized in Figure 20. Strong-field gravity can even *induce* dynamical scalarization of otherwise GR stars during inspiral, offering new ways to constrain such theories [73, 596].

The application of NR methods to the understanding of alternative theories of gravity and tests of GR is still in its infancy. Among various possible directions, we point out the following.

- Understanding the well posedness of some theory, in particular those having some motivation from fundamental physics, as for example Einstein-Dilaton-Gauss-Bonnet and Dynamical Chern-Simons gravity [602, 27]. A study on the well posedness of the latter has recently been presented in Ref. [263].
- Building initial data describing interesting setups for such theories. Unless the theory admits particularly simple analytic solutions, it is likely that initial data construction will also have to be done numerically. Apart from noteworthy exceptions, such as Gauss-Bonnet gravity in higher dimensions [814], initial data have hardly been considered in the literature.

Once well-posedness is established and initial data are constructed, NR evolutions will help us understanding how these theories behave in the nonlinear regime.

### Holography

Holography provides a fascinating new source of problems for NR. As such, in recent years, a number of numerical frameworks have been explored in asymptotically AdS spacetimes, as to face the various pressing questions raised within the holographic correspondence, cf. Section 3.3.1. At the moment of writing, no general purpose code has been reported, comparable to existing codes in asymptotically flat spacetime, which can evolve, say, BH binaries with essentially arbitrary masses, spins and momenta. Progress has occured in specific directions to address specific issues. We shall now review some of these developments emphasizing the gravity side of the problems.

**Figure 21:**
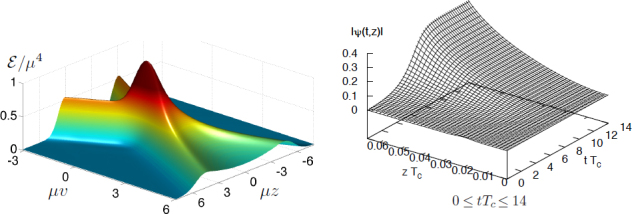
*Left panel*: Collision of two shock waves in AdS5. The energy density *ℰ*/*μ*^4^ is represented as a function of an (advanced) time coordinate *υ* and a longitudinal coordinate *z. μ* defines the amplitude of the waves. *Right panel*: Evolution of the scalar field in an unstable RN-AdS BH. *z* is a radial coordinate and the AdS boundary is at *z* = 0. Due to the instability of the BH, the scalar density grows exponentially for 0 < *tT*_*c*_ ≲ 6. Then, the scalar density approaches some static function. Images reproduced with permission from (left) [207], copyright by APS and (right) [562], copyright by SISSA.

As mentioned in Section 3.3.1 an important problem in the physics of heavy ion collisions is to understand the “early thermalization problem”. In Ref. [207], the gauge/gravity duality was used to address this issue. On the gravity side, the problem at hand was to study a head-on collision of two shock waves in asymptotically AdS5 spacetime. The numerical scheme was to perfom a null (characteristic) evolution. By choosing a specific metric ansatz, it was possible to unveil in the nonlinear Einstein equations a nested linear structure: the equations can be integrated as linear ordinary differential equations if an appropriate sequence is chosen. The AdS boundary condition was implemented by an adequate radial expansion near the boundary and the initial data consisted of two well-separated planar shocks, with finite thickness and energy density, moving toward each other. In this setup an AH is always found (even before the collision) and excision was performed by restricting the computational domain to start at this horizon. The evolution of the two shock waves is displayed in Figure 21 (left panel). By following the evolution and using the gauge/gravity dictionary, the authors reported that the total time required for apparent thermalization was 0.35 fm/c. This is within the same order of magnitude as the thermalization scale obtained from accelerator data, already discussed in Section 3.3.1. A discussion of numerical approaches using null evolutions applied to asymptotically AdS problems can be found in [208]. Other recent applications of shock wave collisions in AdS5 to describe phenomenological properties of heavy ion collisions can be found in Refs. [800, 655, 757].

Time-plus-space decompositions have also been initiated, both based on a generalized harmonic evolution scheme [70] and in an ADM formulation [416]. In particular the latter formulation seems very suited for extracting relevant physical quantities for holography, such as the boundary time for the thermalization process discussed in Section 3.3.1.

Evolutions of BHs deformed by a scalar field in AdS_5_ have been presented in Ref. [70]. The evolution leads the system to oscillate in a (expected) superposition of quasi-normal modes, some of which are nonlinearly driven. On the boundary, the dual CFT stress tensor behaves like that of a thermalized ${\mathcal N = 4}$ super-Yang-Mills fluid, with an equation of state consistent with conformal invariance and transport coefficients that match holographic calculations *at all times*. Similar conclusions were reached in Ref. [417], where the numerical scheme of Ref. [207, 208] was used to study the isotropization of a homogeneous, strongly coupled, non-Abelian plasma by means of its gravity dual, comparing the time evolution of a large number of initially anisotropic states. They find that the linear approximation seems to work well even for initial states with large anisotropies. This unreasonable effectiveness of linearized predictions hints at something more fundamental at work, perhaps a washing out of nonlinearities close to the horizon. Such effects were observed before in asymptotically flat spacetimes, for example the already mentioned agreement between ZFL (see Section 5.3) or close limit approximation predictions (see Section 5.2) and full nonlinear results.

Also of interest for accelerator physics, and the subject of intense work in recent years, are holographic descriptions of jet-quenching, i.e., the loss of energy of partons as they cross strongly coupled plasmas produced in heavy ion collisions [2, 199, 200]. Numerical work using schemes similar to that of Refs. [207, 208] have been used to evolve dual geometries describing the quenches [143, 144, 204]; see also [319] for numerical *stationary* solutions in this context.

Another development within the gauge/gravity duality that gained much attention, also discussed in Section 3.3.1, is related to condensed matter physics. In asymptotically AdS spacetimes, a simple theory, say, with a scalar field minimally coupled to the Maxwell field and to gravity admits RN-AdS as a solution. Below a critical temperature, however, this solution is unstable against perturbations of the scalar field, which develops a tachyonic mode. Since the theory admits another set of charged BH solutions, which have scalar hair, it was suggested that the development of the instability of the RN-AdS BHs leads the system to a hairy solution. From the dual field theory viewpoint, this corresponds to a phase transition between a normal and a superconducting phase. A numerical simulation showing that indeed the spacetime evolution of the unstable RN-AdS BHs leads to a hairy BH was reported in [562]. Therein, the authors performed a numerical evolution of a planar RN-AdS BH perturbed by the scalar field and using Eddington-Finkelstein coordinates. A particular numerical scheme was developed, adapted to this problem. The development of the scalar field density is shown in Figure 21. The initial exponential growth of the scalar field is eventually replaced by an approach to a fixed value, corresponding to the value of the scalar condensate on the hairy BH.

Finally, the gauge/gravity duality itself may provide insight into turbulence. Turbulent flows of CFTs are dual to dynamical BH solutions in asymptotically AdS spacetimes. Thus, urgent questions begging for answers include how and when do turbulent BHs arise, and what is the (gravitational) origin of Kolmogorov scaling observed in turbulent fluid flows. These problems are just now starting to be addressed [185, 12, 13].

### Applications in cosmological settings

Some initial applications of NR methods addressing specific issues in cosmology have been reviewed in the *Living Reviews* article by Anninos [36], ranging from the Big Bang singularity dynamics to the interactions of GWs and the large-scale structure of the universe. The first of these problems — the understanding of cosmological singularities — actually motivated the earlier applications of NR to cosmological settings, cf. the *Living Reviews* article [88]. The set of homogeneous but *anisotropic* universes was classified by Bianchi in 1898 into nine different types (corresponding to different independent groups of isometries for the 3-dimensional space). Belinskii, Khalatnikov and Lifshitz (BKL) proposed that the singularity of a generic inhomogeneous cosmology is a “chaotic” spacelike curvature singularity, and that it would behave asymptotically like a Bianchi IX or VIII homogeneous cosmological model. This is called BKL dynamics or mixmaster universe. The accuracy of the BKL dynamics has been investigated using numerical evolutions in Refs. [87, 558, 662], and the BKL sensitivity to initial conditions in various references (see for instance Ref. [89]). For further details, we refer the reader to Ref. [88].

More recently, NR methods have been applied to the study of bouncing cosmologies, by studying the evolution of adiabatic perturbations in a nonsingular bounce [801]. The results of Ref. [801] show that the bounce is disrupted in regions of the universe with significant inhomogeneity and anisotropy over the background energy density, but is achieved in regions that are relatively homogeneous and isotropic. Sufficiently small perturbations, consistent with observational constraints, can pass through the nonsingular bounce with negligible alteration from nonlinearity.

In parallel, studies of “bubble universes”, in which our universe is one of many nucleating and growing inside an ever-expanding false vacuum, have also been made with NR tools. In particular, Refs. [765, 764] investigated the collisions between bubbles, by computing the cosmological observables arising from bubble collisions directly from the Lagrangian of a single scalar field.

Applications of NR in more standard cosmological settings are still in their infancy, but remarkable progress has been achieved. One of these concerns the impact of cosmic inhomogeneities on the value of the cosmological constant and the acceleration of the universe. In other words, how good are models of homogeneous and isotropic universes — the paradigmatic Friedmann-Lemaître-Robertson-Walker (FLRW) geometry — when we know that our universe has structure and is inhomogeneous?

**Figure 22:**
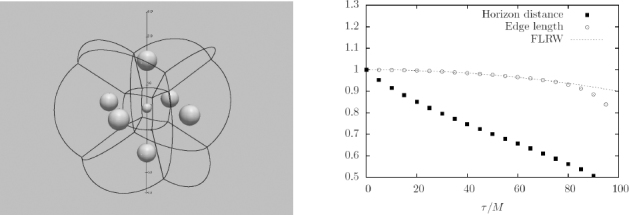
*Left*: Elementary cells for the 8-BH configuration, projected to **R**^3^. The marginal surface corresponding to the BH at infinity encompasses the whole configuration. Note that the 8 cubical lattice cells are isometric after the conformal rescaling. *Right*: Several measures of scaling in the eight-BH universe, as functions of proper time *τ*, plotted against a possible identification of the corresponding FLRW model (see Ref. [86] for details). All the quantities have been renormalized to their respective values at *τ* = 0. Images reproduced with permission from [86], copyright by IOP. All rights reserved.

Studies of this (long-standing, see for instance Ref. [520]) question within NR have considered the evolution of BH lattices (the BHs mimicking strong, self-gravitating inhomogeneities) [86, 805]. In Ref. [86] the authors explicitly constructed and evolved a three-dimensional, fully relativistic, eight-BH lattice with the topology of *S*^3^. The puncture locations in that work projected down to *R*^3^ are shown in the left panel of Figure 22 (one of the punctures is projected out to infinity, see Ref. [86] for further details). The evolution of this 8-BH configuration is summarized in the right panel of Figure 22, showing the (minimal) proper distance between neighbouring surfaces and the proper length of each cell’s edges. These quantities are then compared against a reference FLRW closed model with spatial slices of spherical topology. The comparison procedure is not straightforward, but adopting the procedure of Ref. [86] it yields good agreement.

The effects of local inhomogeneities have been investigated in Ref. [804] using different initial data, describing an expanding inhomogeneous universe model composed of regularly aligned BHs of identical mass. The evolution of these initial data also indicates that local inhomogeneities do not significantly affect the global expansion law of the universe, despite the fact that the inhomogeneities themselves are extremely nonlinear [804, 805]. Similar conclusions were reached in Ref. [834], where the ADM formalism is used to develop a practical scheme to calculate a proposed domain averaging effect in an inhomogeneous cosmology within the context of numerical large-scale structure simulations. This study finds that in the weak-field, slow-motion limit, the proposed effect implies a small correction to the global expansion rate of the universe. In this limit, their simulations are always dominated by the expanding underdense regions, hence the correction to the energy density is negative and the effective pressure is positive. The effects of strong gravity in more general scenarios are yet to be understood [834]. For an earlier NR code developed to address inhomogeneous cosmologies see Ref. [426].

More complex NR codes aimed at understanding cosmological evolutions are currently being developed. NR simulations of large scale dynamical processes in the early universe have recently been reported [345]. These take into account interactions of dark matter, scalar perturbations, GWs, magnetic fields and turbulent plasma. Finally, Ref. [837] considers the effect of (extreme) cosmological expansion on the head-on collision and merger of two BHs, by modelling the collision of BHs in asymptotically dS spacetimes.

## Conclusions

*“Somewhere, something incredible is waiting to be known.”* 22

Einstein’s theory of general relativity celebrates its 100th anniversary in 2015 as perhaps the most elegant and successful attempt by humankind to capture the laws of physics.

Until recently, this theory has been studied mostly in the weak-field regime, where it passed all experimental and observational tests with flying colors. Studies in the strong-field regime, in contrast, largely concerned the mathematical structure of the theory but made few and indirect connections with observation and experiment. Then, a few years ago, a phase transition in the field of strong gravity occurred: on one hand, new experimental efforts are promising to test gravity for the first time in the strong field regime; on the other hand, a new tool — numerical relativity — has made key breakthroughs opening up the regime of strong-field gravity phenomena for accurate modelling. Driven by these advances, gravitation in the strong-field regime has proven to have remarkable connections to other branches of physics.

With the rise of numerical relativity as a major tool to model and study physical processes involving strong gravity, decade-old problems — brushed aside for their complexity — are now tackled with the use of personal or high-performance computers. Together with analytic methods, old and new, the new numerical tools are pushing forward one of the greatest human endeavours: understanding the universe.

## Acronyms

ADM: Arnowitt-Deser-Misner
(A)dS: (Anti-)de Sitter
AH: Apparent horizon
BH: Black hole
BSSN: Baumgarte-Shapiro-Shibata-Nakamura
CFT: Conformal field theory
EOB: Effective one-body
GHG: Generalized Harmonic Gauge
EM: Electromagnetism, Electromagnetic
GR: General relativity
GW: Gravitational wave
IBVP: Initial-boundary-value problem
LHC: Large Hadron Collider
LIGO: Laser Interferometric Gravitational-Wave Observatory
NR: Numerical relativity
NS: Neutron star
PDE: Partial differential equation
PN: Post-Newtonian
PPN: Parametrized Post-Newtonian
QNM: Quasi-normal mode
RN: Reissner-Nordström
SMT: String/M theory
ZFL: Zero-frequency limit

## Notation and conventions

*D*: Total number of spacetime dimensions (we always consider one timelike and *D* − 1 spacelike dimensions).
*L*: Curvature radius of (A)dS spacetime, related to the (negative) positive cosmological constant A in the Einstein equations (*G*_μν_ + Λ*g*_μν_ = 0) through *L*^2^ = (*D* − 2)(*D* − 1)/(2|Λ|).
*M*: BH mass.
*a*: BH rotation parameter.
*R*_S_: Radius of the BH’s event horizon in the chosen coordinates.
*ω*: Fourier transform variable. The time dependence of any field is ∼ *e*^−*iωt*^. For stable spacetimes, Im(*ω*) < 0.
*s*: Spin of the field.
*l*: Integer angular number, related to the eigenvalue *A*_lm_ = *l*(*l* + *D* − 3 of scalar spherical harmonics in *D* dimensions.
*a*, *b*, …, *h*: Index range referred to as “early lower case Latin indices” (likewise for upper case indices).
*i*, *j*, …, *v*: Index range referred to as “late lower case Latin indices” (likewise for upper case indices).
*g*_*αβ*_: Spacetime metric; greek indices run from 0 to *D* − 1.
$\Gamma _{\beta \gamma}^\alpha$: $= {1 \over 2}{g^{\alpha \mu}}({\partial _\beta}{g_{\gamma \mu}} + {\partial _\gamma}{g_{\mu \beta}} - {\partial _\mu}{g_{\beta \gamma}})$, Christoffel symbol associated with the spacetime metric *g*_αβ_.
${R^\alpha}_{\beta \gamma \delta}$: $= {\partial _\gamma}\Gamma _{\delta \beta}^\alpha - {\partial _\delta}\Gamma _{\gamma \beta}^\alpha + \Gamma _{\gamma \rho}^\alpha \Gamma _{\delta \beta}^\rho - \Gamma _{\delta \rho}^\alpha \Gamma _{\gamma \beta}^\rho$, Riemann curvature tensor of the *D*-dimensional spacetime.
∇_*α*_: *D*-dimensional covariant derivative associated with $\Gamma _{\beta \gamma}^\alpha$.
*γ*_*ij*_: Induced metric, also known as first fundamental form, on (*D* − 1)-dimensional spatial hypersurface; latin indices run from 1 to *D* − 1.
*K*_*ij*_: Extrinsic curvature, also known as second fundamental form, on (*D* − 1)-dimensional spatial hypersurface.
$\Gamma _{jk}^i$: (*D* − 1)-dimensional Christoffel symbol associated with *γ*_ij_.
${{\mathcal R}^i}_{jkl}$: (*D* − 1)-dimensional Riemann curvature tensor of the spatial hypersurface.
*D*_*i*_: (*D* − 1)-dimensional covariant derivative associated with $\Gamma _{jk}^i$.
*S*^*n*^: *n*-dimensional sphere.

## Footnotes

1
http://relativity.livingreviews.org/Articles/subject.html

2 That is, the existence of mass currents in opposite spatial directions (in between the horizons) and at relativistic velocities in the centre of energy frame.

3 This bound has an interesting story. Kip Thorne, and others after him, attribute the conjecture to Freeman Dyson; Freeman Dyson denies he ever made such a conjecture, and instead attributes such notion to his 1962 paper [157], where he works out the power emitted by a binary of compact objects. (We thank Gary Gibbons and Christoph Schiller for correspondence on this matter.)

4 Such fragmentation may however not be a counterexample to the spirit of the cosmic censorhip conjecture, if black strings do not form in generic collapse situations. One hint that this may indeed be the case comes from the Dyson-Chandrasekhar-Fermi instability of higher-dimensional cylindrical matter configurations [174]: if cylindrical matter configurations are themselves unstable it is unlikely that their collapse leads to black strings.

5 In the context of quantum gravity, it has ben shown that including a fundamental minimal length, a solution exists in which an interior regular solution is matched to the exterior Kerr metric. Such configuration, however, is a “regularized” BH rather than a description of stars [706].

6 Here, $[{{D - 1} \over 2}]$ denotes the integer part of ${{D - 1} \over 2}$.

7 Numerical solutions of axially symmetric, rotating NSs in GR have been derived by several groups (see [330] and the *Living Reviews* article [728], and references therein), and in some cases their codes have been made publically available [729, 117]. These solutions are used to build initial data for NR simulations of NS-NS and BH-NS binary inspiral and merger.

8 If matter or energy is present, there is a stress-energy tensor which is also perturbed, ${T_{\mu \nu}} = T_{\mu \nu}^{(0)} + \delta {T_{\mu \nu}}$. If ${T_{\mu \nu}}$ describes a fluid, its perturbation can be described in terms of the perturbations of the thermodynamic quantities

9 Strictly speaking, the signature represents the signs of the eigenvalues of the metric: *g*_*αβ*_ has 1 negative and *D* − 1 positive eigenvalues even when the timelike coordinate is replaced in terms of one or two null coordinates.

10 Decompositions in terms of null foliations have to our knowledge not been studied yet, although there is no evident reason that speaks against such an approach.

11 … but beware! For many realistic types of matter, novel effects — such as shocks — can hamper an efficient evolution. These have to be handled with care and would require a review of its own.

12 We remark that the dimensional reduction here discussed is different from Kaluza-Klein dimensional reduction [463, 283], an idea first proposed about one century ago, which in recent decades has attracted a lot of interest in the context of SMT. Indeed, a crucial feature of Kaluza-Klein dimensional reduction is spacetime compactification, which does not occur in our case.

13 There is a length-squared factor multiplying the exponential which we set to unity.

14 Note that Ref. [700] chooses *z* instead of *y*.

15 Generalizations to higher dimensions have been studied for Einstein gravity [836] and for five-dimensional Gauss-Bonnet gravity [814].

16 “Petrov type D” is a class of algebraically special spacetimes, which includes in particular the Schwarzschild and Kerr solutions.

17 The electric and magnetic part of the Weyl tensor may be interpreted as describing tidal effects and differential dragging of inertial frames, respectively, which has been employed to visualize spacetimes in terms of so-called “Frame-Drag vortexes” and “Tidal Tendexes” [593, 579, 578, 833].

18
http://relativity.livingreviews.org/Articles/subject.html

19 Cosmic censorship does not apply to cosmological singularities, i.e., Big Bang or Big Crunch.

20 The *D* = 5 analysis in [701, 700] has been revised in yet unpublished work, obtaining better agreement with the linear results in [273], cf. Ref. [694].

21 Floating orbits would manifest themselves in observations by depleting the inner part of accretion disks of stars and matter, and modifying the emitted gravitational waveform.

22 This quote, usually attributed to Carl Sagan, was published in *Seeking Other Worlds* in Newsweek magazine (April 15, 1977), a tribute to Carl Sagan by D. Gelman, S. Begley, D. Gram and E. Clark.
